# Soft Hypoxia‐Adaptive Bioelectronics Integrating PEDOT:PSS/Polydopamine/Enzyme Biocomposites for Closed‐Loop Therapeutics of Chronic Wounds

**DOI:** 10.1002/advs.76904

**Published:** 2026-07-30

**Authors:** Songrui Liu, Bowen Yang, Cao Qi, Zhijie Zhou, Haochen Zou, Anjum Qureshi, Xiao Zhao, Ting Li, Li Gao, Ye Tao, Gang Song, Pingqiang Cai, Zheng Li, Zhiyuan Liu, Dianpeng Qi, Ting Wang, Lianhui Wang

**Affiliations:** ^1^ State Key Laboratory of Flexible Electronics (LoFE) & Jiangsu Key Laboratory of Smart Biomaterials and Theranostic Technology Institute of Advanced Materials (IAM) Nanjing University of Posts and Telecommunications Nanjing P. R. China; ^2^ SUNUM Nanotechnology Research and Application Center Sabanci University Tuzla Istanbul Turkey; ^3^ Translational Mechanomedicine Lab Medical School Nanjing University Nanjing P. R. China; ^4^ College of Biomedical Engineering & Instrument Science Zhejiang University Hangzhou P. R. China; ^5^ Shenzhen Institute of Advanced Technology Chinese Academy of Sciences Shenzhen P. R. China; ^6^ School of Chemistry and Chemical Engineering Harbin Institute of Technology Harbin P. R. China

**Keywords:** adaptive, hypoxia, multiplex biosensing, soft electronics, wound healing

## Abstract

Adaptive bioelectronics that autonomously adjust to environmental changes represent an emerging paradigm, enabling reliable operation across diverse conditions. Hypoxic microenvironments are prevalent across numerous pathological conditions including chronic wounds, tumors, and ischemic tissues, creating a fundamental challenge: oxygen‐dependent enzymatic biosensors fail precisely when monitoring is most critical, while oxygen deficiency simultaneously impairs tissue regeneration. We present a soft wireless Hypoxia‐Adaptive Sensing and Therapeutic (HAST) system that maintains reliable biosensing functionality in oxygen‐deficient environments through integrated oxygen management. Using engineered poly(3,4‐ethylenedioxythiophene):poly(styrene sulfonate) (PEDOT:PSS)/polydopamine (PDA)/enzyme biocomposites, HAST integrates multiplexed biosensing (glucose, uric acid, lactate) with dual‐oxygen provision: wound exudate‐triggered dissolved oxygen generation restores biosensor functionality while electrical stimulation promotes vascular regeneration for sustained tissue oxygenation. This hypoxia‐adaptive design achieves about 10‐fold biosensor sensitivity enhancement under oxygen‐deficient conditions while promoting tissue repair. In preclinical diabetic wound models, HAST enabled accurate continuous monitoring with ∼30% accelerated wound closure, demonstrating environment‐adaptive bioelectronics for precision medicine in oxygen‐deficient pathological conditions.

## Graphical abstract

Hypoxia‐adaptive bioelectronics based on poly(3,4‐ethylenedioxythiophene):poly(styrene sulfonate) (PEDOT:PSS)/polydopamine (PDA)/enzyme biocomposites couple O_2_‐centric biosensing with embedded O_2_ therapy to address oxygen‐deficient chronic wounds. Local O_2_ provision restores enzyme‐based metabolite sensing, while catalase‐mediated oxygen generation and electrical stimulation reshape the wound microenvironment, promoting vascular regeneration and accelerated tissue recovery.

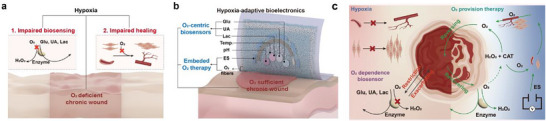

## 1 Introduction

Adaptive sensing systems represent a paradigm shift in bioelectronics, enabling sensors to autonomously adjust their performance in response to dynamic environmental changes [1, 2, 3]. Inspired by biological sensory systems—such as photoreceptor adaptation to varying light intensities, mechanoreceptor tuning to pressure gradients, and cochlear adaptation to sound pressure levels and frequency selectivity—artificial adaptive sensors have been developed for neuromorphic vision [4, 5, 6, 7], tactile robotics [8, 9], acoustic signal processing [10] and strain‐adaptive biosensing that compensates for mechanical deformation in wearable and implantable devices [11]. These systems achieve robust performance by dynamically recalibrating sensitivity, gain, or detection range to match external stimuli, thereby maintaining accuracy across fluctuating conditions. However, a critical gap persists: current adaptive sensing architectures focus exclusively on external physical stimuli (light, pressure, sound, mechanical deformation) while overlooking adaptation to biochemical microenvironmental changes, particularly oxygen availability—a fundamental limitation that undermines their utility in hypoxic biomedical applications (left panel in Figure S1).

**FIGURE 1:**
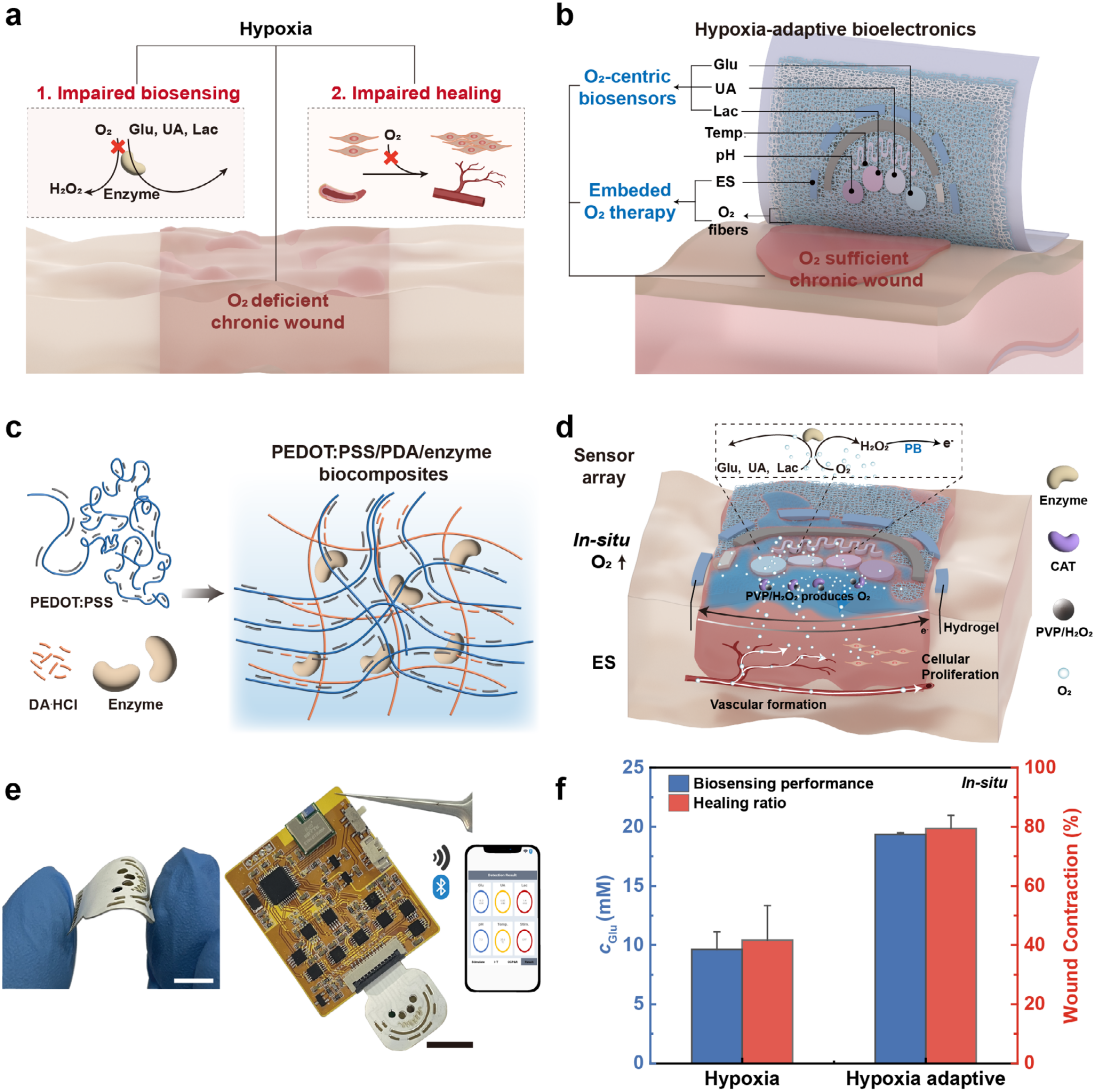
Hypoxia‐adaptive bioelectronics for integrated sensing and therapy. (a) Dual challenges posed by hypoxic microenvironments: impaired O_2_‐dependent enzymatic biosensing (right) and compromised tissue healing processes (left). (b) Schematic of the hypoxia‐adaptive bioelectronics system featuring O_2_‐centric biosensors for metabolite detection (Glu, UA, Lac, Temp., pH) and embedded O_2_ therapy through direct oxygen generation and ES‐induced vascular regeneration. (c) PEDOT:PSS/PDA/enzyme biocomposite design enabling increased enzyme loading, enhanced conductivity, and improved enzyme activity for reliable biosensing under hypoxic conditions. (d) Cross‐sectional illustration showing the integrated system's hypoxia working mechanism: biosensors for real‐time monitoring, in situ O_2_ production through catalytic decomposition, and ES promoting cellular proliferation and vascular formation. (e) Photographs of the flexible HAST biochip demonstrating mechanical flexibility and wireless connectivity. Scale bar: 1 cm. (f) Performance comparison showing biosensor sensitivity enhancement and wound contraction improvement under oxygen provision vs. hypoxic conditions, demonstrating dual functionality in restoring sensing accuracy and therapeutic efficacy. Data are presented as mean ± SD (*n* = 3).

Hypoxic microenvironments, characterized by severely depleted oxygen levels (pO_2_ of ∼0.125–0.5 mg L^−1^ compared to 0.75–1.25 mg L^−1^ in healthy tissues) [12], are prevalent in chronic wounds [13, 14, 15], solid tumors [16, 17], ischemic tissues [18, 19], and mitochondrial disorders [20, 21]. Unlike stable laboratory conditions where adaptive sensors excel, these pathological settings present a unique challenge: oxygen scarcity directly impairs the sensing mechanism itself. Conventional enzymatic biosensors—the gold standard for metabolite detection due to their specificity and low detection limits—rely on oxygen as an obligate co‐substrate for electrochemical transduction. In hypoxic conditions, glucose oxidase (GOx), lactate oxidase (LOx), and uricase (UOx) require oxygen for catalytic turnover, yet oxygen depletion restricts their linear response ranges, destabilizes signals, and dramatically reduces sensitivity [22] (left panel in Figure 1a). This creates a paradoxical negative feedback loop: hypoxia impairs sensor performance, preventing accurate diagnosis, while the sensors themselves consume residual oxygen during enzymatic reactions, further exacerbating hypoxia and stalling tissue repair [23, 24, 25] (right panel in Figure 1). No existing adaptive sensing platform addresses this bidirectional sensor‐microenvironment interaction.

Chronic diabetic wounds exemplify this challenge. Vascular insufficiency and inflammatory cell oxygen consumption generate profound hypoxia, while metabolic dysregulation amplifies healing deficits [3]. Biomarkers such as glucose (Glu, indicating glycemic control and bacterial metabolism) [26, 27], lactate (Lac, reflecting tissue hypoxia and metabolic stress) [28], and uric acid (UA, signaling inflammation and oxidative damage) [29] provide critical diagnostic information—yet their oxygen‐dependent enzymatic detection fails precisely when needed most. Specifically, oxidase enzymes require dissolved oxygen as the terminal electron acceptor; under hypoxic conditions, this enzymatic reaction is severely compromised, rendering the biosensors unreliable. Previous hypoxia‐addressing strategies, including oxygen delivery (photosynthetic microalgae) [23, 24], chemical generation (catalase (CAT)/peroxide, perfluorocarbons) [30, 31], and nanobubble delivery systems [32, 33]) and biological approaches such as vascular restoration through electrical stimulation (ES), growth factors, or angiogenic stimulation [34, 35], improved oxygenation or promoted healing, but lacked integration with biosensing (right panel in Figure S1). An integrated hypoxia‐adaptive sensing platform—capable of dynamically restoring its own sensing microenvironment while simultaneously providing therapeutic intervention—has not yet been realized.

**FIGURE 2:**
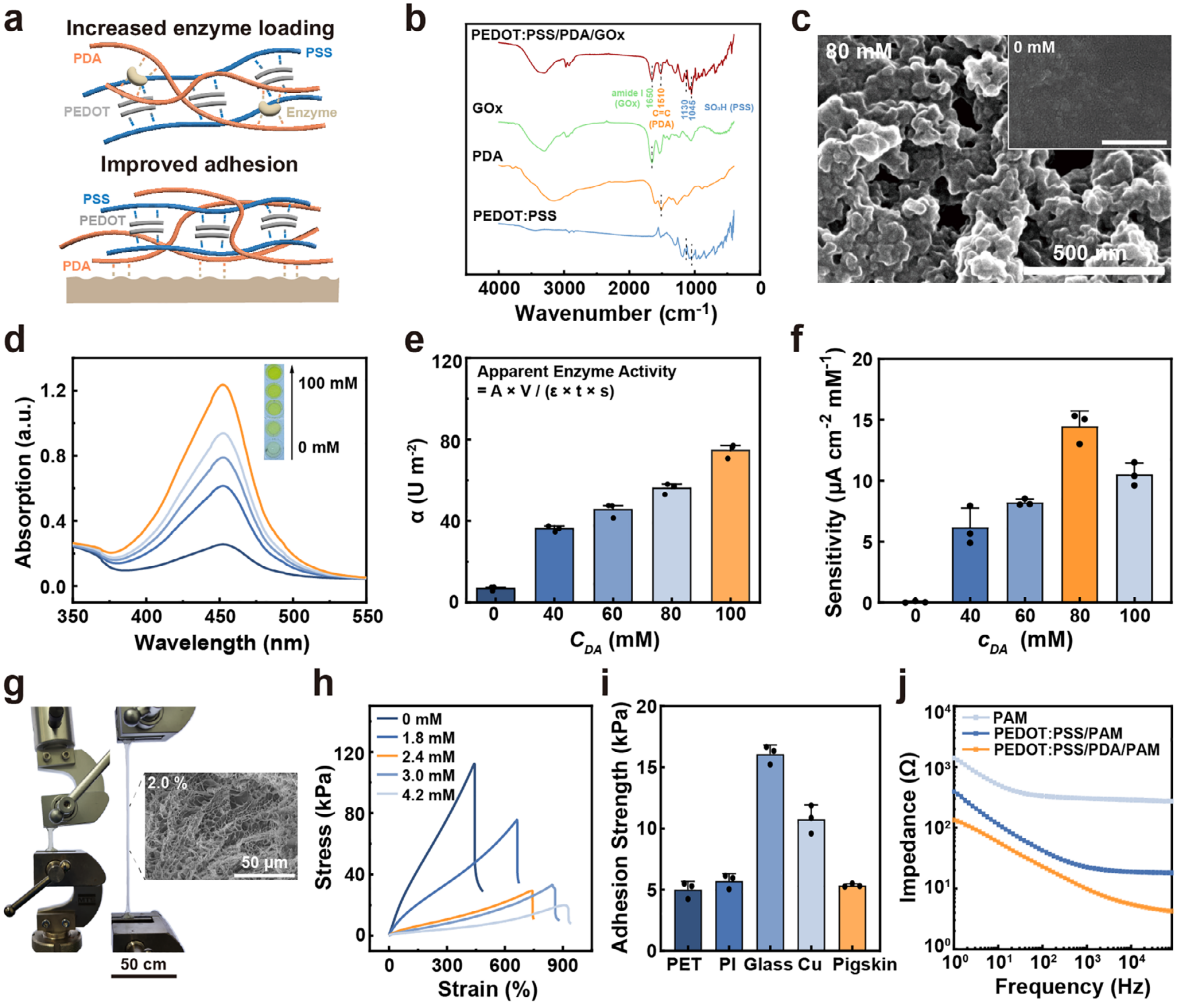
Design and characterization of PEDOT:PSS/PDA/enzyme biocomposites for HAST system. (a) Schematic illustrating DA‐mediated enhancements in enzyme loading capacity and adhesion performance. (b) FT‐IR spectra of GOx, PEDOT:PSS, PDA, and PEDOT:PSS/PDA/GOx nanocomposite, confirming successful incorporation of all components. (c) SEM images of biocomposites showing morphological differences: 80 mm DA concentration reveals a 3D rough surface with enhanced porosity that facilitates biosensing (left), while 0 mm DA concentration shows smooth morphology (right, inset). (d) TMB colorimetric assay results showing UV absorbance at 450 nm after 15 min of enzymatic reaction with varying DA concentrations (0, 40, 60, 80, 100 mm) (with the inset showing colorimetric changes over the 15‐min reaction period). Increased absorbance with higher DA content indicates enhanced enzyme loading and activity within the biocomposites. (e) Quantified apparent enzyme activity of biocomposite‐immobilized electrodes at different DA concentrations. Data are presented as mean ± SD (*n* = 3). (f) Sensitivity of PEDOT:PSS/PDA biosensor at 0 V with varied DA ratios, showing optimal performance at 80 mm DA. Data are presented as mean ± SD (*n* = 3). (g) Digital images of the PEDOT:PSS/PDA/PAM hydrogels under 0% strain (left panel) and 700% strain (right panel), demonstrating their excellent stretchability enabled by stress dissipation through the porous structure of the hydrogels, as shown in the inset. (h) Stress–strain curves of PEDOT/PDA/PAM hydrogels with different DA concentration, illustrating the softening effect of DA on the hydrogel. (i) Adhesion strength of the hydrogel (prepared with 2.4 mm of DA) to various substrates. Data are presented as mean ± SD (*n* = 3). (j) Electrochemical impedance of PAM, PEDOT:PSS/PAM and PEDOT:PSS/PDA/PAM.

In this work, we present a wireless Hypoxia‐Adaptive Sensing and Therapeutic (HAST) biochip that bridges this gap by introducing the first adaptive biosensing system responsive to oxygen‐limited microenvironments (Figure 1b). HAST shows systematic adaptivity to hypoxia environment, which actively modulates its local oxygen availability through wound exudate‐triggered catalytic oxygen generation, breaking the sensor‐hypoxia negative feedback loop. The system integrates three synergistic components: (1) PEDOT:PSS/PDA/enzyme biocomposites for multiplexed enzymatic biosensing, where PEDOT:PSS provides electrochemical conductivity and PDA enables high‐capacity enzyme immobilization (Figure 1c); (2) CAT/H_2_O_2_‐mediated oxygen generation triggered by wound fluid contact, restoring sensor functionality and tissue repair; and (3) ES to promote angiogenesis and accelerate healing (Figure 1d). This architecture enables about 10‐fold enhanced sensitivity for Glu, Lac, and UA detection in oxygen‐depleted environments. Integrated with soft wireless flexible printed circuitry, HAST achieves real‐time monitoring and therapeutic control, accelerating wound closure by ∼30% in diabetic models while maintaining accurate biomarker monitoring in hypoxia. By addressing biochemical microenvironment adaptation—a critical advance beyond physical‐stimuli‐responsive sensors—this platform enables broad applications in chronic wounds, tumor monitoring, ischemic tissue management, and inflammatory disorders.

## 2 Design and Fabrication of the HAST System

The HAST system was engineered to couple multiplexed O_2_‐dependent biosensing (Glu, UA, and Lac) with dual oxygen restoration strategies, thereby maintaining sensing accuracy while promoting vascular regeneration under hypoxic conditions (Figure 1b,d). The platform integrates enzymatic sensors for Glu, UA, and Lac with immediate O_2_ generation through enzyme‐catalyzed H_2_O_2_ decomposition and sustained O_2_ supply through ES‐induced vascularization. To enable this multifunctionality, we developed PEDOT:PSS/PDA/enzyme biocomposites as the core transduction materials. Fabricated using a layer‐by‐layer assembly (Figure S2), the system comprise four functional elements: (i) a flexible polymer substrate with patterned gold electrodes, (ii) PEDOT:PSS/PDA/enzyme‐modified electrode arrays for multiplex biomarker detection, (iii) a PEDOT:PSS/PDA/polyacrylamide (PEDOT:PSS/PDA/PAM) hydrogel interface for ES delivery, featuring two pairs of electrodes (six hydrogel units total, three per side) arranged concentrically around the 13‐mm diameter wound area, and (iv) a sandwich‐structured electrospun fiber encapsulating catalase (CAT) and polyvinylpyrrolidone‐hydrogen peroxide (PVP‐H_2_O_2_) for controlled O_2_ release. Notably, the localized oxygen generation within the wound exudate ensures both optimal biosensor activity and efficient tissue uptake.

The biochip is integrated with a flexible printed circuit board (which connects to the pre‐designed pins of the patch via a Board‐to‐Board interface) that supports biosignal acquisition, wireless data transmission, and programmable voltage modulation for ES (Figure 1e and Figure S3). Photographs of the assembled HAST flexible biochip device highlight its conformability and mechanical robustness bending deformation, collectively illustrating flexibility and wearability (Figure 1e). The circuit diagram (Figure S4) reveals the system's three functional modules: the underlying infrastructure (gray) including power management, Bluetooth wireless transmission, and data processing chips; the oxygen restoration and ES control unit (blue) for regulated oxygen generation and ES delivery; and the multimodal sensing interface (red) for biosignal acquisition and conversion. These modules operate in closed‐loop coordination with biosensor outputs guiding oxygen restoration and ES delivery in real‐time. Video S1 demonstrates the wireless operation of the system in vivo, where the biochip adheres securely to wound tissue, maintains functionality during normal mouse activity, and provides continuous biomarker monitoring alongside responsive ES therapy. The flexible design ensures biocompatibility and wearability, enabling uninterrupted monitoring and adaptive treatment throughout the healing process (Figure 1f).

## 3 Characterization of PEDOT:PSS/PDA/Enzyme Biocomposites for the HAST Biochip

To realize multifunctional performance in hypoxic environments, we engineered two PEDOT:PSS/PDA‐based material systems for biosensing and ES, respectively. For biosensors, PEDOT:PSS/PDA/enzyme biocomposites were fabricated through in situ polymerization of dopamine (DA) within PEDOT:PSS solutions containing target enzymes. This in situ approach is particularly advantageous for enzyme immobilization: as DA monomers polymerize, enzymes are progressively encapsulated within the forming PDA network (Figure S5), providing a protective three‐dimensional matrix that shields enzymes from denaturation while enabling high enzyme loading. The high affinity of PDA for proteins during this encapsulation process simultaneously establishes strong interactions between components (Figure 2a), preserving catalytic activity while preventing enzyme leaching. For the ES module, the incorporation of PDA in the PEDOT:PSS hydrogel matrix enhanced interfacial adhesion to biological tissues and improved both ionic conductivity of the PEDOT:PSS through its positively charged amine groups. The DA concentrations for the biosensing interface and ES hydrogel were independently optimized according to their respective performance requirements. Together, these properties rendered the biocomposite highly suitable for fragile wound environments requiring both robust electrochemical biosensing and ES performance.

Efficient enzyme immobilization is crucial for achieving high biosensing capabilities. Efficient enzyme loading in PEDOT:PSS/PDA matrix was confirmed by Fourier Transform Infrared Spectroscopy (FT‐IR), typical peaks of enzymes (1650 cm^−1^, amide I bands) [36, 37], PDA (1510 cm^−1^, C═C bonds from the indole structure) [38], and PEDOT:PSS (1130 and 1045 cm^−1^, S‐O stretching of PSS's SO^3−^) (Figure 2b) [39]. Scanning electron microscopy (SEM) images of PEDOT:PSS/PDA/enzyme biocomposites revealed that increasing DA concentration significantly enhanced surface roughness and promoted porous microstructures, which was attributed to increased enzyme loading and PDA accumulation (Figure 2c and Figure S6). At a typical DA concentration of 80 mm, a well‐defined three‐dimensional (3D) porous structure emerged, providing abundant active sites for biosensing applications.

Moreover, enzyme activity of biocomposite‐modified flexible electrodes was quantitatively evaluated by a colorimetric assay in which GOx catalyzed glucose oxidation, producing H_2_O_2_ that oxidized 3,3’,5,5’‐Tetramethylbenzidine (TMB) to yield a blue chromogenic signal, which was then converted to yellow upon addition of 2 m H_2_SO_4_ and measured at 450 nm. For varied DA concentration, color development accelerated with increasing DA content, confirming enhanced enzyme loading with higher DA concentrations. Without DA, minimal coloration occurred (Figure 2d and Figure S7). Based on total absorbance changes at 15 min following reaction termination via 2 m H_2_SO_4_, quantified enzyme activities increased from 6.9, 36.2, 45.5, 56.0, to 74.6 U m^−2^ as DA concentration increased from 0 to 100 mm, respectively (Figure 2e), demonstrating increased enzyme loading with higher DA concentrations. In control experiments omitting GOx, varying DA concentration (40–100 mm) resulted in negligible absorbance changes (Figure S8), confirming that the colorimetric signal originates exclusively from enzymatic activity rather than from intrinsic properties of PDA or DA. Electrochemical measurements corroborated this trend and showed that increasing DA concentration from 0 to 80 mm significantly enhanced sensitivity from negligible response (<0.1 µA cm^−2^ mm ^−1^) to 14.46 µA cm^−2^ mm ^−1^ (based on Polyethylene Terephthalate (PET)/Au electrode), consistent with colorimetric results (Figure 2f and Figure S9). The enhanced sensitivity originates from increased enzyme loading enabled by PDA‐assisted in situ encapsulation, whereas excessive PDA formation (100 mm DA) hinders interfacial electron transfer despite further increasing enzyme loading. Consequently, 80 mm DA represents the optimal balance between enzyme loading and electron transfer for the multiplex biosensing array. Importantly, PDA‐modified electrodes (80 mm DA) demonstrated excellent long‐term enzyme retention with negligible leaching over 7 days at 37°C (Figure S10a) and maintained >88% activity after 7 days of refrigerated storage at 4°C (Figure S10b) and 37°C (Figure S10c), confirming superior stability for practical biosensor applications.

Regarding mechanical properties, PDA incorporation significantly enhances the tissue‐interfacing characteristics of PEDOT:PSS‐based hydrogels by reducing Young's modulus, improving stretchability, and enhancing interfacial adhesion. Specifically, with a DA concentration of 2.4 mm (balancing stretchability and modulus), the hydrogel achieved 750% stretchability, substantially higher than PEDOT:PSS‐based hydrogels. Simultaneously, PDA softened the hydrogel, reducing Young's modulus from 37 to 3 kPa (Figure 2g,h and Note S1), well below typical epidermal Young's modulus (10 to 500 kPa) [40], ensuring conformability and minimizing tissue irritation. SEM imaging revealed that PDA incorporation created a porous structure, explaining the enhanced stretchability and softness by enabling homogeneous stress dissipation during deformation (Figure 2g and Figure S11). The in situ formation of the PDA network significantly enhanced interfacial adhesion, as measured by lap shear tests [41, 42], through catechol‐mediated bonding mechanisms (hydrogen bonding, *π–π* interactions, and covalent cross‐linking), increasing adhesion strength ∼6‐fold vs. PEDOT:PSS/PAM and ∼3‐fold vs. pure PAM (Figure S12). The resulting hydrogel demonstrated adequate adhesion to various substrates, including pig skin (∼5 kPa), suitable for soft wound environments without causing tissue damage (Figure 2i, Figure S13, and Note S2). This combination of high stretchability, appropriate softness, and moderate adhesion enables reliable electrode‐wound interfacing essential for effective ES.

Additionally, PEDOT:PSS/PDA hydrogels demonstrated significantly lower interfacial impedance compared to PEDOT:PSS/PAM and pure PAM hydrogels (Figure 2j), attributed to the positively charged amine groups in PDA that enhance ionic conductivity and interfacial contact [43, 44]. Importantly, this low impedance was maintained under mechanical stress, with Electrochemical Impedance Spectroscopy (EIS) showing negligible impedance changes after cyclic stretching (Figure S14), indicating excellent electrical stability and preserved conductive pathways during repeated mechanical deformation. This improved electrical interfacing was validated through Electrocardiogram (ECG) monitoring, where PEDOT:PSS/PDA/PAM electrodes showed higher signal‐to‐noise ratios compared to control hydrogels (Figure S15 and Note S3). In summary, the optimized PEDOT:PSS/PDA nanocomposite synergistically combines high enzyme loading capacity, enhanced conductivity, and tissue‐compatible mechanical properties, where PEDOT:PSS providing electrical/ionic conductivity and PDA enabling enzyme immobilization and tissue adhesion. This synergistic effect contributes to effective multifunctional performance in the HAST biochip with reliable biosensing, localized oxygen production, and ES‐mediated vascular regeneration.

## 4 Validation of the Hypoxia Adaptive Ability of the HAST Biochip

To validate the hypoxia‐adaptive capacity of the HAST biochip, we systematically assessed the impact of oxygen deficiency and the efficacy of our oxygen‐embedded therapy (in vitro) on biosensor function and cellular behavior. Considering that dissolved oxygen provides better accessibility for both enzymatic reactions and cellular uptake compared to gaseous oxygen, we designed a wound exudate‐triggered oxygen generation module using water‐soluble polyvinyl alcohol (PVA) as the responsive matrix to produce dissolved O_2_ directly within the wound fluid environment.

**FIGURE 3:**
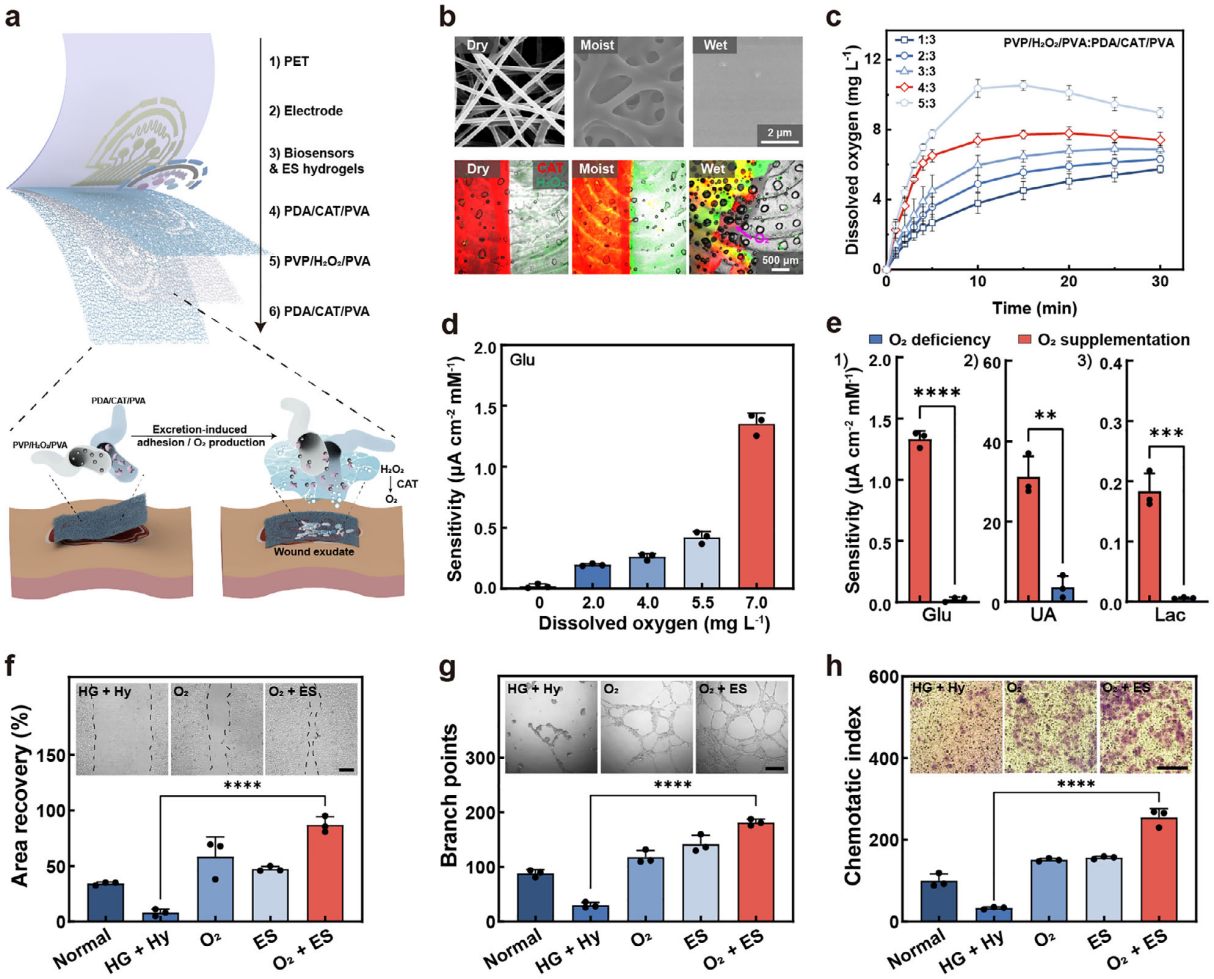
In vitro evaluation of the HAST biochip's biosensing and therapeutic performance under simulated hypoxic conditions. (a) Schematic illustrating the layered structure of the HAST system and the process by which its O_2_‐provision layers release O_2_ through wound fluid‐driven dissolution. (b) Electrospinning oxygen‐generation process. The upper layer shows a series of SEM images illustrating the morphological changes of oxygen‐provision electrospun fibers in dry, moist, and wet states over time. The lower layer presents confocal fluorescence images of oxygen‐producing electrospun fibers on mouse wound sites, illustrating the dissolution process and oxygen release, with CAT (red) and PVP/ H_2_O_2_ (green) labeling. (c) Characterization of O_2_ release from O_2_ provision fibers. With the amount of PDA/CAT/PVA fixed at 30 mg, the effect of varying the amount of PVP/H_2_O_2_/PVA on the oxygen generation rate was optimized. At a 4:3 ratio, the release rate reached 7–8 mg L^−1^ within 10 min and sustained this level for up to 30 min at 37°C. (d) Influence of dissolved O_2_ concentration on Glu sensor sensitivity, demonstrating increased sensitivity with higher O_2_ levels. Data are presented as mean ± SD (*n* = 3). (e) Comparison of sensitivity differences in O_2_‐dependent biosensors (Glu, UA, and Lac) under simulated hypoxic conditions and with O_2_ provision by HAST biochip, demonstrating significant enhancement in sensing performance across multiple analytes. Data are presented as mean ± SD (*n* = 3). Statistical significance was determined using an unpaired two‐tailed Student's *t*‐test. ^**^ *p* < 0.01; ^***^ *p* < 0.001; ****p < 0.0001. Quantification of HUVEC cell migration (f), tube formation (g), and chemotaxis (h) under different treatment conditions, demonstrating the synergistic effects of O_2_ provision and ES on cellular behavior. Insets show the representative images of the HUVEC scratch assay (24 h), tube formation assay (6 h), and chemotaxis assay (24 h) under HG + Hy, O_2_, and O_2_ + ES conditions. Scale bar: 200 µm. Data are presented as mean ± SD (*n* = 3). Statistical significance was determined using one‐way ANOVA followed by Dunnett's multiple‐comparisons test. ^**^ *p* < 0.01; ^***^ *p* < 0.001; ^****^ *p* < 0.0001.

Our oxygen‐embedded module adopts a multilayer sandwich structure with upper and lower PDA/CAT/PVA layers enclosing PVP/H_2_O_2_/PVA a middle layer (Figure 3a upper panel). Upon wound application, the wound exudate penetrates the hydrophilic PVA matrix, dissolving the fibers and releasing encapsulated CAT and PVP/H_2_O_2_ precursors into the wound fluid. Their catalytic reaction generates dissolved O_2_, alleviating hypoxic microenvironments while restoring biosensor performance and supporting tissue healing (Figure 3a, bottom). Importantly, the excess of CAT design ensures complete H_2_O_2_ decomposition, preventing interference with sensing elements and avoiding oxidative tissue stress.

To validate this wound‐responsive mechanism, we characterized fiber morphology under three conditions: ambient air, high humidity (∼80% RH), and direct liquid contact (Figure 3b upper panel) and confirmed dissolution dynamics of the fibers. SEM images revealed a structural transformation from intact fibrous structures in ambient conditions to fiber swelling at high humidity, and finally to homogeneous film‐like dissolution upon liquid contact. The rapid dissolution was confirmed by contact angle measurements, where water droplets were completely absorbed within 25 s as the contact angle decreased from 121° to 0° (Figure S16).

Using wrinkled skin replicas and fluorescently labeled components (sulfo‐cyanine 5 NHS for CAT, 6‐carboxyfluorescein for PVP/H_2_O_2_), we first investigated the behavior of each layer under different moisture conditions. Confocal fluorescence imaging revealed that PDA/CAT/PVA and PVP/H_2_O_2_/PVA fibers showed enhanced dissolution and component release with increasing humidity levels, progressing from minimal release in dry conditions to extensive spreading along tissue interfaces under high humidity and droplet contact conditions (Figure S17). When both fiber types were combined and exposed to simulated wound exudate, visible bubble formation occurred at the fiber interface, confirming the sequential dissolution‐release‐catalysis mechanism where PVA dissolution releases the encapsulated components, allowing CAT and H_2_O_2_ to react and generate oxygen (lower panel in Figure 3b).

To quantify oxygen release from the CAT‐mediated H_2_O_2_ decomposition reaction, we conducted quantitative oxygen release assays (solution‐based tests) with a portable dissolved oxygen meter (JPBJ‐610L). We first investigated the oxygen generation rate by fixing the PVP/H_2_O_2_/PVA layer mass at 10 mg in 30 mL water while varying the PDA/CAT/PVA layer amount. Results showed that oxygen release rates plateaued when the PDA/CAT/PVA layer reached 30 mg or higher, indicating sufficient CAT availability for complete H_2_O_2_ conversion (Figure S18). We then optimized the mass ratio of PVP/H_2_O_2_/PVA to PDA/CAT/PVA layers (with PDA/CAT/PVA fixed at 30 mg) and found that a 4:3 ratio achieved optimal O_2_ release of 7–8 mg L^−1^ within 10 min at 37°C (Figure 3c). Further increasing the H_2_O_2_ proportion resulted in excess oxygen escaping into the air due to oxygen saturation limitations. The excess CAT eliminates potential sensor interference from residual peroxide, with Glu sensors showing consistent baseline and response currents before and after oxygen generation (Figure S19).

Having established the oxygen generation mechanism, we next examine the effects of oxygen availability on biosensor operation through systematic electrochemical studies. Using GOx as a model enzyme on PEDOT:PSS/PDA/GOx modified Au/ thermoplastic polyurethane (Au/TPU) fiber electrodes, we first examined sensing performance under controlled oxygen conditions by simulating hypoxic microenvironments through nitrogen bubbling and regulated air introduction. As dissolved O_2_ levels increased from 0 to 7 mg L^−1^, biosensing sensitivity rose dramatically from 0.02 to 1.35 µA cm^−2^ mm ^−1^, demonstrating the critical oxygen dependence of the enzymatic transduction signal of biosensors (Figure 3d and Figure S20). We then evaluated the performance of our complete oxygen‐generating membrane system for Glu, UA, and Lac biosensing. Under nitrogen‐bubbled hypoxic conditions (30 min deoxygenation), biosensor performance was severely compromised. However, when the designed O_2_ generation membrane system was activated to provide localized oxygen generation, it restored the performance and enhanced the sensitivity. PEDOT:PSS/PDA/enzyme biosensors showed enhanced sensitivity by 44‐fold for Glu (0.03 to 1.33 µA cm^−2^ mm ^−1^), 9.5‐fold for UA (3.29 to 31.1 cm^−2^ mm ^−1^), and 18‐fold for Lac (0.01 to 0.18 µA cm^−2^ mm ^−1^) (Figure 3e and Figure S21). These results confirm that the integrated O_2_‐generating membrane effectively restores O_2_‐dependent biosensor performance in hypoxic environments such as chronic wounds.

Beyond impacting biosensing performance, hypoxic microenvironments also inhibit normal cellular physiological activities, impeding chronic wound healing [23, 24]. We first examined the expression of hypoxia‐inducible factor‐1α (HIF‐1α), a key transcription factor that regulates cellular response to hypoxia, under different conditions. Compared to the normal state (Figure S22a), exposure to hyperglycemia and hypoxia (HG + Hy, 33 mm Glu) led to HIF‐1α overexpression, indicating cellular hypoxia (Figure S22b). Importantly, our O_2_ provision layer (PDA/CAT/PVA and PVP/H_2_O_2_/PVA fibers) notably reduced HIF‐1α expression in HUVECs under HG + Hy conditions, demonstrating its effectiveness in reversing cellular hypoxia (Figure S22c and Note S4).

In addition to direct O_2_ production, previous research has shown that ES can facilitate vascular reconstruction and restore normal blood O_2_ supply to wound sites by enhancing Phosphoinositide 3‐Kinase (PI3K) secretion and decreasing Phosphatase and tensin homologue (PTEN) expression [45, 46, 47, 48]. We investigated the synergistic effects of O_2_ provision (O_2_), ES, and their combination (O_2_ + ES) on cellular activities using HUVECs as a model. The ES intensity (100 mV/mm) was selected based on reported values in the literature [45, 49]. Proliferation rates under O_2_, ES, and O_2_ + ES, evaluated by Cell Counting Kit‐8 (CCK8) testing, were significantly higher than in the control (HG + Hy) group (Figure S23a,b and Note S5). To further assess cell proliferation and migration under these conditions, we conducted cell scratch assays. Compared to the control group, O_2_ provision and ES significantly enhanced HUVEC migration and induced faster gap closure (Figure 3f and Figure S24).

To evaluate angiogenesis potential, we performed tube formation assays and transwell assays. The tube formation assay, simulating angiogenic capacity in vitro, showed significantly more tube branches in the O_2_ + ES treatment compared to the control group (Figure 3g and Figure S25). The transwell assay further confirmed the enhanced migration capability of HUVECs under O_2_ provision and ES conditions (Figure 3h and Figure S26), which is crucial for angiogenesis. Collectively, these results indicate that while O_2_ provision rapidly alleviates local hypoxia, ES further promotes endothelial cell proliferation, migration, and angiogenesis, thereby facilitating vascular reconstruction and restoring endogenous oxygen supply to the wound microenvironment. Notably, the combined O_2_ + ES treatment consistently outperformed either individual intervention, highlighting the synergistic therapeutic benefits of immediate oxygen supplementation and ES‐mediated vascular remodeling for chronic wound repair.

## 5 Evaluation of the Multiplex Biosensing Capability In vitro

To enable comprehensive monitoring of chronic wound microenvironments, the HAST biochip integrates multiplexed sensing of physical parameters, including Glu (as an indicator of tissue metabolism and bacterial colonization), UA (as a marker of tissue damage and inflammation) [50], and Lac (indicating tissue hypoxia) [28], incorporated with pH and temperature (temp.). Together, these targets provide an accurate and real‐time physicochemical fingerprint of wound status, with pH and temperature additionally serving as indicators of infection and wound inflammatory activity [50, 51, 52].

Stable electrode operation was achieved through hydrophobic octadecyl trichlorosilane (OTS) (4%) modification of Au electrodes, followed by shadow‐mask oxygen plasma to restore electroactivity at defined regions. Cyclic Voltammetry (CV) and EIS results confirmed suppressed electrochemical activity (through significantly reduced peak currents and increased charge‐transfer resistance) after OTS treatment and localized recovery after plasma patterning (Figure S27a,b). Ag/AgCl reference electrodes were fabricated by electrochemical chlorinating evaporated Ag (200 nm) films (CV treatment in 0.1 m KCl and 0.01 m HCl mixed solution), exhibited stable potential outputs for > 3000 s (Figure S28).

**FIGURE 4:**
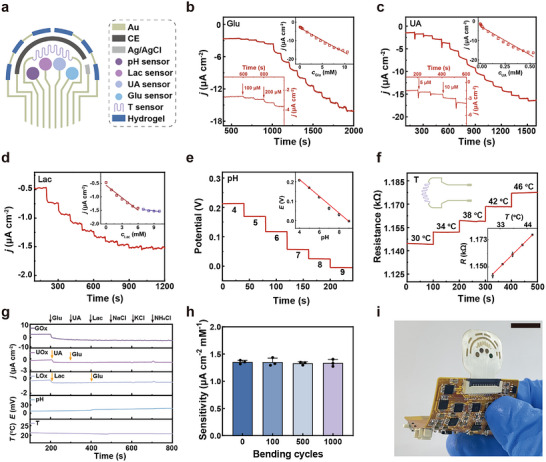
Multiplex biosensing performance and biocompatibility evaluation of the HAST system. (a) Schematic illustration of the integrated multiplex sensing system. (b–f) Sensing performance and linear calibration curves (insets) for Glu (0.1–10.6 mm) (b), UA (5–530 µm) (c), Lac (0.1–6 mm, and 7–10 mm) (d), pH (4–9) (e), and temperature (30^ο^C–46^ο^C) (f). Calibrations were performed in phosphate buffer (0.1 m PBS, pH 7.5) using pure analyte solutions to determine sensor sensitivity, linear range, and detection limits under controlled conditions. (g) Selectivity evaluation of the multifunctional sensing system. Sequential addition of potential interferents for: Glu (target: 200 µm Glu; interferents: 500 µm UA, 10 mm Lac, and 10 mm each of NaCl, KCl, NH_4_Cl); UA sensor (target: 20 µm UA; interferents: 10 mm each of Glu, Lac, NaCl, KCl, NH_4_Cl); Lac sensor (target: 200 µm Lac; interferents: 500 µm UA, 10 mm Glu, and 10 mm each of NaCl, KCl, NH_4_Cl); pH and temperature sensors response to various metabolites and electrolytes (10 mm Glu, 500 µm UA, 10 mm Lac, and 10 mm each of NaCl, KCl, NH_4_Cl). (h) Mechanical stability of Glu Sensor. Bar Chart: >95% initial sensitivity retained after 1000 bends, showing robust durability for wearables. Data are presented as mean ± SD (*n* = 3). (i) Photographs of the flexible HAST system under bending, including the bending of the circuit board. Scale bar: 1 cm.

The multiplex sensing system is shown in Figure 4a. For wound biomarker detection, the Glu sensor exhibited a linear range of 0.1–10.6 mm with a limit of detection (LOD) of 0.018 mm (Figure 4b). UA sensor spanned 0.005–0.53 mm, achieving an LOD of 0.0015 mm (Figure 4c). Lac sensing exhibited two linear ranges: 0–6 mm and 7–10 mm, with an LOD of 0.026 mm (Figure 4d). These enzymatic sensors were calibrated in simulated wound fluid to ensure accurate performance under physiologically relevant conditions (Figure S29). Despite the complex protein‐ and electrolyte‐rich environment, the sensors retained ∼90% of their sensitivity while maintaining excellent linearity, with detection ranges effectively covering the physiological and pathological concentrations encountered in chronic wounds (Table S1). The polyaniline‐based pH sensor modified Au/TPU fiber exhibited a sensitivity of 43 mV/pH (Figure 4e), while the serpentine Au microwire temperature sensor utilizing showed a sensitivity of 2.08 Ω/°C (Figure 4f). Enzymatic activity was largely unaffected by ±2°C temperature changes (Figure S30a), whereas pH shifts between 6.5 and 8.5 altered calibration slopes (Figure S30b), underscoring the need for integrated physical readouts to correct metabolite sensing under varying wound conditions. The multiplex sensor system also showed strong selectivity against various interfering electrolytes and metabolites commonly present in wound fluid, ensuring reliable sensing performance in complex wound environments (Figure 4g).

Moreover, the mechanical robustness of multiplex sensing electrodes was validated by continuous bending cycles (1000 cycles, bending diameter = 10 mm) with no significant resistance drift (Figure S31a). Following this durability test, the electrodes maintained consistent Glu sensing performance, confirming their long‐term reliability (Figure 4h). Beyond post‐cycling stability, the sensor maintains stable Glu detection under real‐time bending conditions at different curvatures (bending diameters = 30, 20, 10 mm, tested via Linear Sweep Voltammetry (LSV)), showing nearly identical responses at each Glu concentration (400 and 800 µm) across different bending states (Figure S31b,c). The integrated wireless circuit system maintained stable data transmission under mechanical deformations (Figure 4i), confirming the reliability of daily wear.

For clinical applications, biocompatibility is crucial for wound monitoring devices. Biocompatibility was confirmed through the cytotoxicity response of all HAST components. HUVECs retained >95% viability after 48 h exposure to device components (Figure S32a and Note S6). Furthermore, hemolysis rates remained below 0.5% for all materials, satisfying the <5% clinical threshold (Figure S32b and Note S7). No skin irritation was observed after a 6 h in vivo application on human skin (Figure S32c), collectively demonstrating the excellent biocompatibility of the HAST biochip for wound monitoring and therapeutic applications. Additionally, we evaluated potential reactive oxygen species (ROS)‐related effects by examining tissue sections treated with different electrospun patches. In comparison to normal tissue and the combined CAT + H_2_O_2_ group, which incorporated our O_2_ provision module with PDA/CAT/PVA and PVP/H_2_O_2_/PVA layers, the H_2_O_2_‐only group showed significantly stronger ROS response. By contrast, the CAT + H_2_O_2_ group maintained ROS levels comparable to those observed in normal tissue (Figure S33a,b and Note S8). These findings demonstrate that the presence of excess CAT efficiently consumes H_2_O_2_ at the wound site, thereby mitigating harmful oxidative stress. This mechanism ensures safe oxygen release while preserving tissue integrity, a prerequisite for the reliable integration of oxygen‐generating modules into closed‐loop bioelectronics wound healing platforms.

## 6 Systematic Integration and Evaluation of Multiplex Biosensing and Therapeutic Efficacy in Chronic Wounds

**FIGURE 5:**
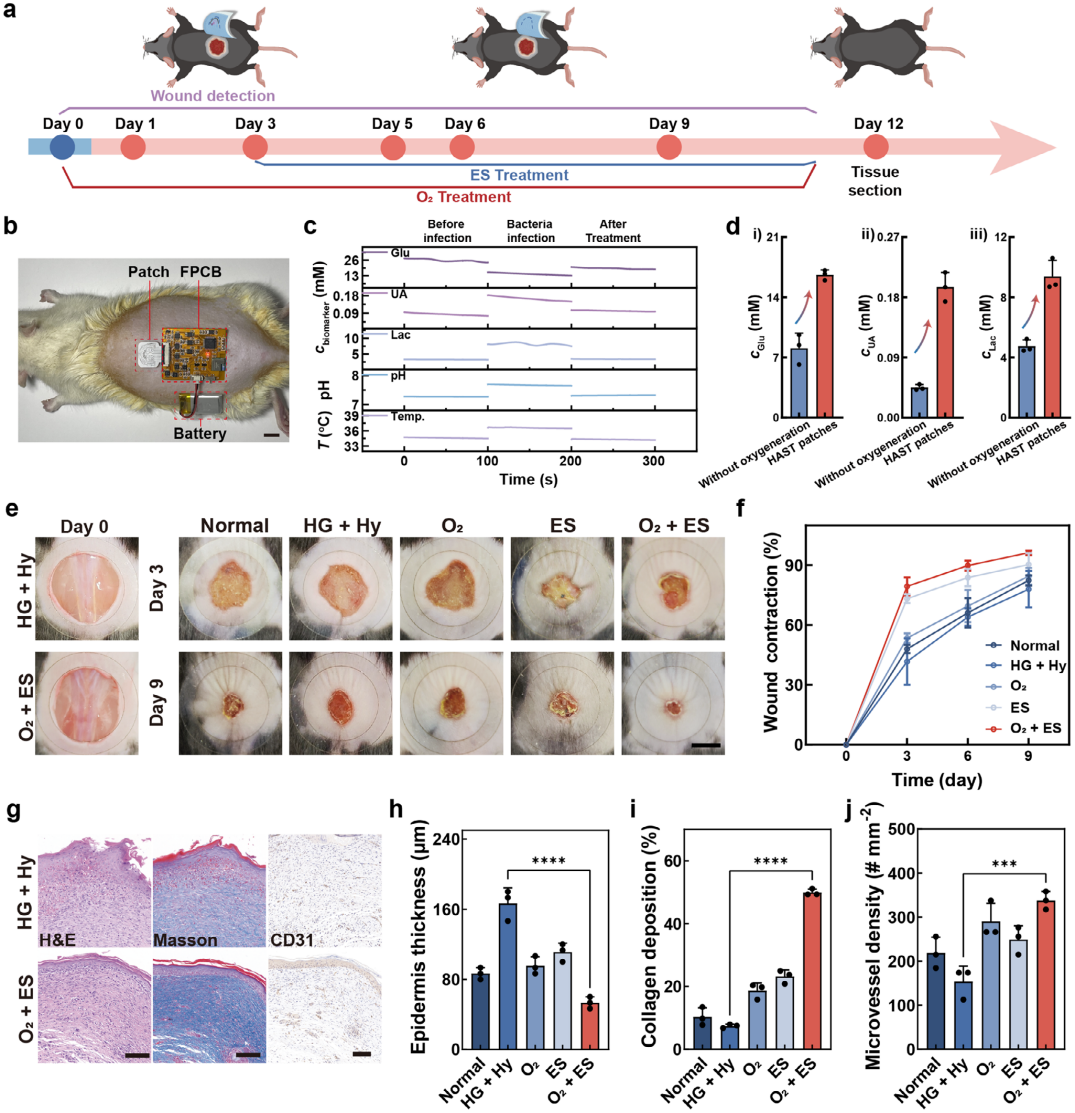
In vivo validation of HAST biochip for wound monitoring and therapy in diabetic mouse model. (a) Schematic illustration of diabetic wound modeling, detection, and treatment protocol. (b) Photographs showing HAST biochip application on dorsal wound of diabetic rat for real‐time in situ monitoring and therapy, Scale bar: 1 cm. (c) In situ monitoring of wound exudate biomarker concentrations by HAST biochip before infection, during bacterial infection, and after treatment. (d) Comparison of O_2_‐dependent enzyme sensor performance (Glu, UA, and Lac) between HAST biochips and patches without O_2_ provision, showing >1.9‐fold sensitivity enhancement with O_2_ supplementation in diabetic chronic wounds. Data are presented as mean ± SD (*n* = 3). (e) Representative wound images under different treatments (O_2_ provision, ES, and O_2_ + ES) at days 0, 3, and 9 post‐operation (*n* = 3). Scale bar: 5 mm. (f) Wound closure rates assessed every three days, demonstrating superior healing with O_2_ + ES combined treatment compared to single treatments. Data are presented as mean ± SD (*n* = 3). (g) Representative H&E, Masson's trichrome, and CD31 immunostaining images of diabetic wounds with and without O_2_ + ES treatment, showing enhanced re‐epithelialization, collagen deposition, and vascularization in the treated group (*n* = 3). Scale bar: 100 µm. Quantitative analysis of (h) epidermis thickness, (i) collagen deposition, and (j) microvessel density in normal mice, untreated diabetic mice, and diabetic mice under O_2_, ES, and O_2_ + ES treatments. Data are presented as mean ± SD (*n* = 3). Statistical significance was determined using one‐way ANOVA followed by Dunnett's multiple‐comparisons test. ^**^ *p* < 0.01; ^***^ *p* < 0.001; ^****^ *p* < 0.0001.

To evaluate the efficacy of the HAST biochip in chronic wound diagnosis and treatment, we established a diabetic mouse model (using C57BL/6 male mice) through high‐fat diet feeding combined with streptozotocin (STZ) administration. Mice with plasma Glu levels of over 16.7 mm after 10 days of STZ injection were considered diabetic [23, 24]. A full‐thickness skin wound (13 mm diameter) was created on the dorsum of each mouse (Figure 5a). To demonstrate in vivo operation, the HAST biochip was attached to a diabetic rat wound with its biosensing, O_2_ provision layers, and ES electrodes facing the wound bed (Figure 5b). The system's flexible design—integrating a conformal biosensing/therapeutic patch (17 × 20 mm), flexible FPCB (42 × 35 mm) for signal processing with a programmable control interface, and a miniaturized battery—enabled wireless monitoring and therapy during unrestricted animal activity. The control interface enabled programmable adjustment of ES parameters (voltage: 0–1300 mV, cycle number, on/off state) based on real‐time biomarker monitoring, providing a foundation for closed‐loop therapeutic intervention (Figure S34). The stimulation electrode was positioned in close proximity to the wound without direct immersion in wound fluid, thereby preventing hydrogel swelling while maintaining effective ES. The oxygen‐generating patch was replaced once daily during the in vivo experiments to provide repeated oxygen supplementation throughout the treatment period. Future iterations could implement pathology‐responsive oxygen delivery guided by wound biomarkers.

Before checking the diagnosis and therapeutic ability, we first check the attachment of devices, especially the O_2_ provision film on diabetic wounds. On a real mouse wound, PVA‐based O_2_ provision films transform into transparent, well‐conformed films within 30 min upon wound exudate contact (Figure S35). This transformation demonstrates PVA's ultra‐hydrophilic nature and controlled dissolution, which is the basis for O_2_ provision in the wound bed. Importantly, this wound biofluid‐triggered adhesion enables secure attachment during wear while allowing atraumatic removal via saline irrigation, minimizing tissue damage during dressing changes. Meanwhile, the interfacial adhesion contributes to stress dissipation between the electrospun layer and HAST substrate, ensuring mechanical stability of the integrated system (Figure S36).

We first assessed metabolic fluctuations linked to dietary intake and affects wound exudate composition (Figure S37). Fasting increased Lac levels while decreasing Glu and UA levels, consistent with systemic metabolite shifts [53]. The decrease in body temperature was attributed to reduced metabolic activity during fasting‐induced hypothermia [54]. Refeeding restored Glu and UA levels, recovered due to restored metabolic homeostasis and nutrient availability [27], demonstrating HAST biochip's capability for real‐time wound monitoring.

Infected wound monitoring further revealed characteristic biochemical signatures (Figure 5c). Post‐infection wounds exhibited elevated levels of UA, Lac, pH, and temperature, alongside >35% glucose depletion. These changes reflect typical infection characteristics: increased UA and temperature indicating inflammation [50], elevated pH suggesting bacterial colonization [51], and reduced Glu resulting from bacterial consumption [27]. The increased Lac can be attributed to two factors: enhanced glycolysis under hypoxic conditions, in which cells shift to anaerobic metabolism [28, 55]; and bacterial metabolism in the infected environment [27].

Moreover, considering the impact of hypoxia on sensing performance, we compared HAST biochips with conventional non‐oxygenating control patches on diabetic chronic wounds. The oxygen‐generating HAST biochips demonstrated increased sensitivity > 1.9‐fold, demonstrating that the integrated O_2_ generation effectively mitigated the hypoxic conditions that typically impair O_2_‐dependent biosensors (Figure 5d). These results demonstrate that integrated oxygen provision directly overcomes hypoxia‐induced sensing degradation and is advantageous for hypoxic wound monitoring.

To verify the therapeutic effects of HAST biochips, we conducted in vivo experiments on mouse models with O_2_, ES (pulsed DC: ±1.3 V, 100 mV/mm, 50 ms polarity reversal, 1‐second on/off cycles, 30 min daily), and O_2_ + ES treatments administered once daily, using untreated diabetic wounds (Diabetic) and normal wounds (Normal) as controls. The O_2_ + ES group showed significantly accelerated healing, achieving 79% wound closure by day 3, compared to 53% (O_2_), 73% (ES), and 41% (Diabetic). By day 9, O_2_ + ES treatment reached 96% closure, comparable to normal healing. While untreated diabetic wounds showed significantly delayed healing after 12 days, both O_2_ + ES and normal groups achieved complete closure (Figure 5e,f and Figure S38).

Histological analysis of wounds confirmed the synergistic effects of O_2_+ES therapy. Hematoxylin and Eosin (H&E) staining revealed that O_2_ + ES treatment significantly promoted re‐epithelialization (12 days), with epithelial thickness (53 ± 7 µm) approaching that of healthy epidermis (24 ± 7 µm) compared to other groups [31]. In contrast, the Diabetic, O_2_, and ES groups showed thinner epidermis (167 ± 17 µm, 96 ± 10 µm, and 111 ± 10 µm, respectively) (Figure 5g,h and Figure S39). Masson staining demonstrated increased dense collagen deposition group (Figure 5g,i and Figure S39), while CD31 immunostaining revealed enhanced vascularization in O_2_, ES, and most prominently O_2_ + ES‐treated wounds compared to the Diabetic group (Figure 5g,j and Figure S39). Additionally, Ki67 staining showed markedly elevated cell proliferation in O_2_ and O_2_ + ES groups (Figure S40a,b and Note S9).

Together, these findings demonstrate that the HAST biochip system uniquely integrates multiplex wound monitoring with active therapeutic capability. The oxygen provision module not only restores O_2_‐dependent biosensor fidelity but also synergizes with ES to accelerate wound closure through enhanced cell proliferation, collagen deposition, and angiogenesis under hypoxic conditions. This dual diagnostic‐therapeutic functionality represents a significant advance toward closed‐loop bioelectronic management of chronic wounds.

## 7 Conclusions

We present a soft, wireless HAST system addressing the dual challenges of chronic wound microenvironments: impaired healing and compromised enzymatic biosensor accuracy. The system integrates multiplex metabolite sensing (Glu, UA, Lac), wound‐exudate‐responsive oxygen delivery, and ES within a modular platform. The oxygen‐generating electrospun fiber restores biosensor function while supporting cellular metabolism, achieving ∼10‐fold sensitivity improvement under hypoxia. Concurrently, ES via PEDOT:PSS/PDA/PAM hydrogels enhances vascularization and tissue regeneration, accelerating wound closure by ∼30% in diabetic models. The modular design—featuring reusable sensor/stimulation components and replaceable oxygen‐generating layers—enables long‐term clinical implementation. Beyond chronic wounds, this integrated approach could extend to other hypoxic pathological conditions including ischemic tissues and tumor microenvironments. By coupling adaptive sensing with responsive therapy in a wireless platform, HAST demonstrates a paradigm for personalized bioelectronics capable of real‐time diagnosis and treatment in challenging physiological environments. Future iterations will aim to further integrate continuous biosensor feedback with automated regulation of oxygen delivery and ES, thereby advancing the current clinician‐in‐the‐loop workflow toward a fully autonomous closed‐loop therapeutic system capable of intelligent adaptive responses to dynamic wound conditions.

## 8 Methods

### 8.1 Materials

Dopamine hydrochloride (DA‐HCl), N,N’‐methylenebisacrylamide (MBA), ammonium persulfate (APS), tetramethylethylenediamine (TMEDA), sodium hydroxide (NaOH), tetrahydrofuran (THF), N,N‐dimethylformamide (DMF), aniline, chloroplatinic acid, potassium ferricyanide (K_3_Fe(CN)_6_), ferric chloride (FeCl_3_), potassium chloride (KCl), octadecyltrichlorosilane (OTS), polyvinylpyrrolidone (PVP), polyvinyl alcohol (PVA), Glucose oxidase (GOx) and catalase (CAT) were purchased from Aladdin Technology Co., Ltd. (Shanghai, China). Acrylamide (AM) was purchased from Merck Ltd. (Shanghai, China). Poly (3,4‐ethylenedioxythiophene) (styrenesulfonate) (PEDOT:PSS) (PH1000) was purchased from Heraeus. Uricase (UOx) was purchased from Yien Chemical Technology Co., Ltd. (Shanghai, China). Lactate oxidase (LOx) was purchased from Macklin Biochemical Co., Ltd. (Shanghai, China). ECG electrode pads (915T55) were purchased from Shenfeng Medical Supplies Co., Ltd. (Shanghai, China).

### 8.2 Fabrication of HAST System

The HAST system was fabricated through a series of well‐coordinated steps. Initially, a 20% TPU solution was prepared using a 1:1 mass ratio of THF and DMF, followed by electrospinning at 15 kV for 3 h to form a uniform membrane on silicone release paper. This TPU electrospun film was then transferred onto a PET substrate, where a 100 nm Au layer was deposited via thermal evaporation and subsequently treated with OTS. Next, UV laser cutting was employed to pattern the TPU/Au layer, and the sensor area was thoroughly cleaned using a plasma instrument to restore the hydrophilicity of the sensing electrode. The reference electrode was chlorinated to produce Ag/AgCl. Subsequently, the sensor area was modified with Glu, UA, Lac, and pH sensing layers, in addition to a patterned hydrogel electrode. Finally, PVP/H_2_O_2_/PVA and PDA/CAT/PVA electrospun films were fabricated, patterned with a UV laser, and integrated into the system, completing the assembly of the HAST system.

### 8.3 The Preparation Procedures of PEDOT:PSS/PDA/Enzymes

For the enzymatic sensors, a Prussian Blue (PB) layer was electrodeposited onto the gold electrode via cyclic voltammetry (scan rate: 100 mV/s, 10 cycles, −0.2 to 0.6 V vs. commercial Ag/AgCl) in an electrolyte solution containing ferric chloride (FeCl_3_), potassium ferricyanide (K_3_[Fe(CN)_6_]), potassium chloride (KCl), hydrochloric acid (HCl), and chitosan (CS). Subsequently, a bionanocomposite solution was prepared by polymerizing DA in the presence of GOx (3 mg/mL), Na_2_PtCl_6_, and PEDOT:PSS. This enzyme‐immobilized composite was drop‐cast onto the PB‐modified electrode, air‐dried, and then encapsulated with Nafion. The fabricated electrodes were stored at 4°C for subsequent measurements. Similar procedures were employed for UOx (3 mg/mL) and LOx (24 mg/mL) based sensors.

### 8.4 pH and Temperature Sensor

For the pH sensor, an aniline solution was prepared containing 0.1 m aniline and 1 m sulfuric acid. A polyaniline layer was deposited on the evaporated gold electrode surface by CV at a scan rate of 100 mV/s through 25 cycles (relative to a commercial Ag/AgCl reference electrode from −0.2 to 1 V).

For the temp. sensor, an Au layer was deposited onto the TPU fibers, followed by UV laser cutting into a serpentine structure to construct the temp. sensing electrodes.

### 8.5 Fabrication of Oxygen‐Provision Electrospun Fibers

For the layered electrospun O_2_‐providing fiber, the top and bottom layers, PDA/CAT/PVA fibers, were fabricated by preparing a mixed solution of 4 mm chloroplatinic acid and 80 mm DA, adjusting it to neutral pH, and adding CAT at 20 mg mL^−1^. This solution was then mixed with a 20% PVA solution in a 1:1 ratio and electrospun at 15 kV. The middle layer, PVP/H_2_O_2_/PVA fibers, was created by incubating PVP (242 mg) with 1 mL of H_2_O_2_ at low temperature in the dark for 8 h. This mixture was subsequently combined with a 20% PVA solution in a 1:1 ratio and electrospun at 15 kV, depositing the fibers onto the surface of the PDA/CAT/PVA fibers.

### 8.6 Fabrication of ES Hydrogel

For the synthesis of the conducting adhesive stimulation hydrogel, DA was first mixed with an NaOH solution (pH = 11) for 20 min in air. Next, PEDOT:PSS was combined with the DA oligomer solution for 10 min to prepare the PEDOT:PSS/PDA composite. AM (2.5 g), APS (10 wt.% initiator), MBA (0.8 wt.% cross‐linker), and TMEDA (0.62 wt.% accelerator) were then mixed with the PEDOT:PSS/PDA solution for 10 min in an ice bath under nitrogen. The resulting solution was poured into a Teflon mold and covered with a glass slide, followed by thermal polymerization at 90°C for 20 min to produce low‐impedance PEDOT:PSS/PDA/PAM hydrogel films.

### 8.7 Enzyme Activity Assay

Enzyme solutions (taking GOx as an example) with different DA addition amounts were immobilized on PET substrates (diameter: 1 cm), then immersed in 3 mL of a solution containing 1.2 mm TMB, 0.02 mg/mL horseradish peroxidase (HRP), and 10 mm Glu for reaction. The reaction was terminated by adding 2 m H_2_SO_4_, and the absorbance at 450 nm was measured using a microplate reader. The apparent enzyme activity of the GOx‐immobilized electrodes was calculated using the following formula:

$$\mathrm{Apparent}\;\mathrm{Enzyme}\;\mathrm{Activity}\;\left(U\;m^{-2})=A\;\times \;V\;\right./\left(\varepsilon \times t\;\times s\right)$$

where A is the difference in absorbance before and after incubation, V is the total volume, ε is the millimolar extinction coefficient (59 for TMB at 450 nm) [56], t is the reaction time, and s is the surface area of the electrode.

### 8.8 Electrochemical Characterization

The multiplex sensing system was characterized to evaluate its sensitivity, stability, and selectivity in solutions of target analytes in phosphate‐buffered saline (PBS, 0.1 m) using an Electrochemical workstation CHI660E (Chenhua, China). For the impedance measurement, the hydrogel films were patterned using a laser cutter. Next, the hydrogel was layered between two Pt electrodes, and the impedance of the hydrogel film was measured with an Electrochemical workstation CHI660E (Chenhua, China) in a frequency range of 10^−1^ to 10^5^ Hz.

### 8.9 In vitro Cell Studies

#### 8.9.1 Cell Culture

HUVECs were obtained from Nanjing Keygentec. HUVECs were incubated in Dulbecco's Modified Eagle Medium (DMEM, Keygentec) containing 10% fetal bovine serum (FBS; Gibco). The cells were cultured at 37°C and 5% CO_2_. Cells were passaged at 70% confluency. For all in vitro studies, cells were cultured using high‐Glu (450 mg/dL) and serum‐free medium under 1% oxygen and 37°C conditions.

#### 8.9.2 Scratch Wound‐Healing Assay

For the scratch wound‐healing assay, cells were cultured in six‐well plates until they reached 85% to 95% confluency (*n* = 3 for each group). A scratch was then made using a 200 µL pipette tip. The cells were washed with PBS, after which serum‐free medium, with or without the HAST system. Optical images were captured using an optical microscope (MI52‐N, Mshot) at regular intervals (12 h). The distances between the two sides of the scratch were measured using ImageJ software. The scratch wound‐healing ratio was calculated using the following formula:

$$\begin{array}{ccc} \mathrm{Wound}\;-\mathrm{healing}\;\mathrm{ratio}\left(\%\right) & & =\left[\left(\mathrm{Initial}\;\mathrm{width}-\mathrm{Width}\;\mathrm{at}\;\mathrm{time}\;t\right)/\right.\\ & & \;\left.\mathrm{Initial}\;\mathrm{width}\right]\times 100\% \end{array}$$

#### 8.9.3 Transwell Migration Assay

Transwell chambers with an 8 µm pore size (Corning) were used for the transwell migration assay. HUVECs were seeded into the upper chamber at a density of 5 × 10^3^ cells per well. The lower chamber contained medium with or without the HAST system. After 24 h, cells remaining on the upper surface of the filter were removed using a cotton swab. Migrated cells on the bottom side of the filter were fixed with 4% paraformaldehyde and stained with a 0.5% crystal violet solution for 1 h. The migrated cells were photographed and counted under an optical microscope.

#### 8.9.4 Tube Formation Assay

For the tube formation assay, 50 µL per well of thawed Matrigel (Corning) was added into a pre‐cooled 96‐well plate and incubated for 1 h at 37°C. Then, HUVECs were seeded with 1 × 10^4^ cells per well into the Matrigel‐coated 96‐well plate, and the HAST system was attached to the wall of the 96‐well plate. To assess the tube formation, the tubes were imaged under an optical microscope and quantified using ImageJ software.

### 8.10 Animal Studies

#### Chronic Wound‐Healing Models

C57BL/6 male mice (8 weeks old) were obtained from Yangzhou University Medical Centre (Yangzhou, China). 4–5 mice were housed in a cage, and a sizzle‐nest was added in the cage for animal welfare. The mice's condition was checked by the experimenter twice a day (9 am and 5 pm). Mice had free access to abundant food and water. All animal experiments were performed under the guidelines set by the Academic Morality and Ethical Committee of Nanjing University of Posts and Telecommunications (No.2024016, Nanjing, China).

STZ‐induced diabetic models: C57BL/6 male mice were fed with high‐sugar feed and maintained under a specific pathogen‐free environment. Briefly, the animals other than those in the control group (*n* = 3) were administered streptozotocin (STZ, 50 mg/kg, Coolaber) mixed in sodium citrate buffer (pH = 4.3, intraperitoneal injection) daily for four consecutive days. After 10 days of the STZ injection, all the treated mice with plasma Glu levels over 16.7 mm under Vierendeel conditions were considered diabetic. Animals were maintained in a diabetic state for the wound healing and sensing experiment.

STZ‐induced diabetic mice were anesthetized with isoflurane (2%–5%) and secured on a sterilized surgical table. The hair on their backs was removed using an electric razor and depilatory cream. The skin was then disinfected with 75% ethanol. A full‐thickness wound was created at the center of the dorsum, removing the epidermis and superficial parts of the dermis. The animals were randomly divided into five groups:1. Normal group (Normal); 2. STZ group (HG + Hy); 3. STZ with electrical stimulation system group (ES); 4. STZ with O_2_ provision film group (O_2_); 5. STZ with HAST system group (O_2_ + ES). Digital images of the wound area were captured on the day of surgery and every other day thereafter. A 13 mm diameter PET ring was placed around the wound as a size reference. The wound area was quantified using ImageJ software by three blinded observers. The HAST biochips were applied to monitor metabolite changes in wound exudate before fasting, after 24 h fasting, and 12 post‐feeding.

Animals were randomly selected for euthanasia on day 12 (maturation stage) post‐surgery. Harvested wound tissues were either fixed in 4% Paraformaldehyde (PFA) in PBS or flash‐frozen in liquid nitrogen for subsequent immunohistology and immunofluorescence analyses.

### 8.11 Wound Infection Model, Creation and Monitoring

To establish animal wound‐infection models, STZ‐induced mice were anesthetized with 2–5% isoflurane and secured on a sterilized surgical table. The hair on their backs was removed using an electric razor and depilatory cream, followed by disinfection of the skin with 75% ethanol. A full‐thickness wound was then created at the center of the dorsum, removing the epidermis and superficial dermis. The wound was secured with a Tegaderm film dressing (Nexcare, 3 m, USA). Subsequently, the wounds were inoculated with a suspension of S. aureus (300 µL) with an initial density of ∼10^8^ CFU mL^−1^. After 1 day, the HAST system was placed over the wound and secured with a Tegaderm film dressing. The dressing was replaced daily with a fresh one. For infected wound monitoring, we tracked the wound exudate composition before infection (day 0), post‐infection (day 2), and after treatment (combined O_2_ and ES treatment after day 3).

#### Histology and Immunohistochemistry

The wound tissues were fixed in 4% paraformaldehyde overnight, embedded in paraffin, and serially sectioned at 5 µm. Hematoxylin and eosin (H&E) staining was performed to measure epidermal thickness in wounded skin. Epidermal thickness was calculated in the wound‐closed region. Masson staining was used to quantify collagen deposition.

For immunohistochemical analysis, after dewaxing and quenching the endogenous peroxidase activity with 3% H_2_O_2_ for 10 min, the section was rinsed with PBS and blocked in 5% BSA for 10 min. The slice was incubated with the primary antibody (mouse anti‐CD31 antibody, 1:100, Servicebio) at 4°C overnight and then treated with horseradish peroxidase (HRP)‐conjugated secondary goat anti‐rat antibody at room temperature for 30 min. The sections were observed and photographed using a microscope connected to a digital camera.

## Author Contributions

**Dianpeng Qi**: validation, Writing – review and editing. **Bowen Yang**: conceptualization, investigation, methodology, validation, writing – review and editing. **Anjum Qureshi**: validation, software. **Pingqiang Cai**: validation, writing – review and editing. **Zhijie Zhou**: methodology, validation. **Gang Song**: writing – review and editing, validation, supervision. **Cao Qi**: writing – original draft. **Haochen Zou**: methodology, validation. **Songrui Liu**: writing – review and editing, methodology, validation, investigation, conceptualization, data curation. **Xiao Zhao**: writing – review and editing, supervision, visualization. **Ting Li**: validation, supervision. **Ye Tao**: writing – review and editing. **Li Gao**: writing – review and editing. **Ting Wang**: writing – review and editing, conceptualization, funding acquisition, supervision, project administration. **Lianhui Wang**: writing – review and editing, funding acquisition. **Zheng Li**: validation, writing – review and editing. **Zhiyuan Liu**: writing – review and editing, validation.

## Conflicts of Interest

The authors declare no conflicts of interest.

## Supporting information




**Supporting File 1**: advs76904‐sup‐0001‐SuppMat.docx.

**Supporting File 2**: advs76904‐sup‐0002‐VideoS1.mp4.

## Data Availability

The data that support the findings of this study are available from the corresponding author upon reasonable request.
