# Supplementary material for: Soft Hypoxia‐Adaptive Bioelectronics Integrating PEDOT:PSS/Polydopamine/Enzyme Biocomposites for Closed‐Loop Therapeutics of Chronic Wounds
Source: Adv Sci (Weinh). 2026 Jul 30:e76904. Online ahead of print. doi: 10.1002/advs.76904 (PMC13423484; doi:10.1002/advs.76904)
Supplement: Supplementary file 1 — Supporting File 1: advs76904‐sup‐0001‐SuppMat.docx. [file ADVS-9999-e76904-s002.docx]

Supporting Information

**Soft hypoxia-adaptive bioelectronics integrating PEDOT:PSS/polydopamine/enzyme biocomposites for closed-loop therapeutics of chronic wounds**

*Songrui Liu^1,7^, Bowen Yang^1,7^,* *Cao Qi^1^, Zhijie Zhou^1^, Haochen Zou^1^, Anjum Qureshi^2^, Xiao Zhao^1^, Ting Li^1^, Li Gao^1^, Ye Tao^1^, Gang Song^1^, Pingqiang Cai^3^, Zheng Li^4^, Zhiyuan Liu^5^, Dianpeng Qi^6^, Ting Wang^1*^, and Lianhui Wang^1^*

^1^State Key Laboratory of Flexible Electronics (LoFE) & Jiangsu Key Laboratory of Smart Biomaterials and Theranostic Technology, Institute of Advanced Materials (IAM), Nanjing University of Posts and Telecommunications, Nanjing 210023, China.

E-mail: iamtingwang@njupt.edu.cn

^2^SUNUM, Sabanci University, Nanotechnology Research and Application Center, Tuzla, 34956, Istanbul, Turkey

^3^Translational Mechanomedicine Lab, Medical School, Nanjing University, Nanjing 210093, China

^4^College of Biomedical Engineering & Instrument Science, Zhejiang University, Hangzhou, 310027, China

^5^Shenzhen Institute of Advanced Technology, Chinese Academy of Sciences, Xueyuan Avenue, Shenzhen, 518055, China
^6^School of Chemistry and Chemical Engineering, Harbin Institute of Technology Harbin 150001, P. R. China

^7^These authors contributed equally to this work.

**Supplementary Notes**

**Supplementary Note 1.** **Mechanical Testing**

The tensile tests were performed using a universal testing machine with a 10 N load cell. The hydrogels were prepared in a dumbbell shape with a narrow width of 4 mm, narrow length of 10 mm, and overall length of 50 mm. The speed of the tensile test was 10 mm min^−1^.

**Supplementary Note 2. Adhesion Testing**

The adhesion strength was measured via the lap shear test which was performed to quantify the shear strength of the hydrogel adhesion. All tests were performed under a constant shear rate of 10 mm min^−1^, and the shear strength was estimated by dividing the maximal shear force by the adhesion area.

**Supplementary Note 3. ECG Testing**

For ECG monitoring demonstration, electrodes were directly placed on the skin surface under resting or sweating conditions and ECG signals were recorded by Brain SpikerBox (Ann Arbor). Recording of ECG signals was pre-approved by the ethics committee of Institutional Review Board (IRB) at Shenzhen Institute of Advanced Technology, Chinese Academy of Sciences (Reference number: SIAT-IRB-210815-H0574). All participants are co-authors of the manuscript and have provided consents for participation.

**Supplementary Note 4. In vitro HIF-1α Expression Analysis**

To evaluate the wound healing potential of the HAST system’s oxygen provision, we conducted *in vitro* experiments using HUVEC cells. The control group was cultured in basic medium, while experimental groups were subjected to hypoxic conditions (no oxygen provision for 6 hours under high glucose conditions of 450 mg/dL). One experimental group was co-cultured with sterilized spun membranes for oxygen provision. After 24 hours, cells were fixed with 4% paraformaldehyde (PFA) for 15 minutes. They were then permeabilized with 0.3 % Triton X-100 for 10 minutes, followed by blocking with 5% bovine serum albumin (BSA) for 30 minutes. To assess hypoxia, cells were incubated with HIF-1α primary antibody for 30 minutes at room temperature, followed by incubation with anti-rabbit immunoglobulin G (IgG) secondary antibody for 30 minutes at room temperature in the dark. After PBS washing, cell nuclei were stained with DAPI for 10 minutes. Imaging was performed using a confocal laser scanning microscope, and quantification was conducted using ImageJ software.

**Supplementary Note 5. Evaluation of Cell Proliferation and Viability**

Cell proliferation and viability were evaluated using a Calcein Acetoxymethyl Ester (calcein AM) cell live kit and 4’,6-diamidino-2-phenylindole dihydrochloride (DAPI, both from Beyotime Biotechnology). Samples were imaged using an FV1000 confocal laser scanning biological microscope (Olympus). Live cells were visualized in green (calcein-AM staining), while cell nuclei appeared blue (DAPI staining). To quantify cell proliferation and viability, a separate experiment was conducted. Cells were cultured in a 96-well plate using medium with or without the HAST system (n=3 for each group). After 24 hours of incubation, 10 μL of Cell Counting Kit-8 (CCK-8) solution was added to each well and incubated for 2 hours at 37°C, after which absorbance was measured at 450 nm using a microplate reader. The proliferation ratio was calculated using the following formula:

Proliferation ratio (%) = [(with HAST – without HAST) / (without HAST)] × 100%

**Supplementary Note 6. In vitro Cytocompatibility Studies**

The separated components of the HAST system were sterilized under 254 nm UV light. The sterilization process lasted for 30 minutes. A suspension of Human Umbilical Vein Endothelial Cells (HUVECs) was then added to each well of a 96-well plate at a density of 5000 cells per well. The separated components of the HAST system were then placed in cell-seeded 96-well plates. Cells were treated with appropriate media and incubated at 37°C with 5% CO_2_ for the duration of the study. After 24 hours of incubation, 10 μL of CCK-8 solution was added to each well, followed by a further 2-hour incubation at 37°C. Subsequently, absorbance was measured at 450 nm using a microplate reader.

**Supplementary Note 7. Blood Compatibility Test**

The blood compatibility of the individual components of the HAST system was evaluated using a hemolysis test. Fresh blood (8 mL) was diluted with 10 mL of 0.9% NaCl solution, while 0.2 g of each material was soaked in 5 mL of 0.9% NaCl solution at 37°C in a water bath for 30 minutes. Negative and positive control groups were prepared using 5.2 mL of 0.9% NaCl solution and 5.2 mL of distilled water, respectively. After 30 minutes, 2 mL of freshly diluted blood was slowly added to all centrifuge tubes and mixed gently. The mixtures were then incubated at 37°C in a water bath for 60 minutes. Following incubation, the solutions were transferred to new centrifuge tubes, centrifuged at 2500 rpm for 5 minutes, and the supernatant was collected for absorbance measurement at 545 nm. The hemolysis rate was calculated using the following formula:

Hemolysis Rate (%) = [(A - B) / (C - B)] × 100%

where A is the absorbance of the sample, B is the absorbance of the negative control, and C is the absorbance of the positive control.

**Supplementary Note 8. Reactive Oxygen Species (ROS) Immunofluorescence Staining**

Three groups of mice were selected and, according to the previously described method, 13 mm diameter circular wounds were created on their backs. Each group was treated as follows: one group received no patch treatment (Normal), one group was treated with PVP/H_2_O_2_/PVA electrospun patches (H_2_O_2_), and another group received both PDA/CAT/PVA and PVP/H_2_O_2_/PVA electrospun patches (CAT + H_2_O_2_), all of which were fixed using 3M tape. After 30 minutes, the mice were euthanized, and the wound tissues were extracted and immediately frozen in optimal cutting temperature (OCT) compound at -80°C.

The samples were then sectioned into 10 µm thick slices using a cryostat. Sections were mounted onto glass slides. For reactive oxygen species (ROS) detection, tissue sections were incubated with 10 µM 2’,7’-Dichlorofluorescin diacetate (DCFH-DA) for 30 minutes at 37°C in the dark. Green fluorescence, derived from DCFH-DA, indicated the presence of general ROS. For counterstaining, sections were treated with DAPI to visualize cell nuclei. Finally, sections were mounted and analyzed using confocal microscopy. ROS production was quantified by measuring fluorescence intensity using ImageJ software, comparing ROS levels across experimental groups.

**Supplementary Note 9. Immunofluorescence Staining**

For immunofluorescence analysis, tissue sections were serially sectioned at 5 μm, rinsed in PBS, and then blocked with 5% BSA solution for 1 hour. To evaluate cell proliferation and hypoxia, the slices were incubated with anti-Ki67 (Servicebio) at 4°C overnight, then washed several times with PBS. Nuclei were counterstained with DAPI. Sections were observed and photographed using a fluorescence microscope. Quantification of the staining was performed using ImageJ software.

**Supplementary Figures**





**Figure S1. Research landscape and innovation bridging adaptive sensing and hypoxic remediation.** Timeline showing independent evolution of adaptive sensing systems (left, blue: responding to light, pressure, sound, mechanical stimuli) and hypoxic-environment remediation technologies (right, purple: oxygen delivery via microalgae, microspheres, hydrogels, nanobubbles) from 2020–2025. Critical gap: No existing platform addresses biosensor adaptation to hypoxic microenvironments. Our work (bottom): First integrated system combining O_2_ generation (CAT/H_2_O_2_), multiplexed enzymatic biosensing, and electrical stimulation, enabling accurate monitoring and accelerated healing in oxygen-deficient wounds. Scale bar: 1 cm. Images reproduced with permission from references: ^[1]^Copyright 2021, Springer Nature; ^[2]^Copyright 2021, Springer Nature; ^[3]^Copyright 2022, Springer Nature; ^[4]^Copyright 2023, Springer Nature; ^[5]^Copyright 2024, Springer Nature; ^[6]^Copyright 2025, Springer Nature; ^[7]^Copyright 2020, The American Association for the Advancement of Science; ^[8]^Copyright 2021, The American Association for the Advancement of Science; ^[9]^Copyright 2023, The American Association for the Advancement of Science; ^[10]^Copyright 2023, John Wiley and Sons; ^[11]^Copyright 2024, Springer Nature; ^[12]^Copyright 2025, American Chemical Society;


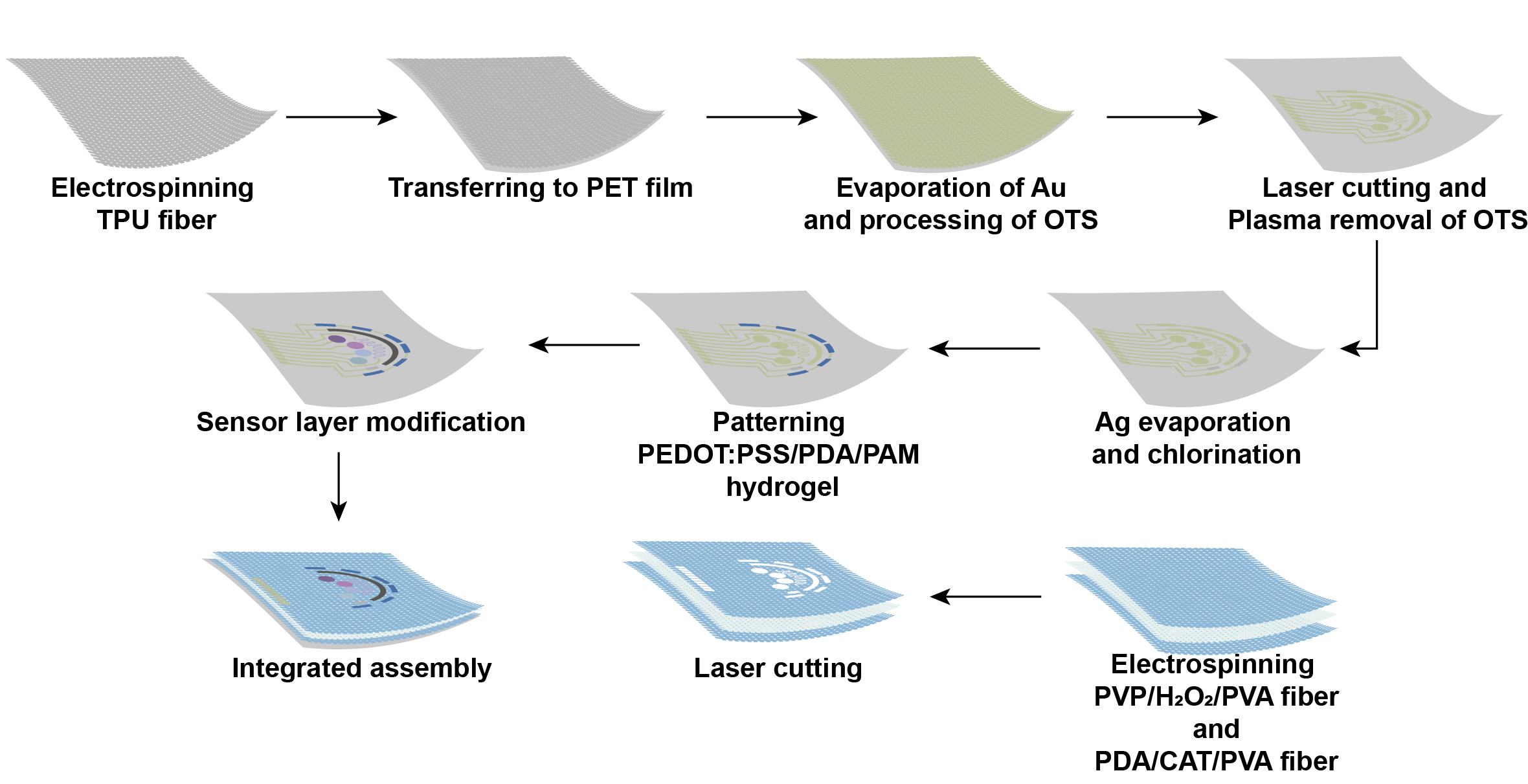


**Figure S2. Fabrication of Hypoxia-Adaptive Smart Theragnostic (HAST) system.** First, a TPU fiber was prepared via electrospinning and transferred onto a PET substrate. A 100 nm gold layer was deposited by thermal evaporation and treated with OTS. Next, UV laser cutting was used to pattern the electrode, and OTS was removed from the sensing area using plasma cleaning. A chlorinated Ag layer was fabricated to produce an Ag/AgCl reference electrode, and the patterned hydrogel electrode was transferred onto the gold electrode. Subsequently, multifunctional sensing films were modified onto the gold electrode surface as described earlier. Finally, the patterned PVP/H_2_O_2_/PVA and PDA/CAT/PVA electrospun fibers were assembled to create the integrated HAST system.


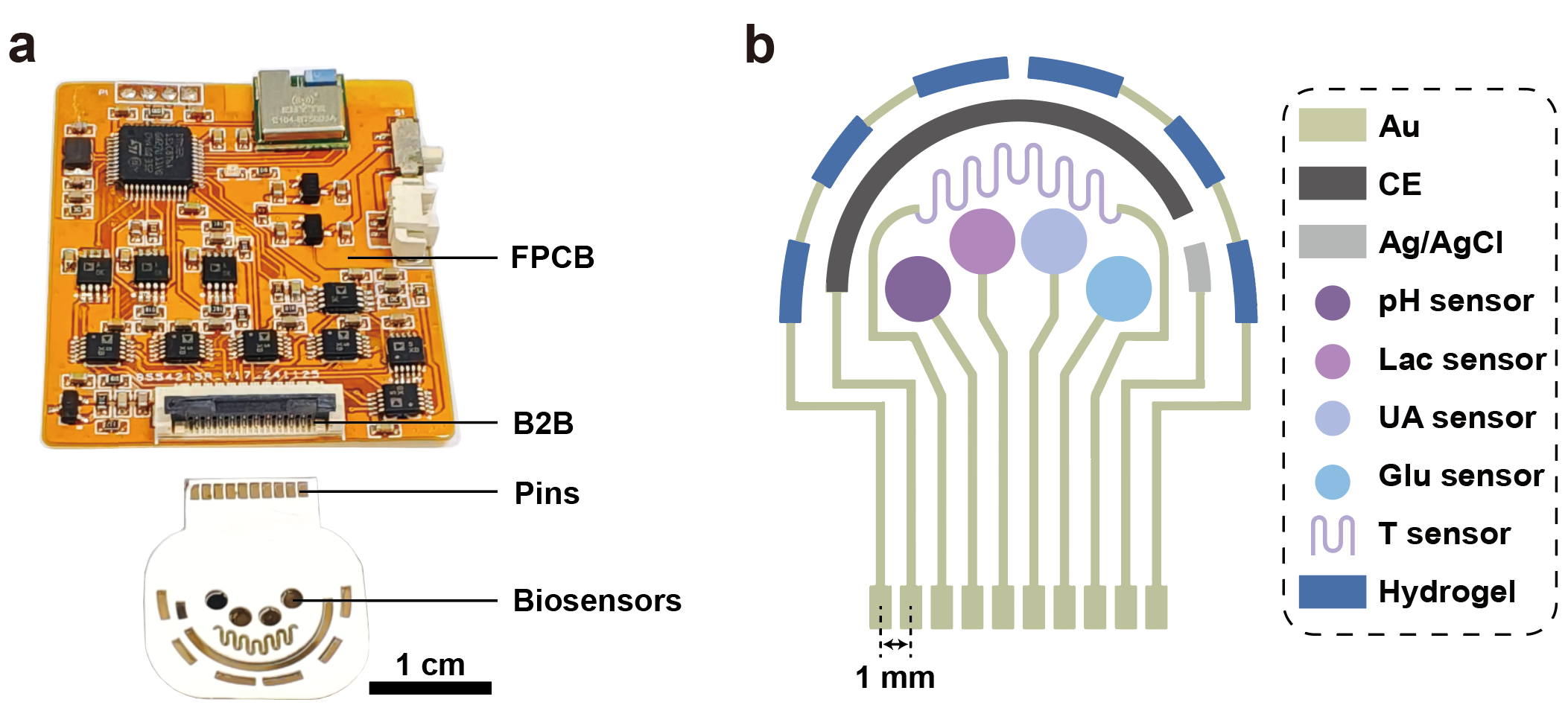


**Figure S3. Schematic illustration and photograph of the plug-in interface between the flexible biosensor array and the signal acquisition circuit.** a) Gold electrode traces from the biosensor extend to form connection pins precisely aligned with the female Board-to-Board (B2B) connector on the flexible printed circuit board (FPCB). The B2B connector interface can be configured with coplanar (as shown) or non-coplanar layouts depending on specific needs. The gold pins are mechanically clamped by the B2B socket, ensuring stable electrical contact and reliable signal transmission. This plug-in design allows easy biosensor replacement, preserves the reusable electronic module, and maintains robust mechanical integrity during *in vivo* operation. b) The top view of the HAST electrode array shows a 1-mm center-to-center spacing between each pair of contact pins.


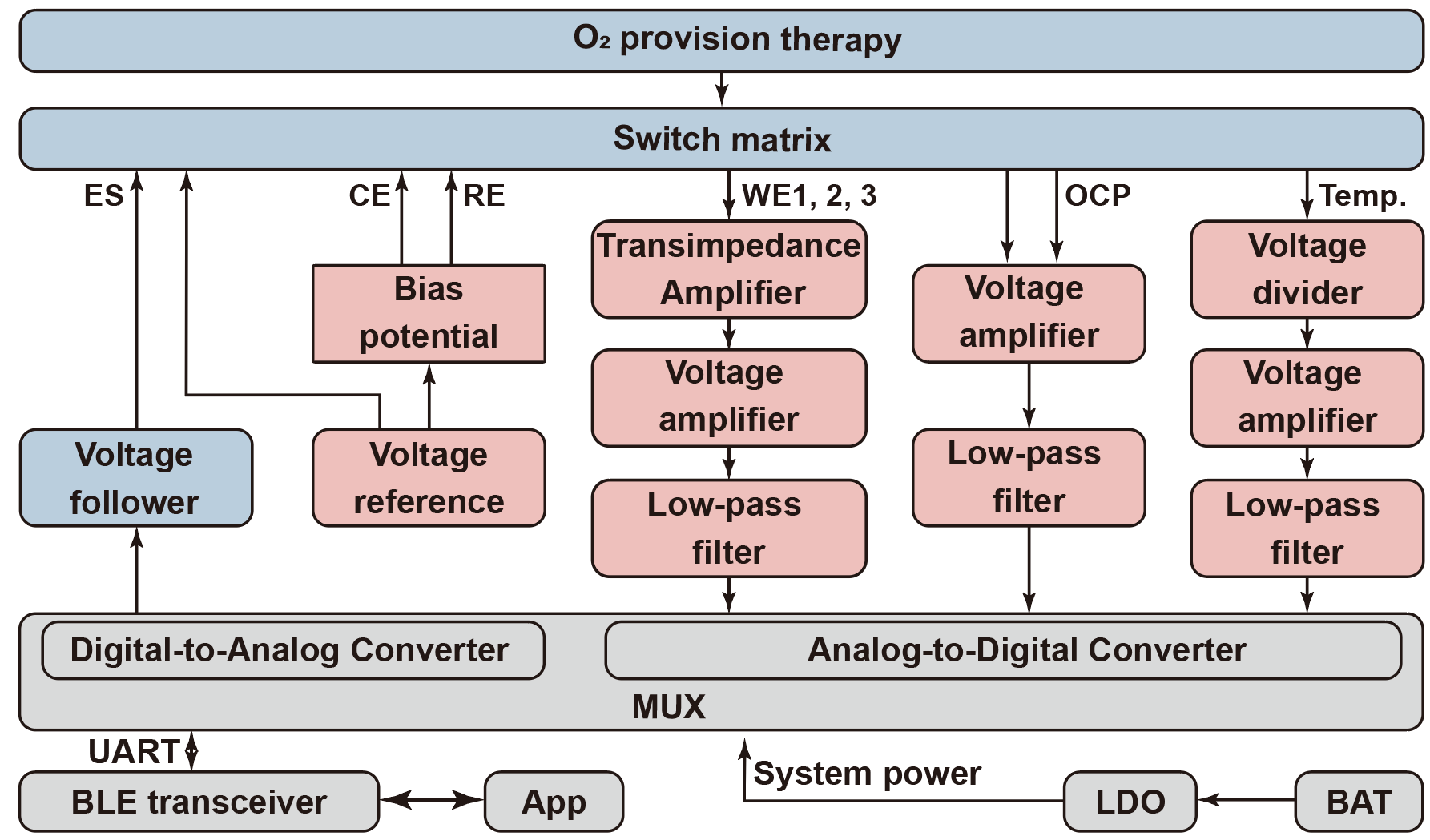


**Figure S4. Circuit diagram of the integrated wireless biochip** where the gray modules represent the underlying infrastructure (power management, Bluetooth transmission, and data processing), blue modules indicate the oxygen restoration and ES control unit, and red modules show the multimodal sensing interface for biosignal acquisition and conversion.


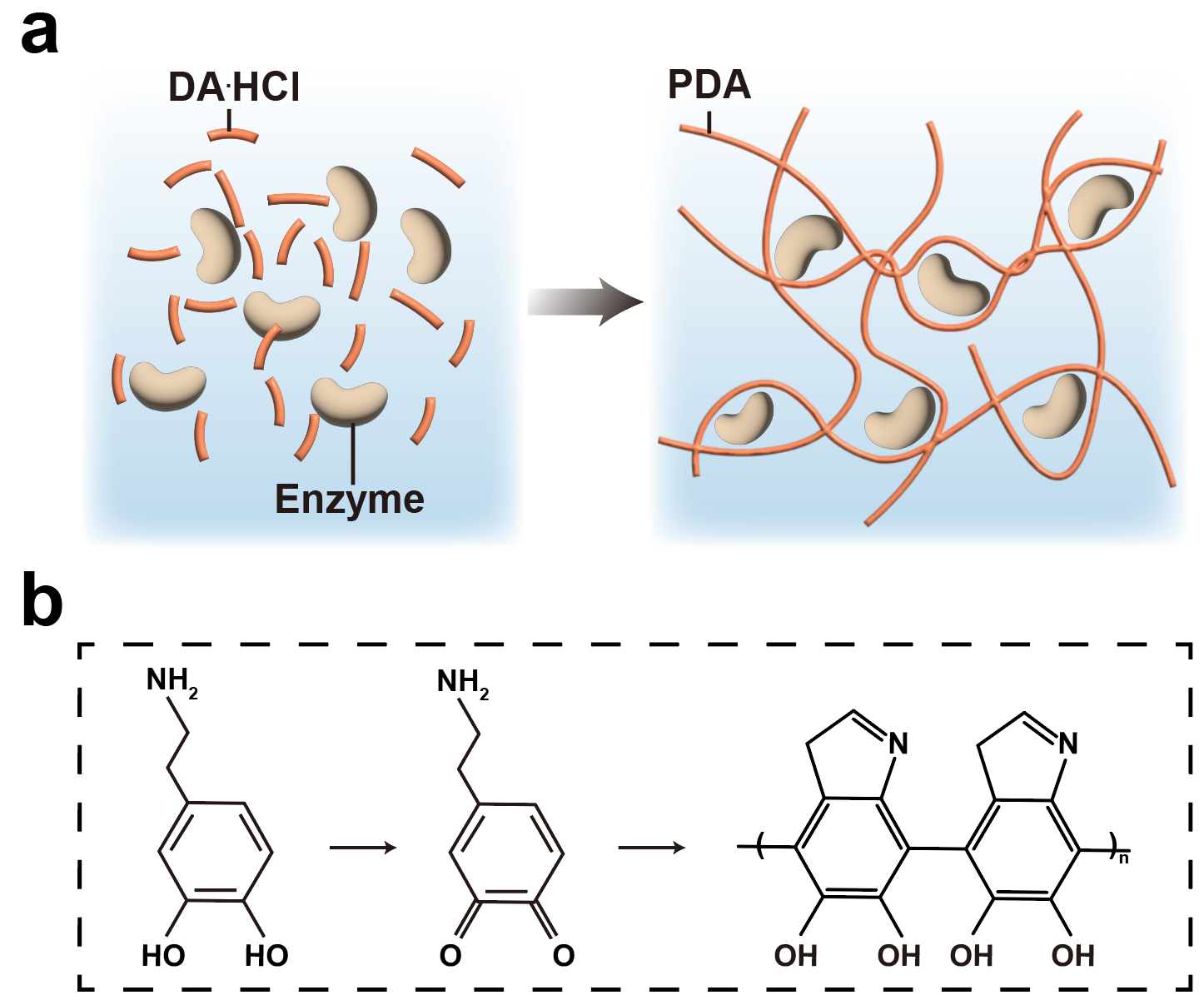


**Figure S5. Mechanism of *in situ* PDA-assisted enzyme immobilization.** (a) During dopamine polymerization, enzyme molecules are entrapped within the forming PDA network, resulting in enhanced enzyme loading, reduced enzyme leaching, and improved long-term stability. (b) Schematic representation of dopamine oxidation and self-polymerization into PDA, which provides abundant catechol and amine functionalities for enzyme immobilization and stabilization.


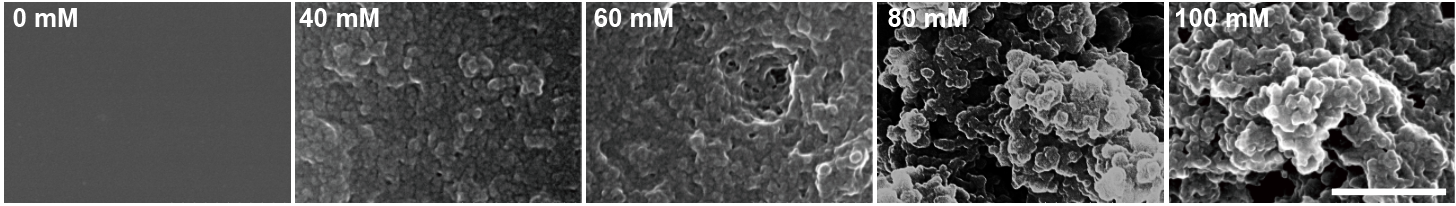


**Figure S6. SEM images of PEDOT:PSS/PDA/GOx with dopamine concentrations varied from 0 to 100 mM.** With increasing dopamine (DA) content, SEM images reveal a higher density of granular GOx, indicating that the amount of immobilized GOx can be effectively regulated by adjusting the DA concentration. Scale bar: 500 nm.


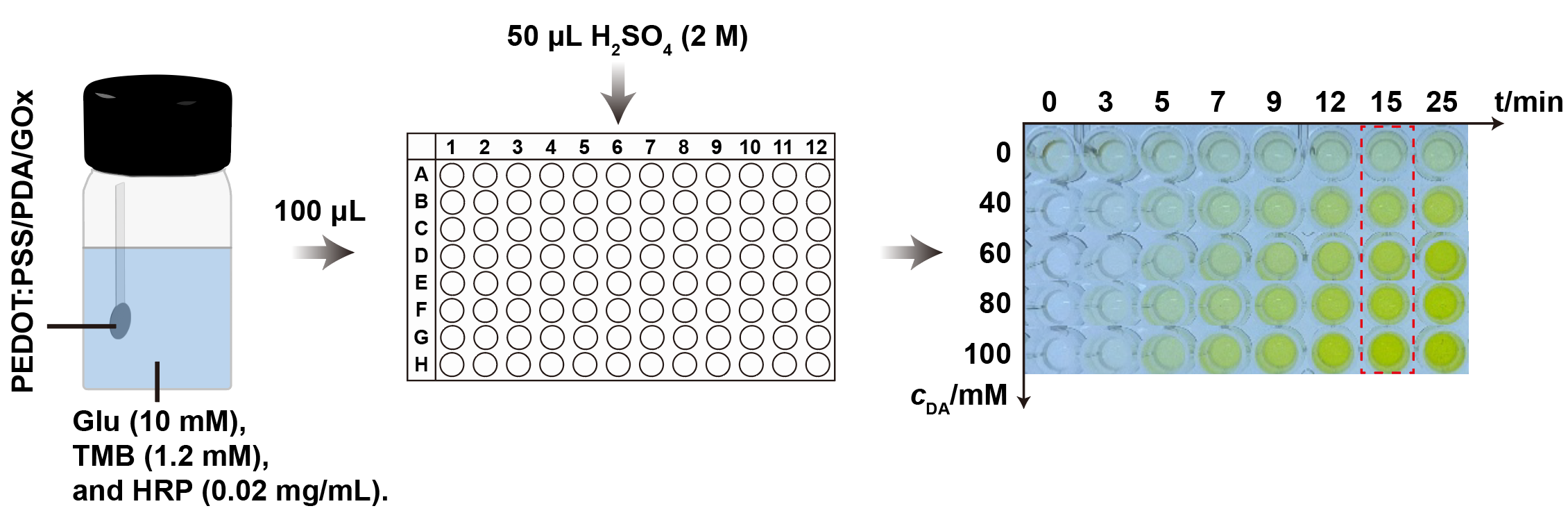


**Figure S7 Colorimetric assay for evaluating GOx activity in PEDOT:PSS/PDA/GOx biocomposites.** The electrode is incubated with glucose (10 mM), TMB (1.2 mM), and HRP (0.02 mg/mL). GOx-generated H_2_O_2_ oxidizes TMB to blue, which turns yellow upon H_2_SO_4_ (2 M) addition. Absorbance at 450 nm quantifies enzyme activity across different incubation times and glucose concentrations.


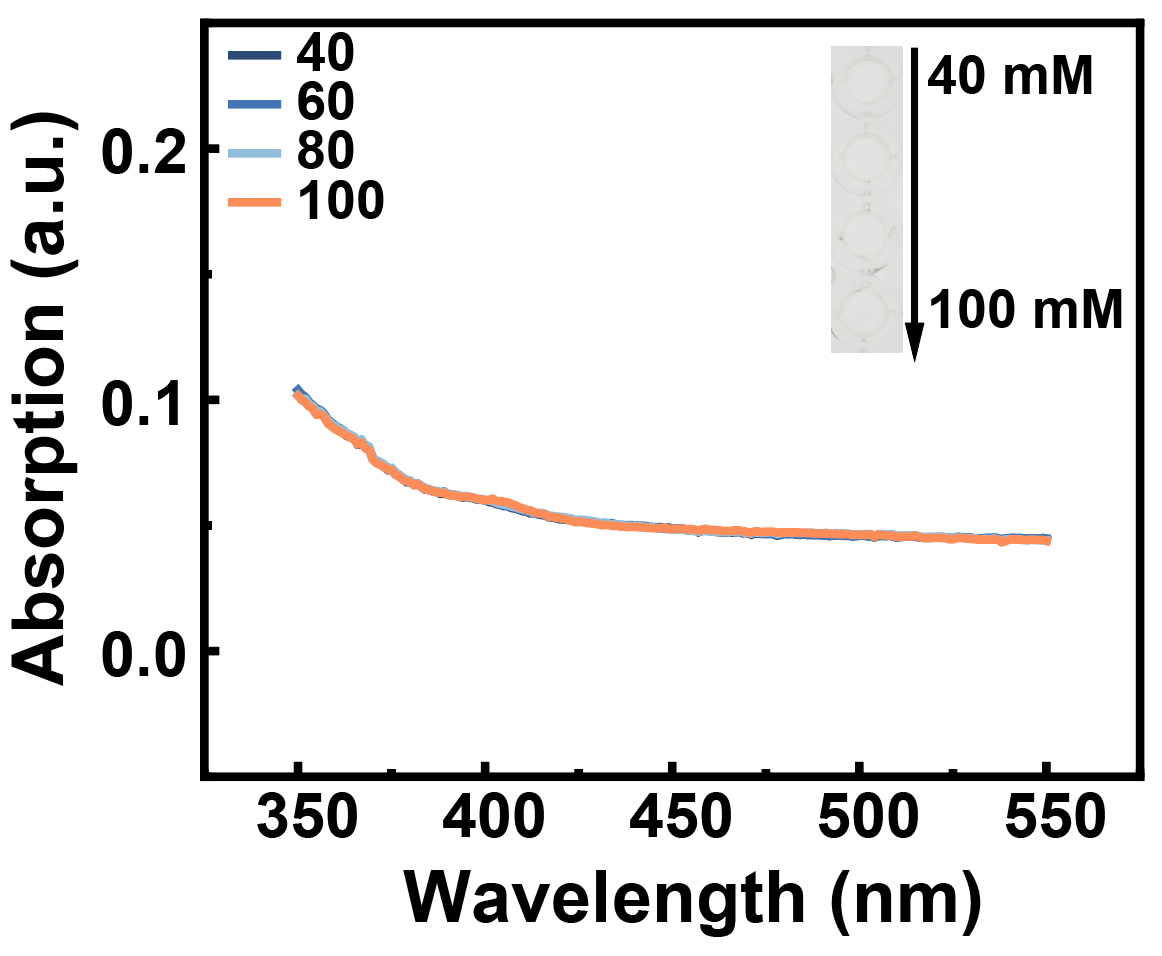


**Figure S8.** UV-vis absorption spectra of samples prepared with different initial dopamine (DA) feed concentrations (40-100 mM) for polydopamine (PDA) formation, followed by incubation in TMB solution for 15 min in the absence of glucose oxidase (GOx). Here, DA refers to the precursor concentration prior to polymerization into PDA, which serves as the enzyme immobilization matrix after purification (centrifugation and redispersion). No significant differences in absorption intensity or spectral features are observed across samples, indicating that PDA derived from varying DA feed does not induce colorimetric response in the absence of enzyme. Insets: corresponding optical images showing negligible color change.


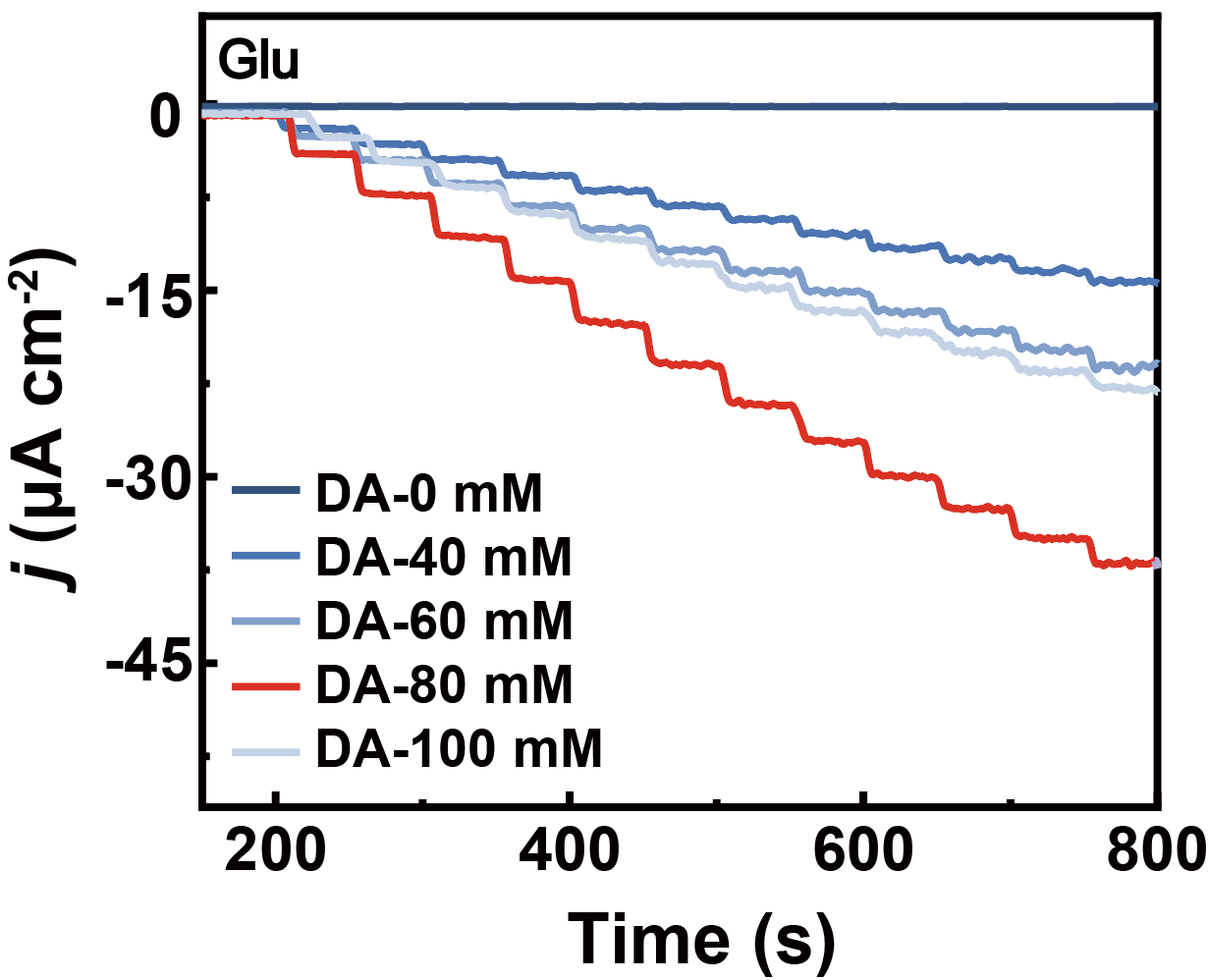


**Figure S9.** *i-t* curves of PEDOT:PSS/PDA/GOx with dopamine concentrations varied from 0 to 100 mM.


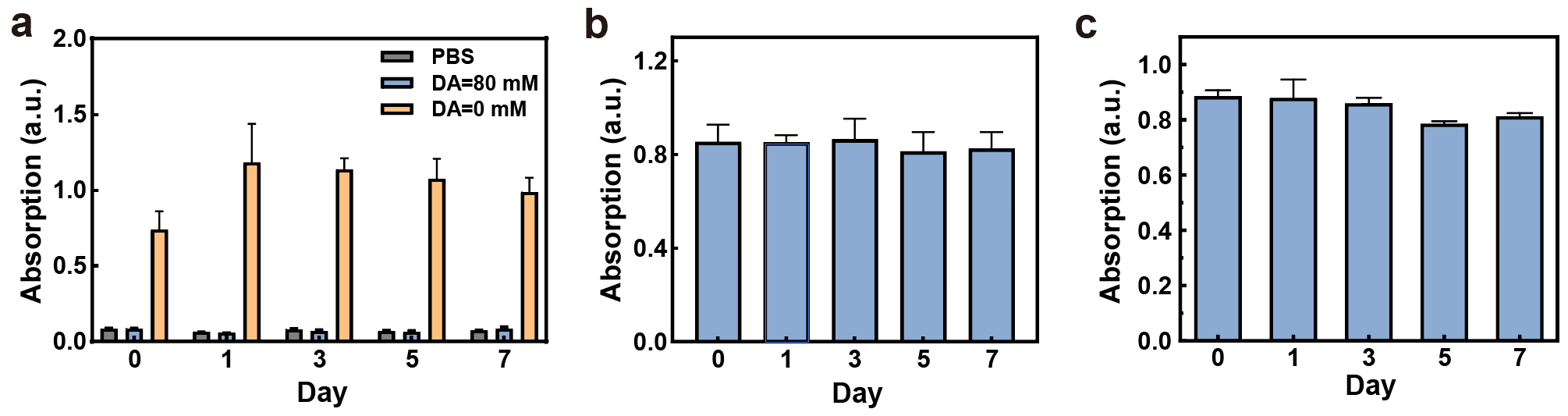


**Figure S10.** **Enzyme retention and storage stability of PDA-based biosensors.** a) GOx leaching assessment in solution at 37^o^C over 7 days by TMB assay. PDA-modified electrodes (DA=80 mM, blue) showed negligible enzyme release (<0.1 a.u.), while unmodified electrodes (DA=0 mM, orange) exhibited obvious leaching (~0.8-1.2 a.u.). PBS control (black) shows baseline. (b) Storage stability at 4^o^C. GOx activity on PDA-modified electrodes remained stable (~0.85 a.u.) over 7 days with <10% variation, enabling advance preparation and refrigerated storage. (c) Stability evaluation of the enzyme-functionalized sensing layer at 37^o^C over 7 days. The absorbance signal remains largely stable (~0.88 a.u.) over 7 days with <12% variation throughout the testing period, indicating good preservation of enzymatic activity under physiological working conditions. Data are presented as mean ± SD (n = 3).


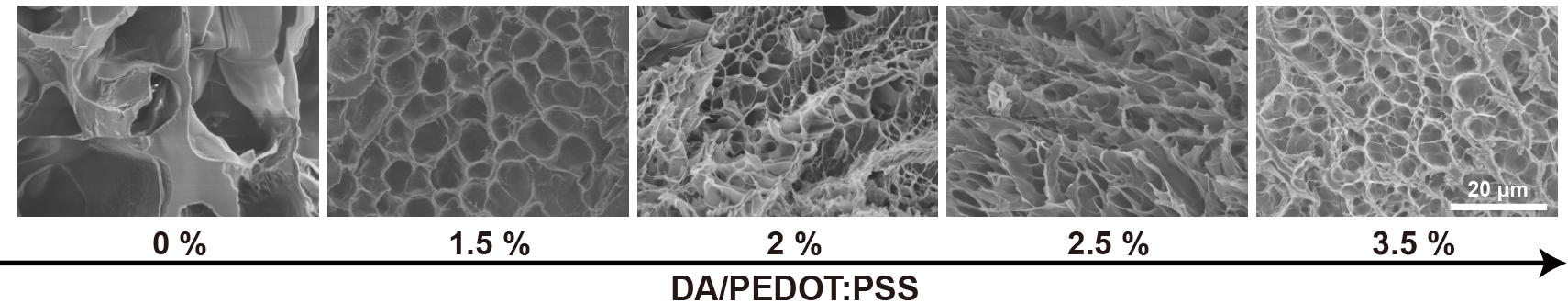


**Figure S11. SEM images of PEDOT:PSS/PDA/PAM hydrogels.** As the doping ratio increases, the pore structure becomes more compact, indicating the formation of more PDA chain segments that integrate with PAM to establish a double-network structure.


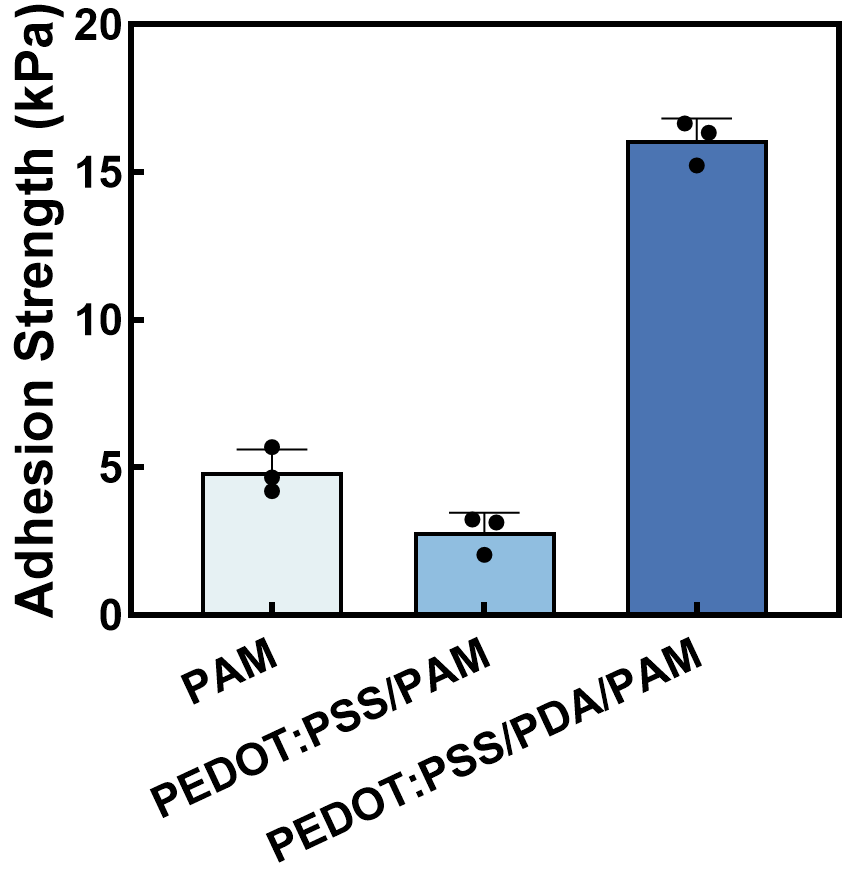


**Figure S12.** Lap shear measurements of adhesion strength for PAM, PEDOT:PSS/PAM, PEDOT:PSS/PDA/PAM (prepared with 2.4 mM DA under identical composition conditions). Data are presented as mean ± SD (n = 3).


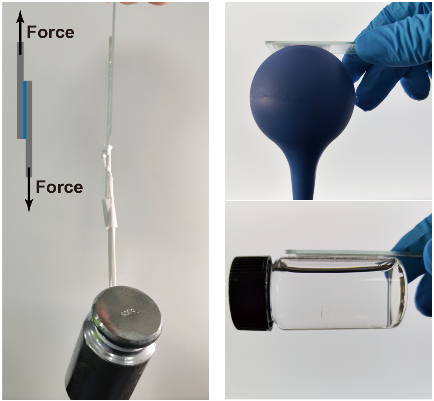


**Figure S13. Adhesive Properties of the PEDOT:PSS/PDA/PAM Hydrogel.** The images showcase the hydrogel’s strong adhesion to a range of materials, highlighting its versatile and robust adhesive properties.


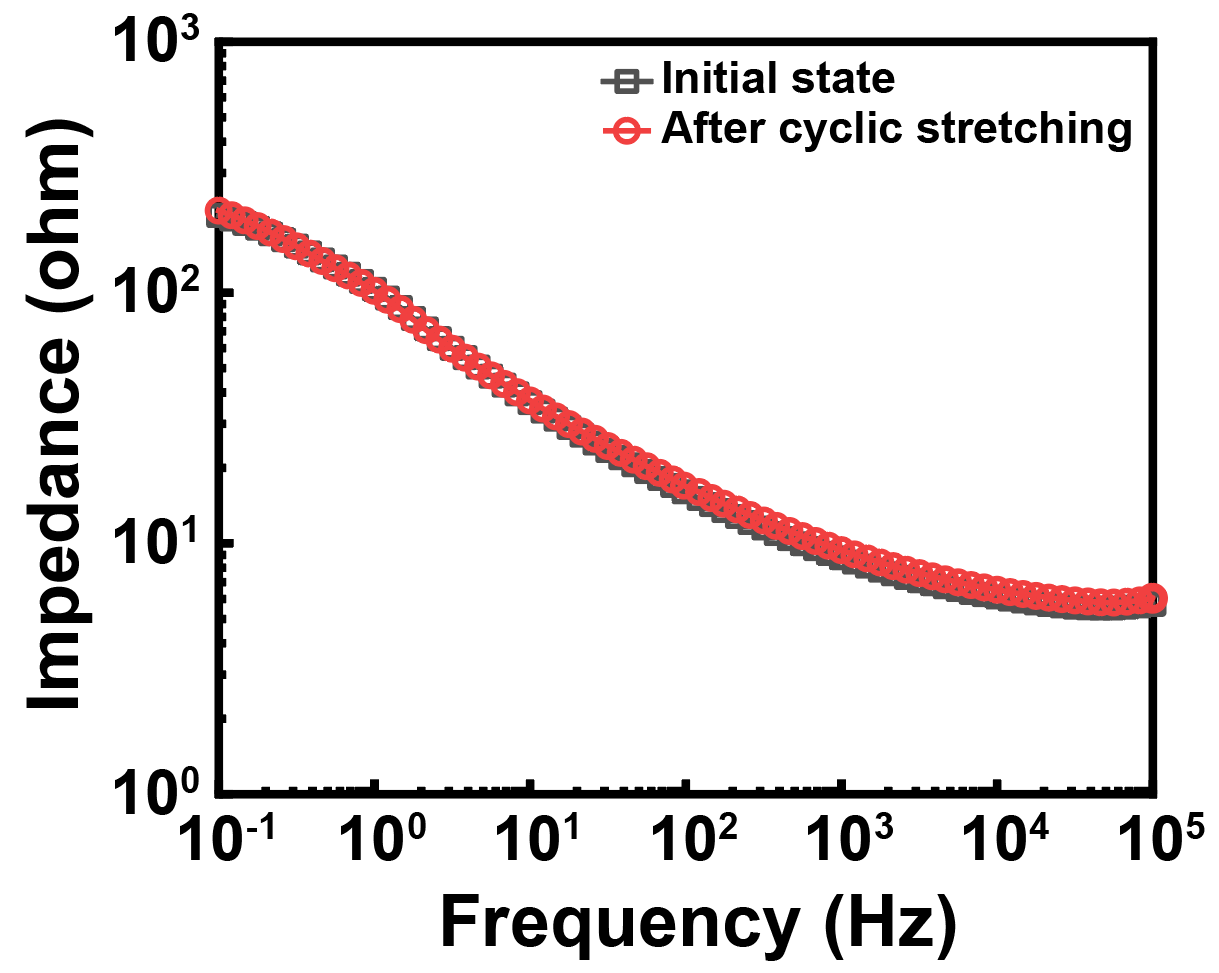


**Figure S14. Electrochemical impedance spectroscopy (EIS) characterization of the electrode before and after 1000 cycles of stretching.** The nearly overlapping impedance spectra indicate excellent electrical stability and preserved conductive pathways under repeated mechanical deformation.


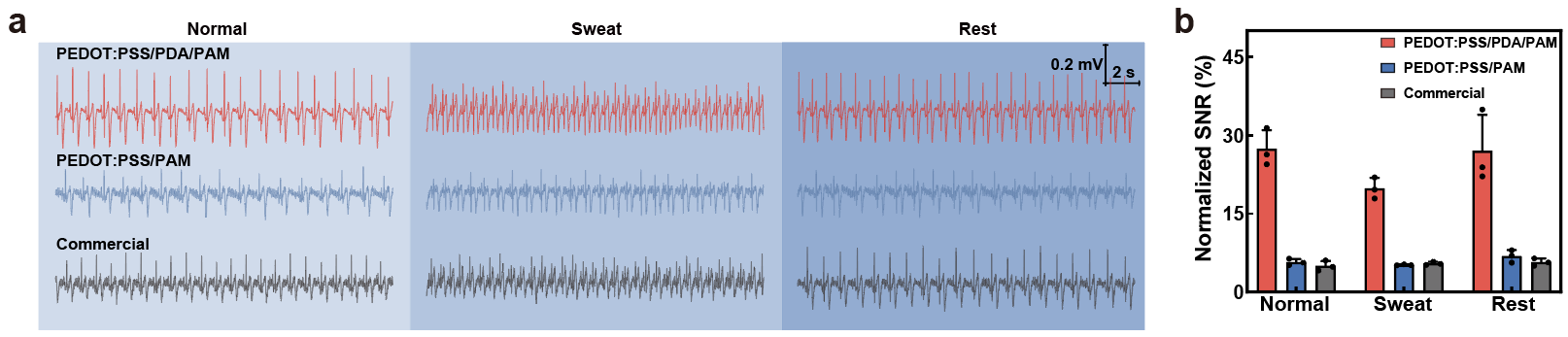


**Figure S15. Application of PEDOT Hydrogel in ECG Monitoring.** (a) Comparison of ECG tests between PEDOT:PSS/PDA/PAM, PEDOT:PSS/PAM, and commercial hydrogel and commercial hydrogel in three scenarios: 1) Before exercise (Normal), 2) During exercise-induced sweating (Sweat), 3) After 30 minutes of rest post-exercise (Rest). (b) Normalized Signal-to-Noise Ratio (SNR) comparison between PEDOT:PSS/PDA/PAM, PEDOT:PSS/PAM, and commercial hydrogel electrodes for ECG monitoring in three scenarios: Normal, Sweat and Rest. Data are presented as mean ± SD (n = 3).

**
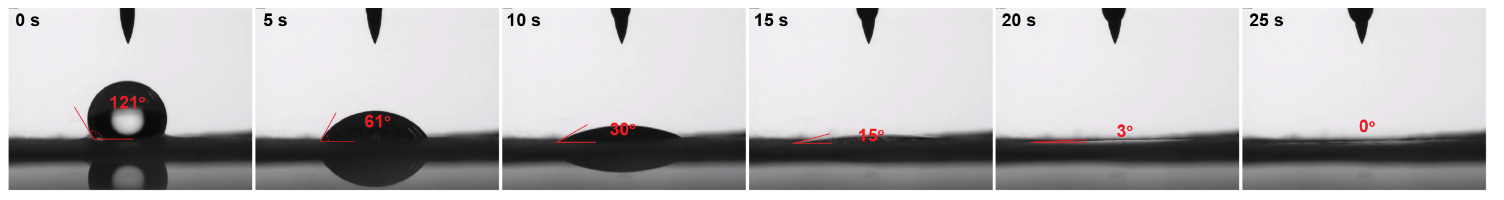
**

**Figure S16. Contact Angle Characterization.** Contact angle measurement of PVA fibers shows that PVA exhibits good hydrophilicity.


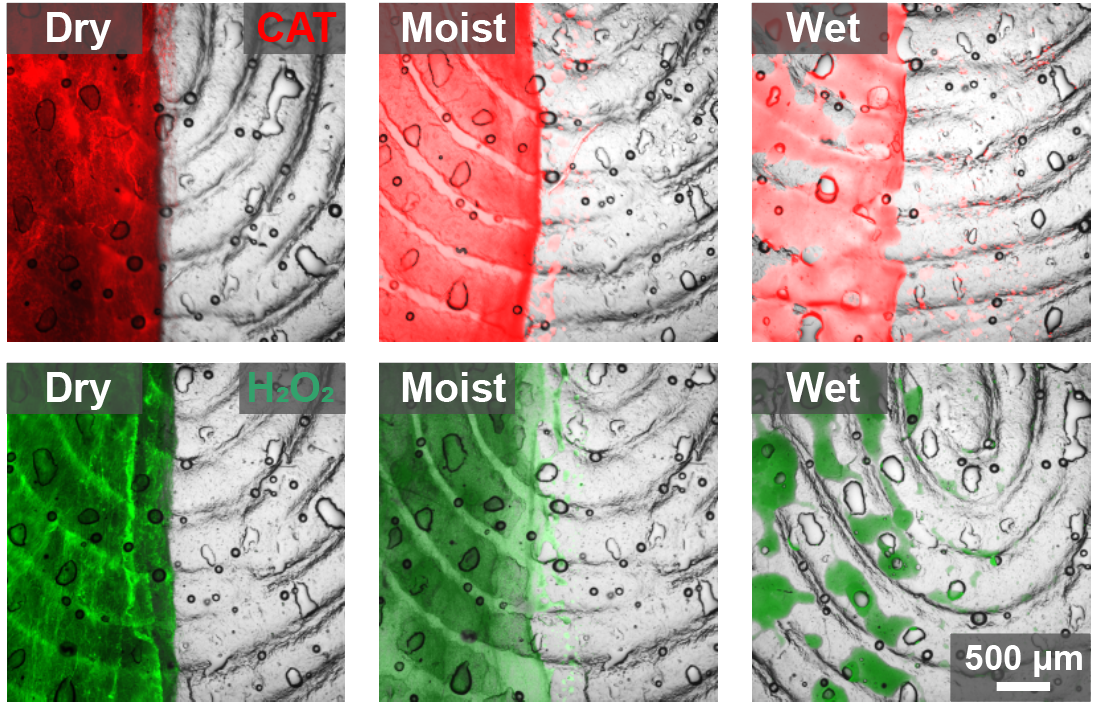


**Figure S17. Confocal fluorescence images of the oxygen-producing electrospun fibers**, with the top layer being CAT fibers labeled with red fluorescence and the bottom layer being PVP/H_2_O_2_ fibers labeled with green fluorescence. The dissolution process under dry, moist, and wet states is shown, where the fibers gradually dissolve and diffuse as the solution increases.


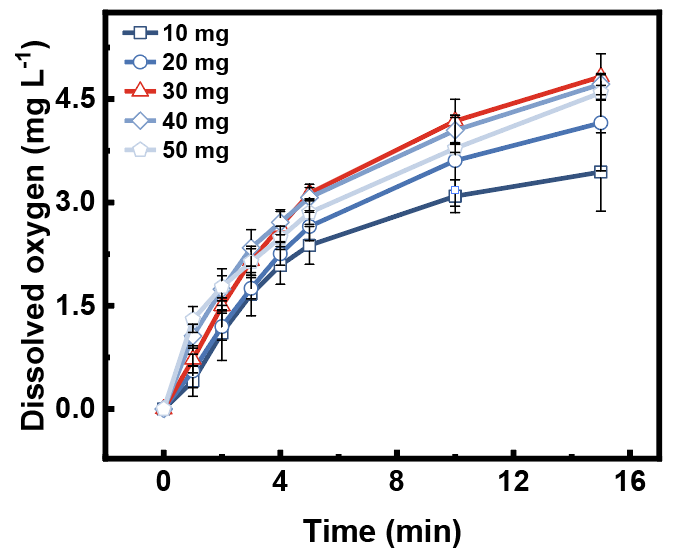


**Figure S18.** **Characterization of O_2_ release from O_2_ providing electrospun fibers.** When the amount of PVP/H_2_O_2_/PVA was fixed at 10 mg, the oxygen generation rate was optimized for different amounts of PDA/CAT/PVA. When the amount added was 30 mg or more, the oxygen release rates were similar. Data are presented as mean ± SD (n = 3).


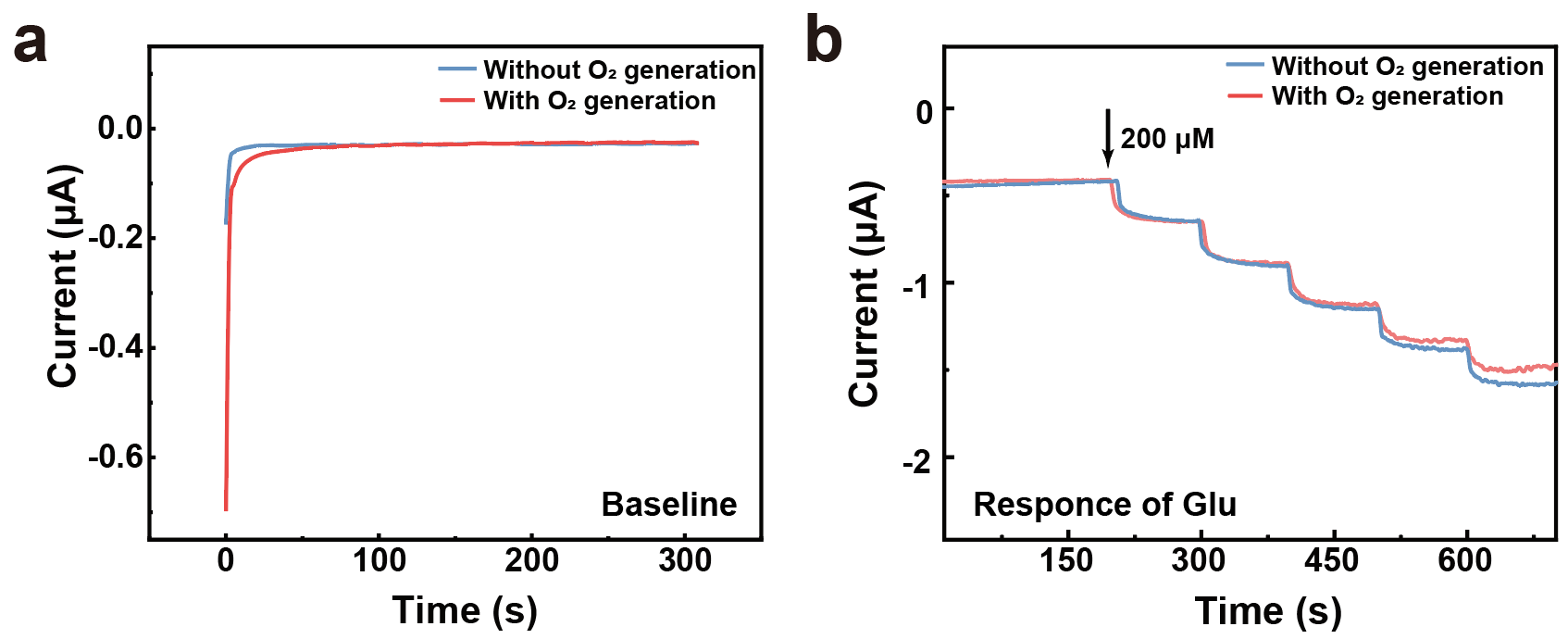


**Figure S19. Validation of oxygen generation system’s non-interference with biosensor performance.** (a) Amperometric baseline stability (*i-t* curve) of the glucose sensor without and with oxygen generation, demonstrating consistent baseline current over 300 seconds. (b) Stepwise amperometric responses to successive glucose additions (200 μM per step), showing comparable sensitivities without and with oxygen generation. These results confirm that the excess catalase effectively decomposes H_2_O_2_, thereby eliminating potential interference with the glucose biosensor. All measurements were conducted using PET/Au electrodes.


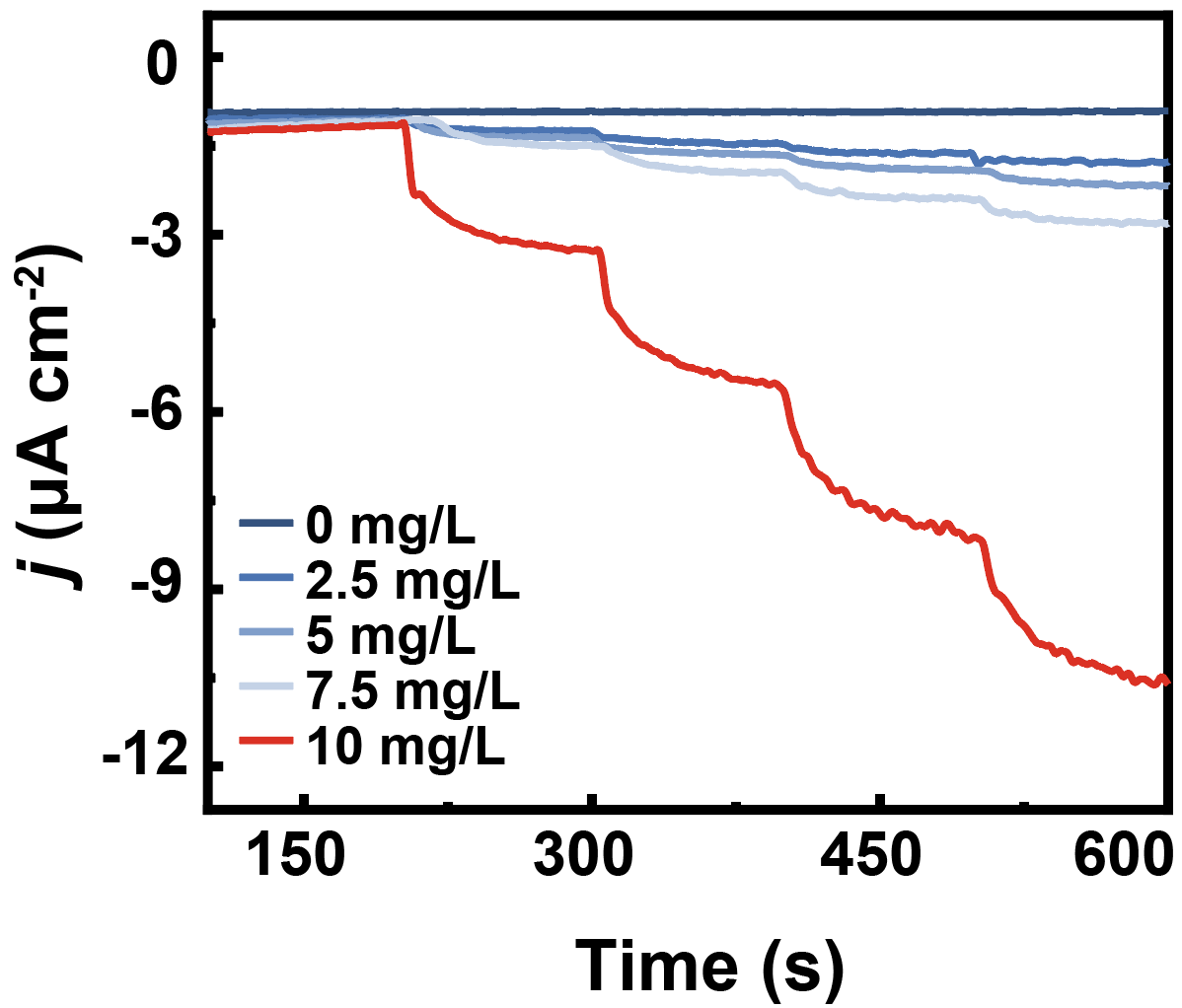


**Figure S20.** *i-t* curves of PEDOT:PSS/PDA/GOx biosensor for glucose detection under varying dissolved oxygen concentrations from 0 to 10 mg/L.


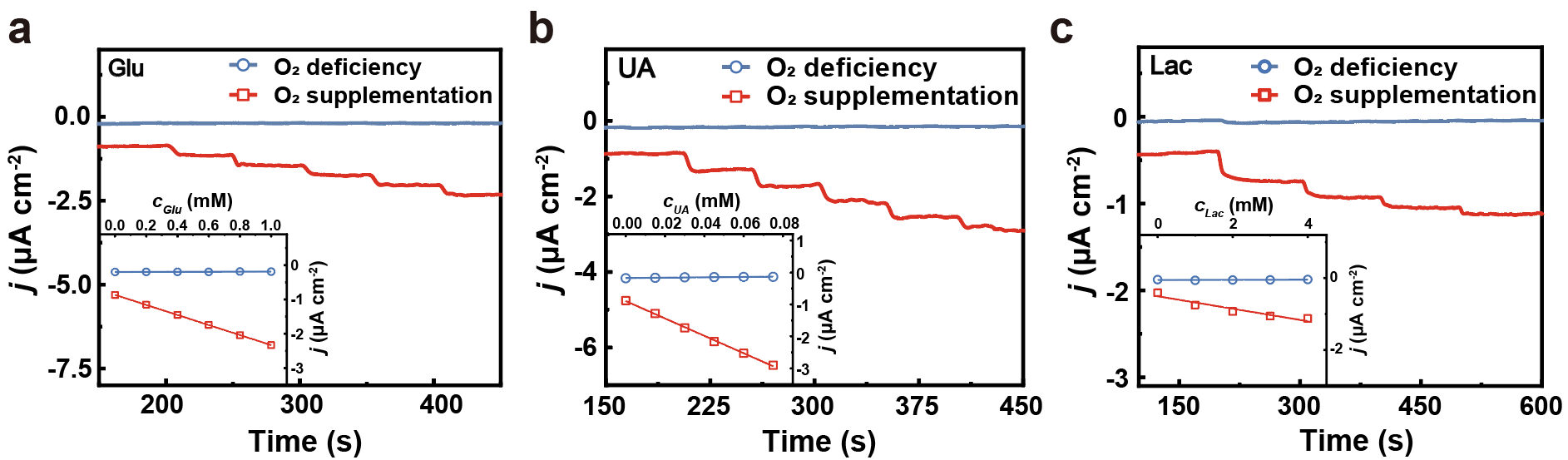


**Figure S21.** Comparison of *i-t* curves and their corresponding linear fits for Glu, UA, and Lac sensing under simulated hypoxic and O_2_-provision by HAST biochip. Insets show the corresponding linear fitting curves.


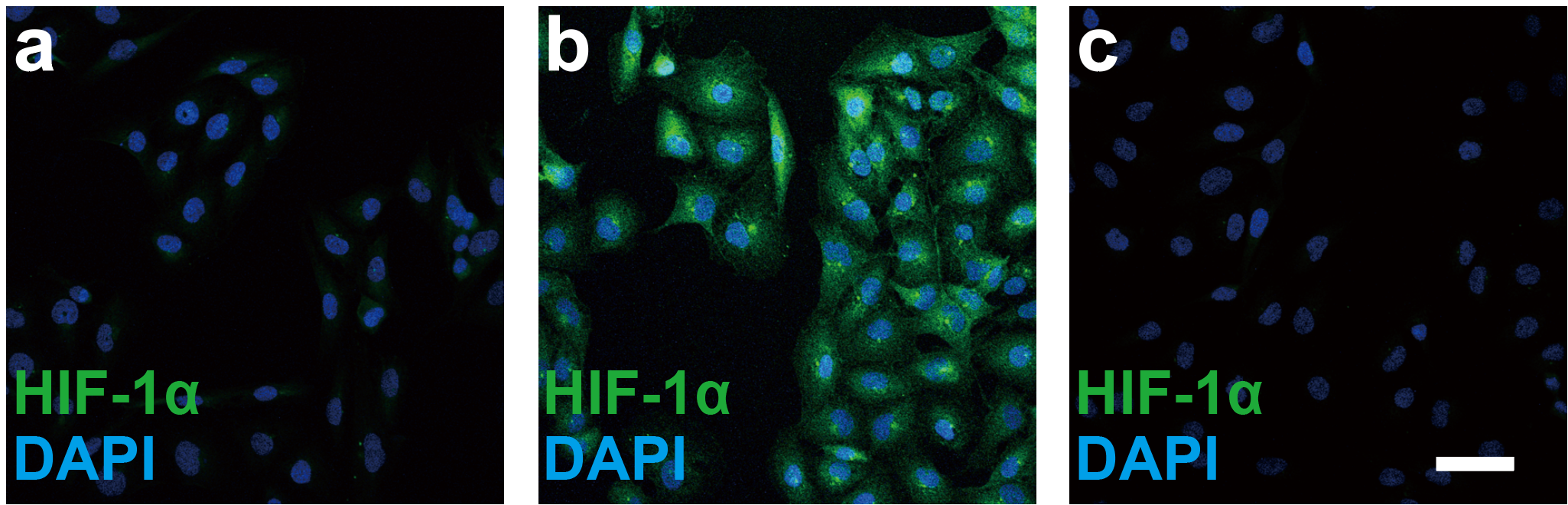


**Figure S22.** ***In vitro* validation of HIF-1α expression in HUVEC cells** under different conditions, including normal (a), hyperglycemia and hypoxia (HG + Hy) (b), and oxygen provision (c). Scale bar: 50 μm.


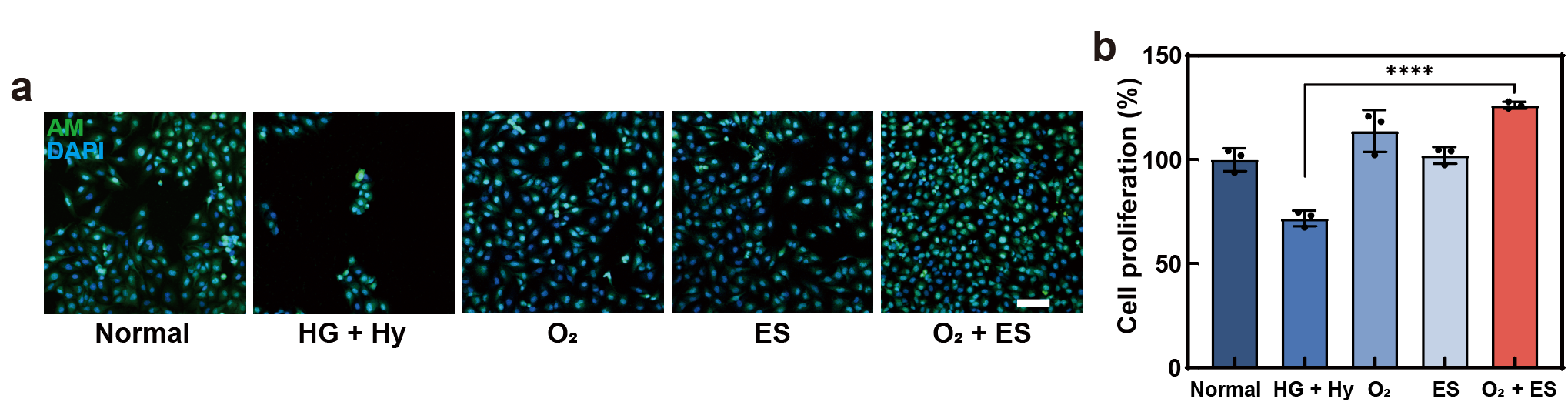


**Figure S23.** **Effects of oxygen provision and electrical stimulation on HUVEC proliferation under various conditions.** (a) HUVEC cell proliferation under different conditions: Normoxia (Normal), hyperglycemia and hypoxia (HG + Hy), and HG + Hy with O_2_ provision (O_2_), electrical stimulation (ES), or combined O_2_ + ES treatment (Green: AM, Blue: DAPI, scale bar: 100 μM). (b) Quantitative analysis of HUVEC cell proliferation rates under the conditions described in (a). Data are presented as mean ± SD (n = 3). Statistical significance was determined using one-way ANOVA followed by Dunnett's multiple-comparisons test. **P < 0.01; ***P < 0.001; ****P < 0.0001.


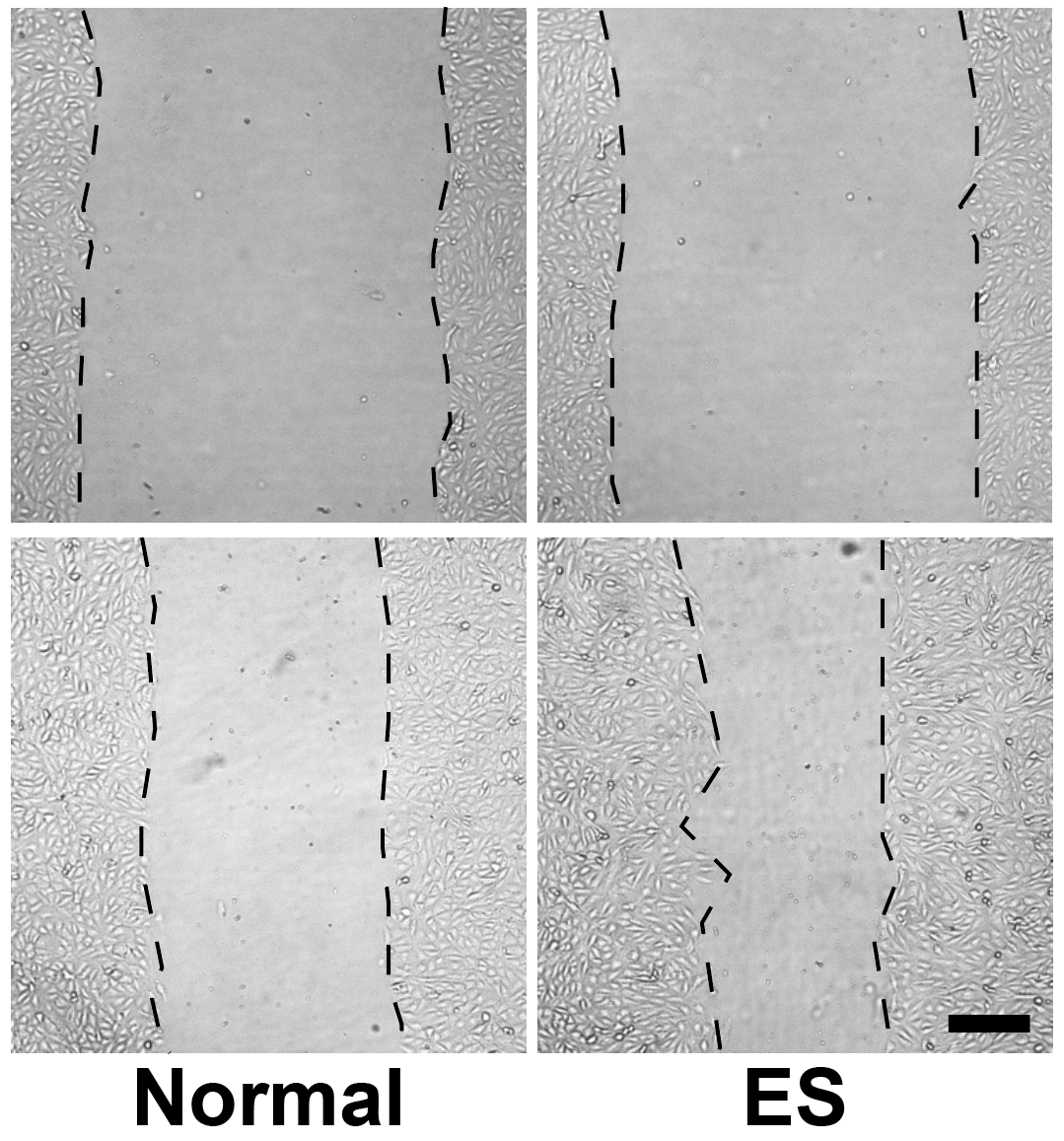


**Figure S24.** **HUVECs migration assessed by cell scratch assay under various conditions.** Representative images of cell scratch assays at 0 h (top panel) and 24 h (bottom panel) demonstrating HUVEC migration under different conditions Normal, and ES. Scale bar: 200 μm.


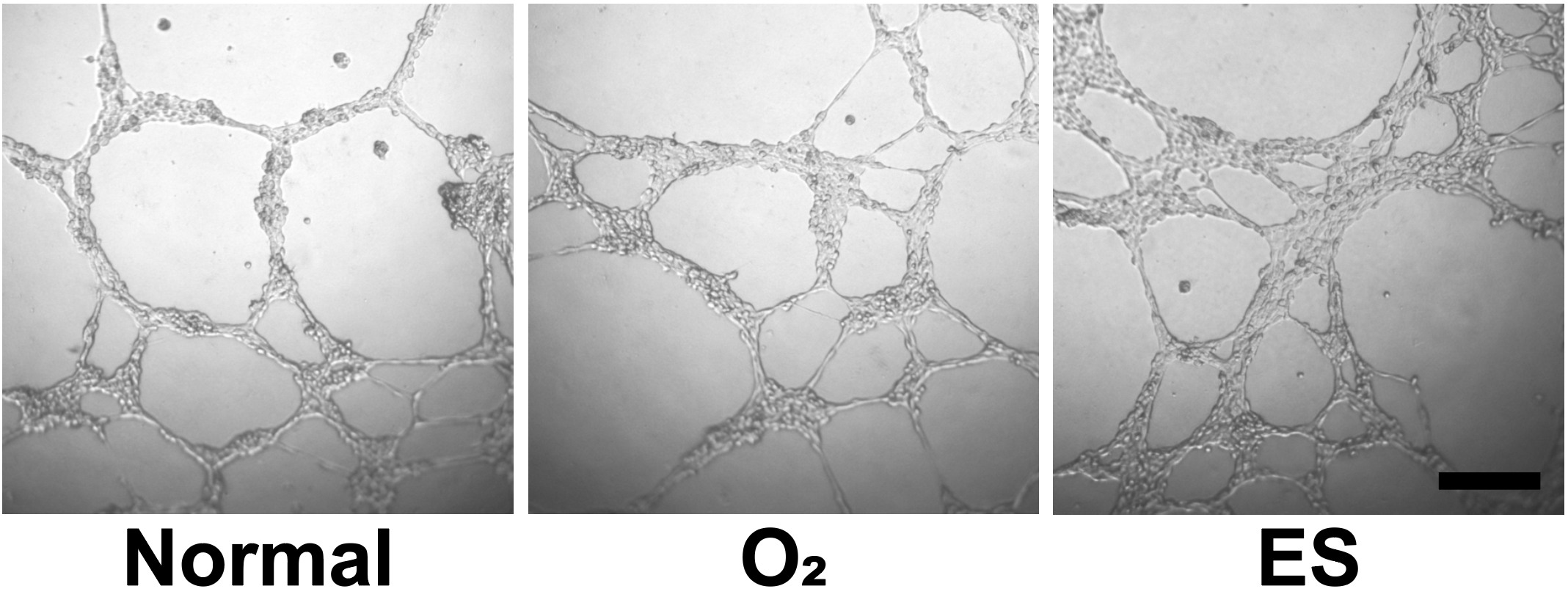

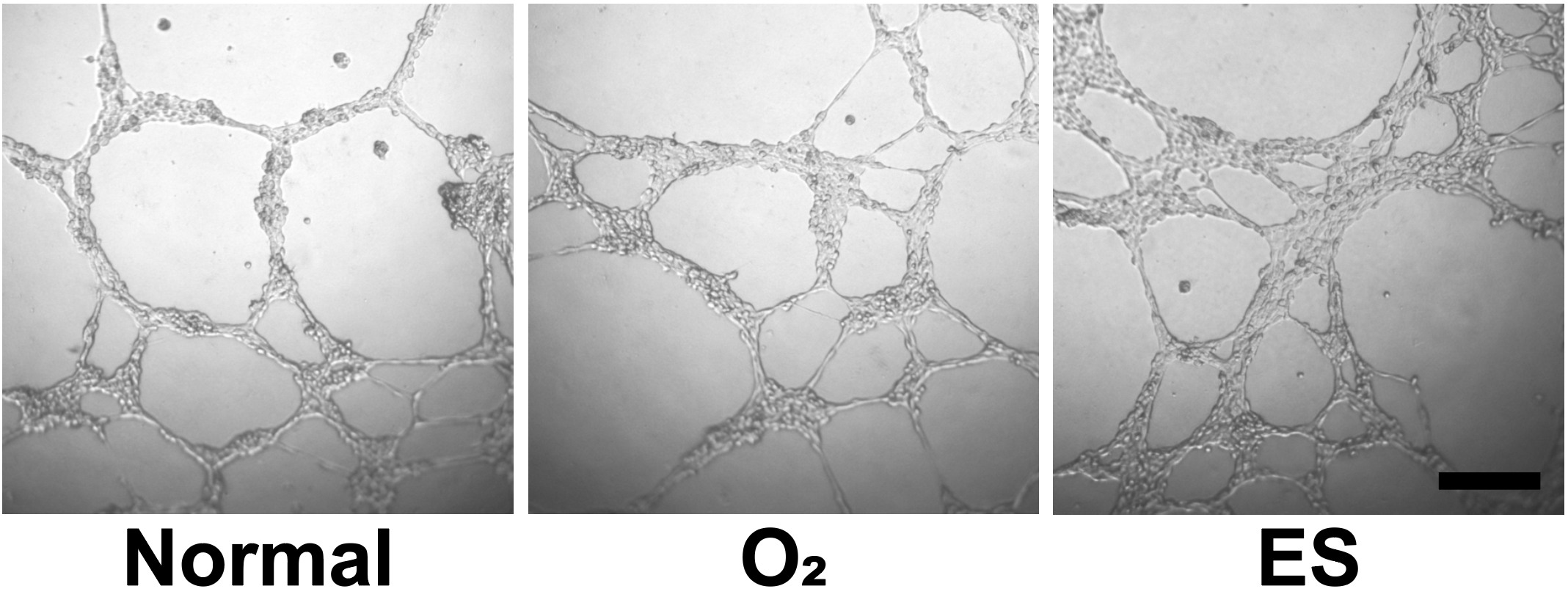


**Figure S25.** **Representative bright field images of HUVECs’ tube formation** after 6 h under different conditions: Normal, O_2_ and ES. Scale bar: 200 μm.


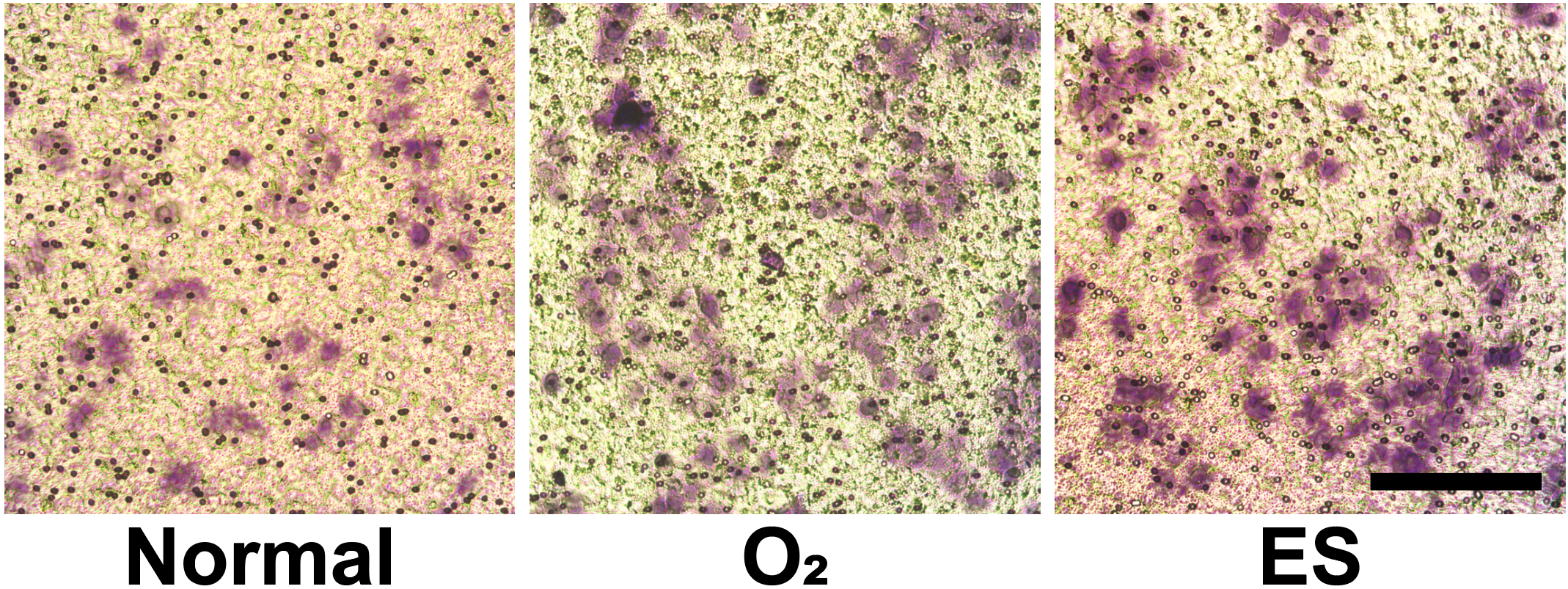

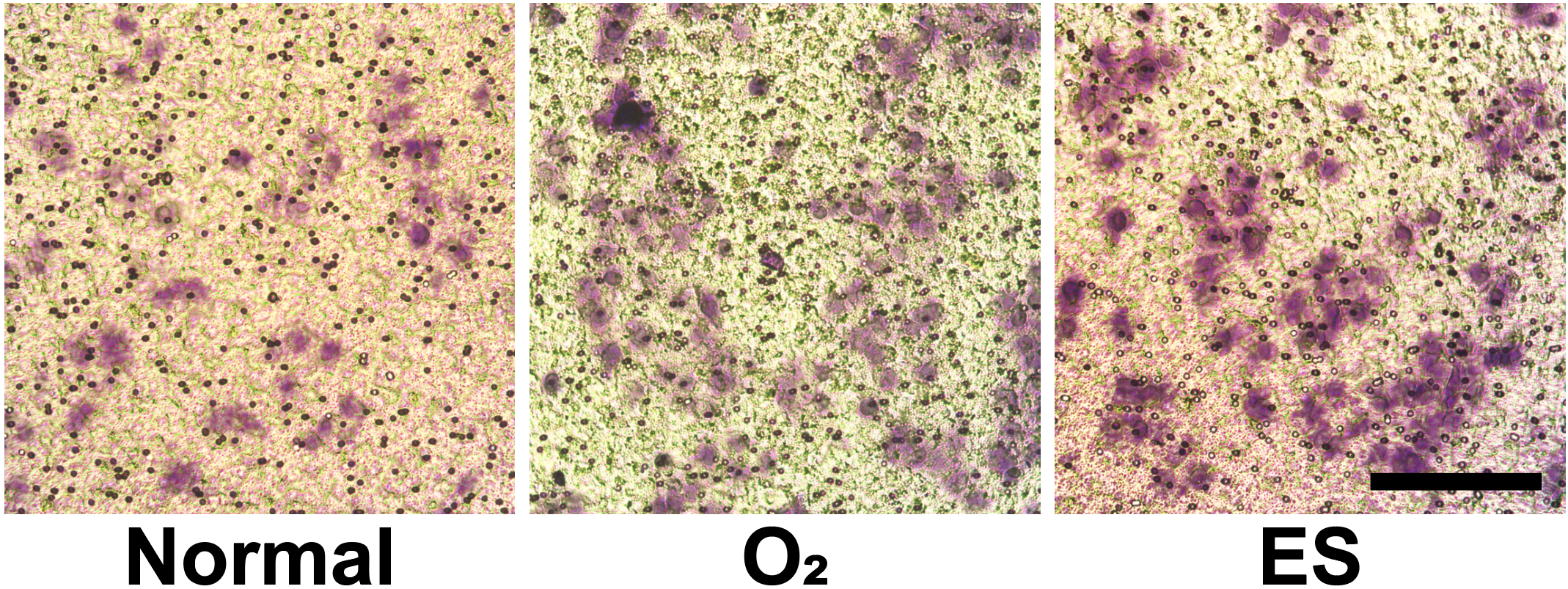


**Figure S26.** **Representative images of transwell assay** demonstrating HUVEC chemotaxis after 24 h under different conditions: Normal, O_2_ and ES. Scale bar: 200 μm.


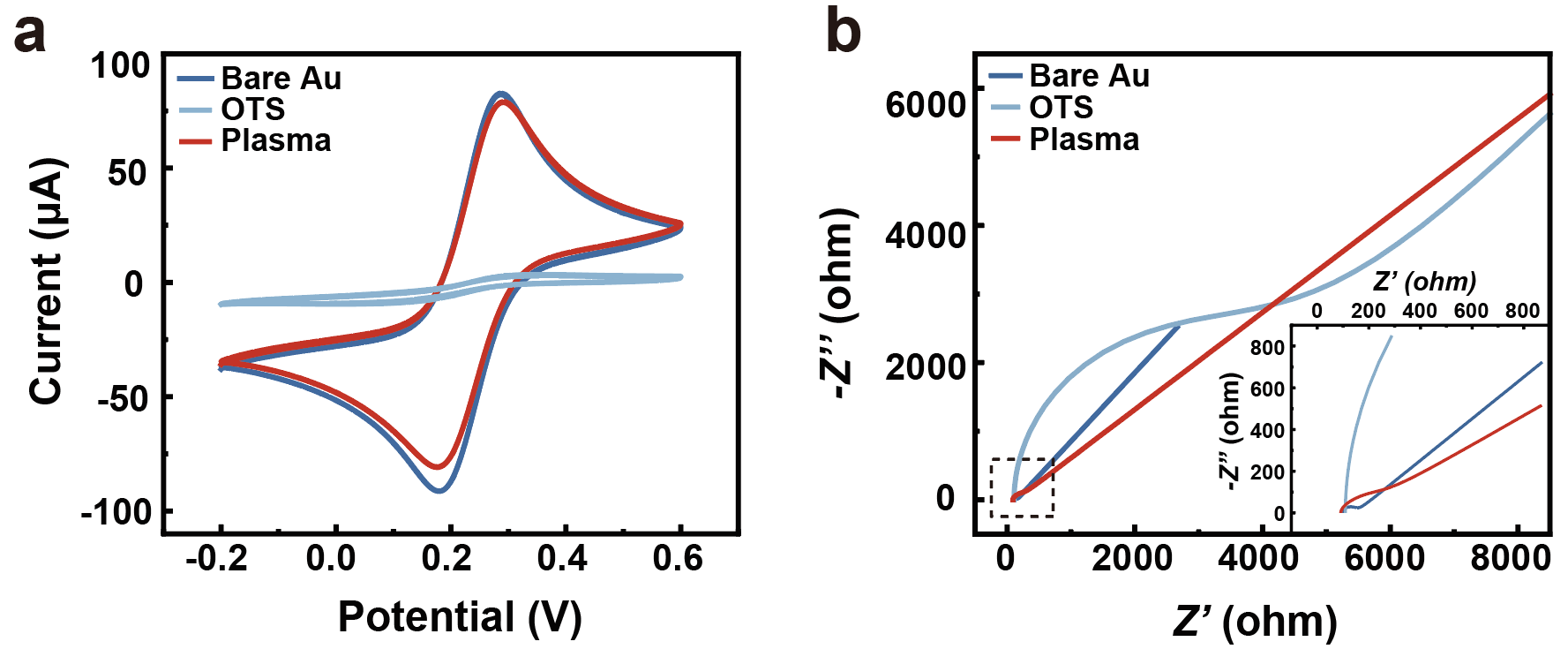


**Figure S27.** **Electrochemical characterization of electrode surface modifications.** (a) Cyclic voltammetry (CV) curves and (b) Nyquist plots of electrochemical impedance spectroscopy (EIS) of bare Au electrodes, after OTS modification, and following plasma treatment.


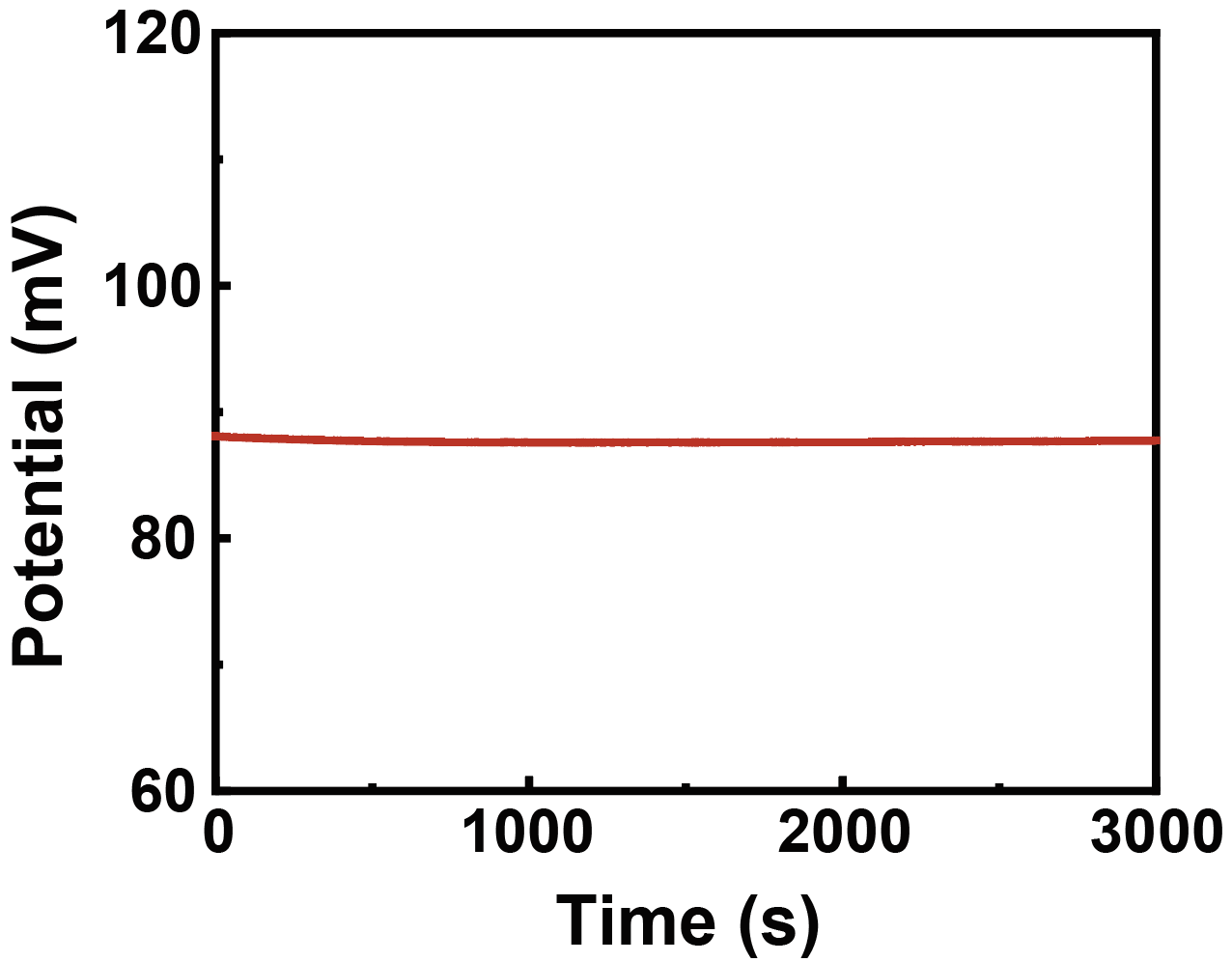


**Figure S28.** **Long-term potential stability of fabricated Ag/AgCl electrode** based on open circuit potential time (OCPT) measurements in 0.1 M KCl solution for 3000 s, using a commercial reference electrode as the potential reference.


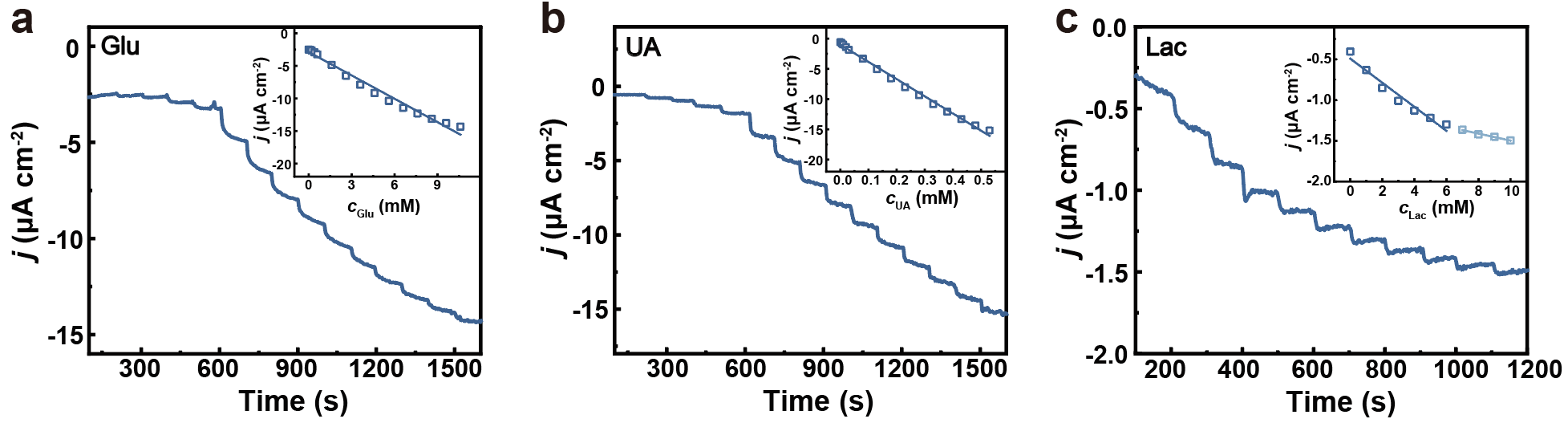


**Figure S29.** **Amperometric responses and calibration curves of enzymatic biosensors in simulated wound fluid (SWF).** (a) Glucose sensor, (b) uric acid sensor, and (c) lactate sensor showing stepwise current responses to successive analyte additions. Insets display the corresponding linear calibration curves. SWF composition: 584.4 mg NaCl, 336.0 mg NaHCO_3_, 29.8 mg KCl, 27.8 mg CaCl_2_, and 3.3 g bovine serum albumin in 100 mL DI water.


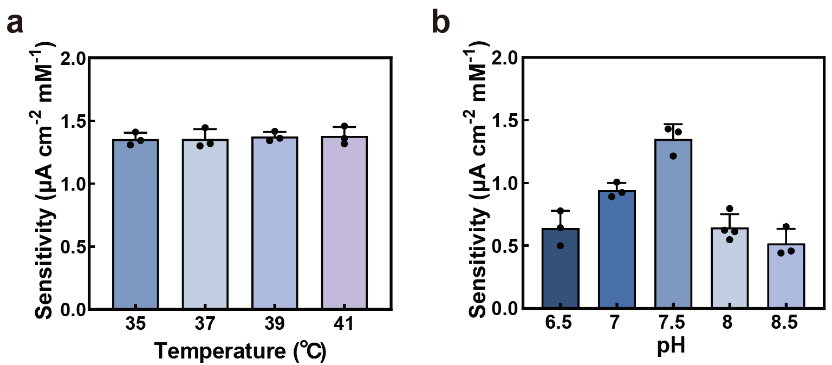


**Figure S30.** **Temp. (a, 35-41°C) and pH (b, 6.5-8.5) dependent calibration curves of the Glu sensor**, providing essential data for accurate metabolite measurements under varying wound conditions. Data are presented as mean ± SD (n = 3).


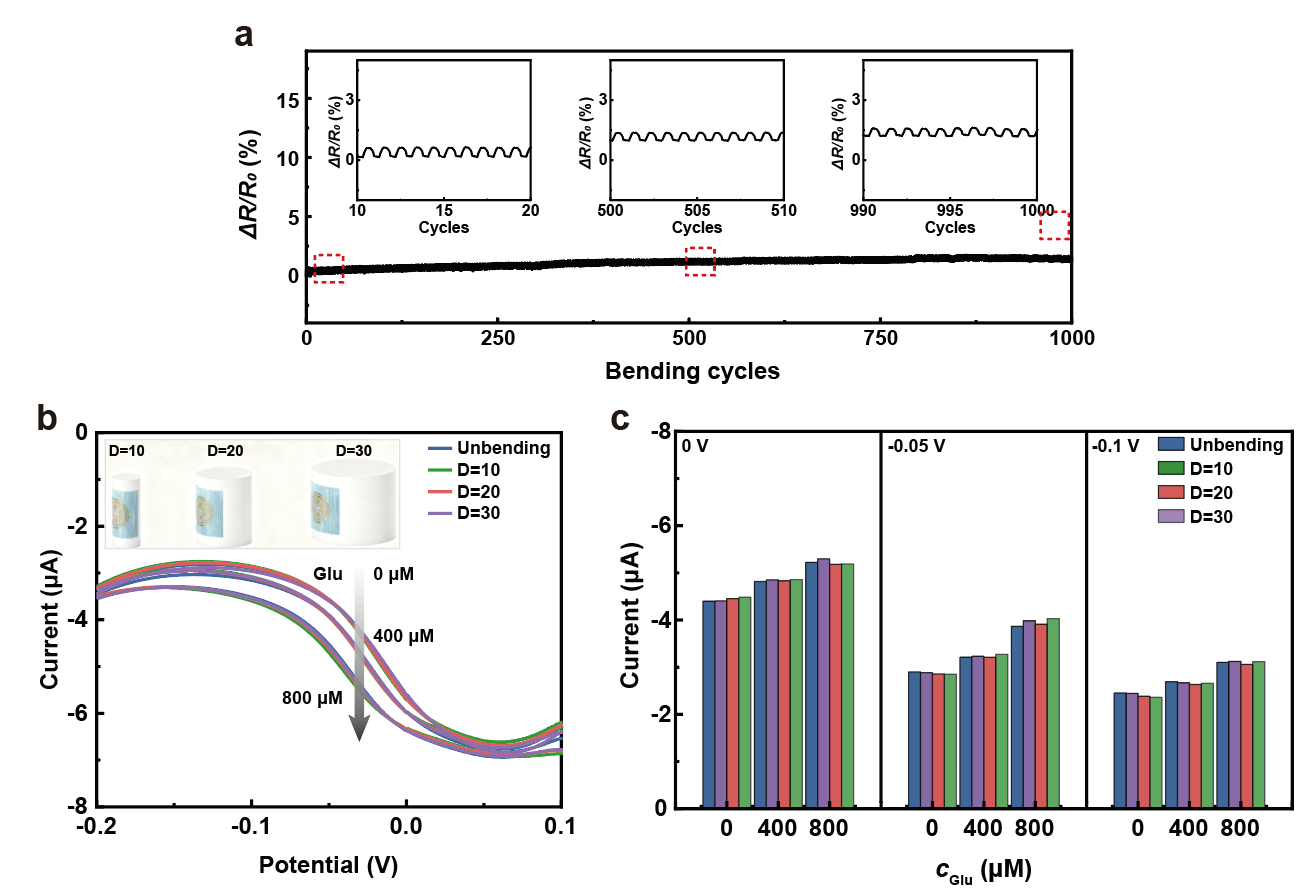


**Figure S31.** **Mechanical stability evaluation of HAST biochip.** (a) Resistance changes (Δ*R/R*_0_) of the electrode during 1,000 continuous bending cycles, where R_0_ represents initial resistance (inset: resistance measurement setup). (b) Linear Sweep Voltammetry (LSV) curves of glucose sensing under different bending diameters (D=30, 20, 10 mm) at glucose concentrations of 0, 400, and 800 μM, respectively; (c) Statistical analysis of glucose sensing response under different bending diameters at 0 V, -0.05 V, and -0.1 V, demonstrating no significant influence from bending deformation.


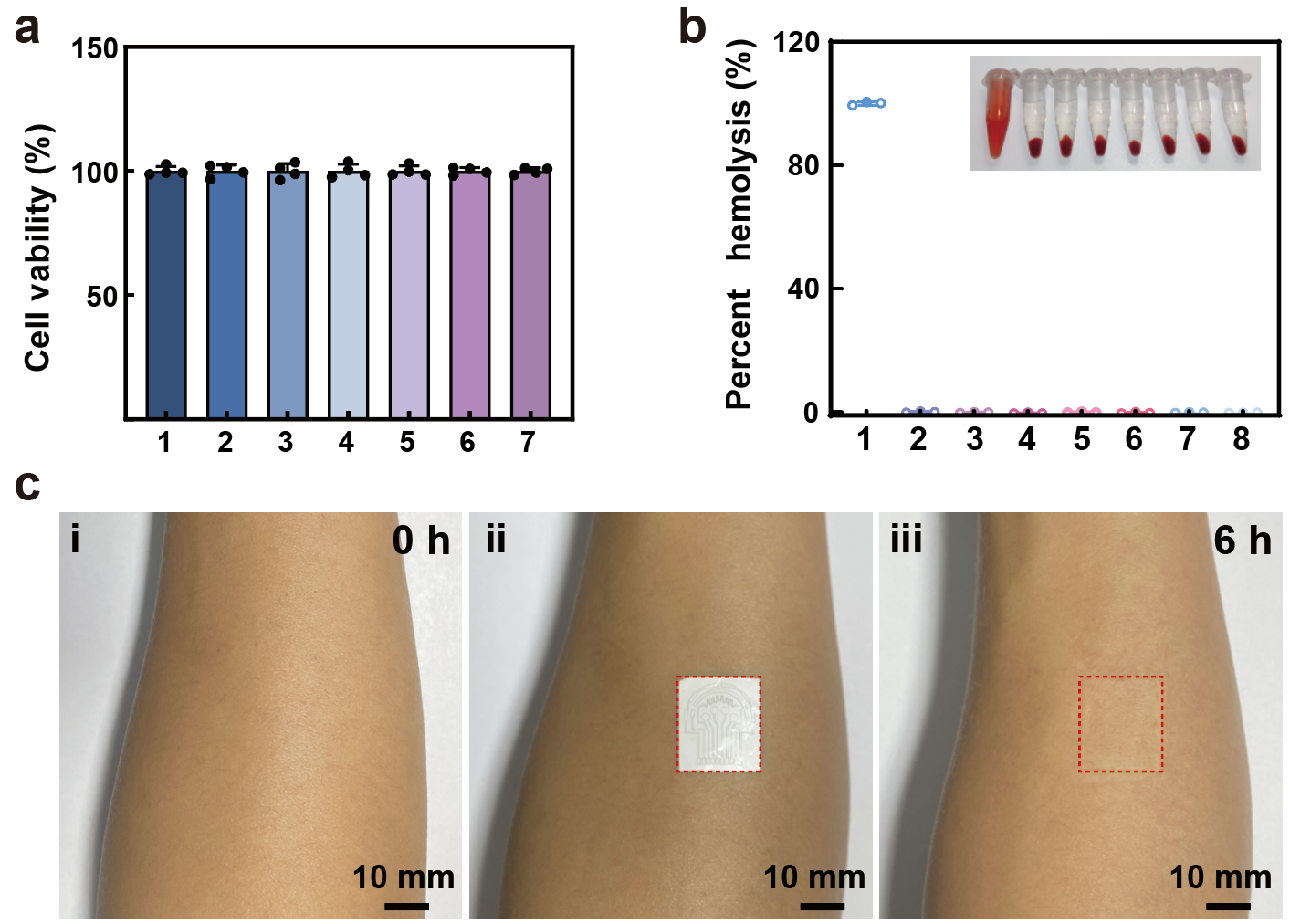


**Figure S32.** **Biocompatibility evaluation of HAST.** (a) Cell viability results (>95%) for different HAST components: (1) Blank, (2) PET, (3) PET/TPU fiber/Au, (4) PET/TPU fiber/Au/PB/CS, (5) PEDOT:PSS/PDA/Enzyme, (6) PDA/CAT/PVA + PVP/H_2_O_2_/PVA, and (7) PEDOT:PSS/PDA/PAM leachate, demonstrating excellent biocompatibility. (b) Hemolysis test results of different HAST components: (1) Positive control (Triton X-100), (2) Negative control (Permeabilization Buffer), (3) PET, (4) PET/TPU fiber/Au, (5) PET/TPU fiber/Au/PB/CS, (6) PEDOT:PSS/PDA/Enzyme, (7) PDA/CAT/PVA + PVP/H₂O₂/PVA, and (8) PEDOT:PSS/PDA/PAM leachate. Data are presented as mean ± SD (n = 3). (c) Digital photographs of HAST biochip on human skin before and after 6-hour application, showing no visible irritation or allergic reaction.


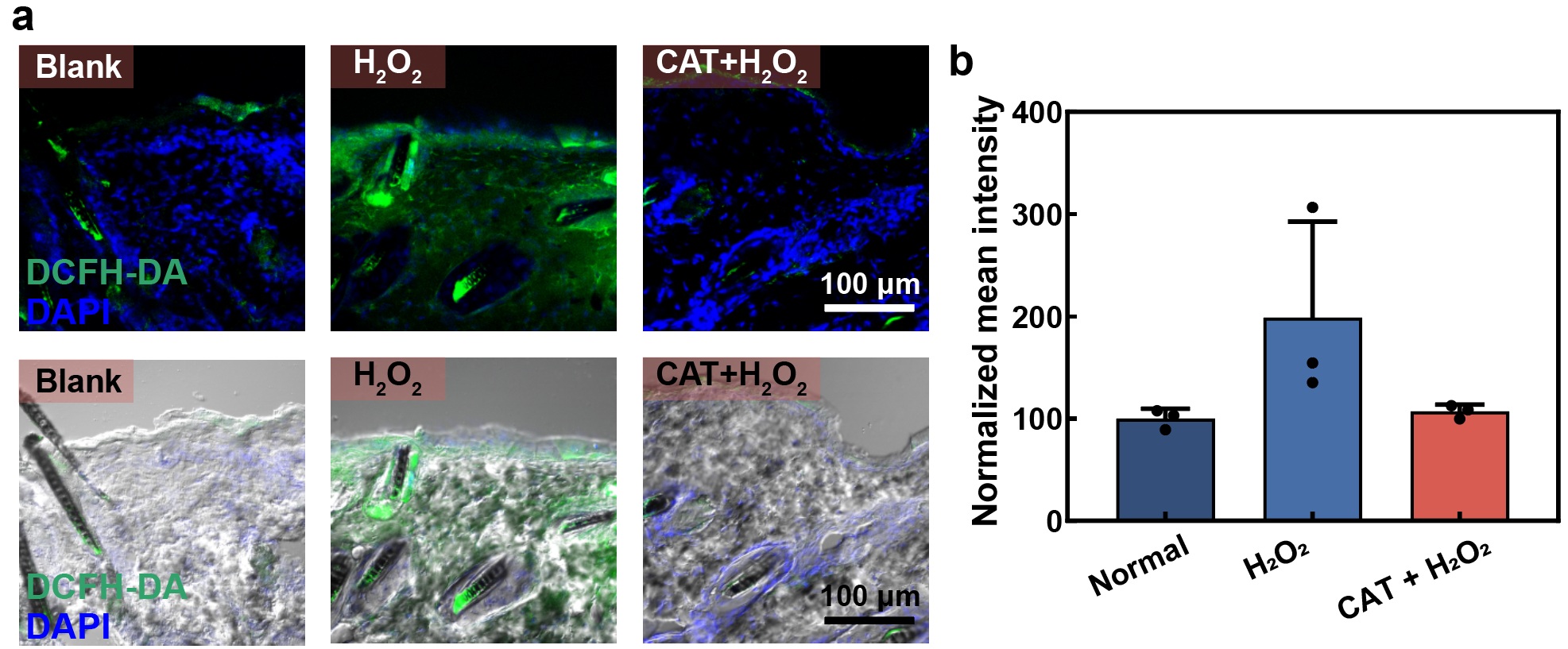


**Figure S33.** **ROS confocal fluorescence characterization.** (a) Confocal fluorescence images of mouse wound slices treated with different patches, including the Normal group, PVP/H_2_O_2_/PVA fiber group (H_2_O_2_), our O_2_ provision module with PDA/CAT/PVA and PVP/ H_2_O_2_/PVA layers (CAT + H_2_O_2_). (b) Quantification of normalized fluorescence intensity, showing that no significant oxidative stress was observed between normal wounds and wounds treated with O_2_ provision module while significant increase of oxidative stress is observed for H_2_O_2_ group. Data are presented as mean ± SD (n = 3).

**
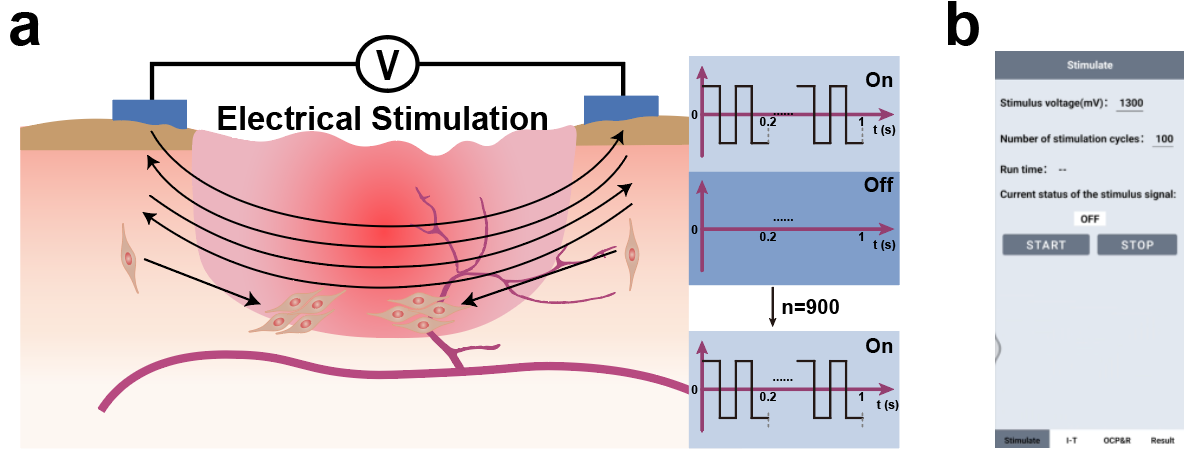
**

**Figure S34. Electrical stimulation strategy and control interface.** (a) Schematic illustration of the electrical stimulation (ES) configuration for wound therapy. Two hydrogel electrodes are positioned on the peri-wound skin to deliver a bidirectional pulsed electric field across the wound area. The stimulation protocol consists of ±1.3 V pulsed DC signals (corresponding to ~100 mV mm^-1^ across a 13 mm electrode spacing), with polarity reversal every 50 ms and an intermittent stimulation mode of 1 s ON/1 s OFF repeated for 900 cycles. (b) User interface of the control system for real-time regulation of stimulation voltage, stimulation cycles, and operational status based on biosensor feedback.


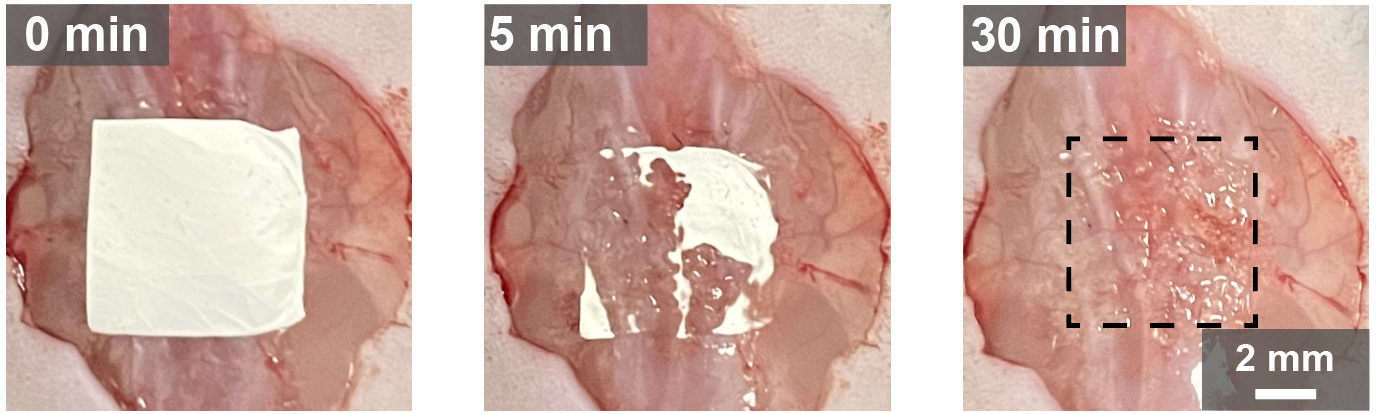


**Figure S35.** **Optical photographs of the O_2_ provision film applied to the diabetic wound of a mouse**, showing that the film gradually dissolves, which provides the possibility for oxygen generation.


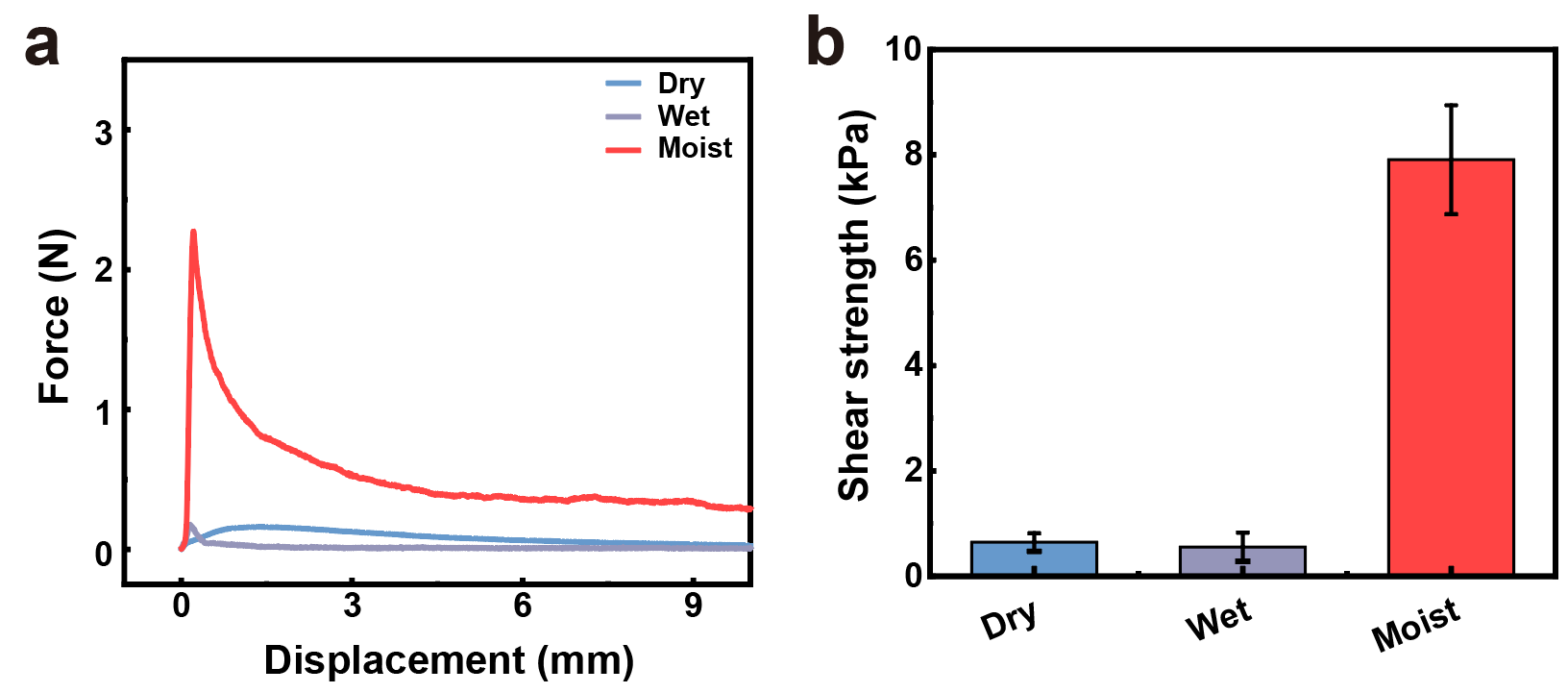


**Figure S36. Adhesion properties of oxygen-generating electrospun fibers under different hydration conditions.** (a) Representative force-displacement curves from lap shear testing of PDA/CAT/PVA and PVP/H₂O₂/PVA fibers adhered to mimic wound tissue under three conditions: dry (as-fabricated fibers), wet (fully immersed in water), and moist (simulating wound exudate exposure). (b) Quantified shear strength under different hydration states (n = 3, mean ± s.d.), showing that dissolved PVA in the moist state provides significantly enhanced adhesion strength (~7.91 kPa) compared to dry (~0.65 kPa) and wet (~0.55 kPa) states. This moisture-activated adhesion mechanism helps maintain effective fiber-tissue contact at the wound interface after exudate-triggered dissolution. Data are presented as mean ± SD (n = 3).


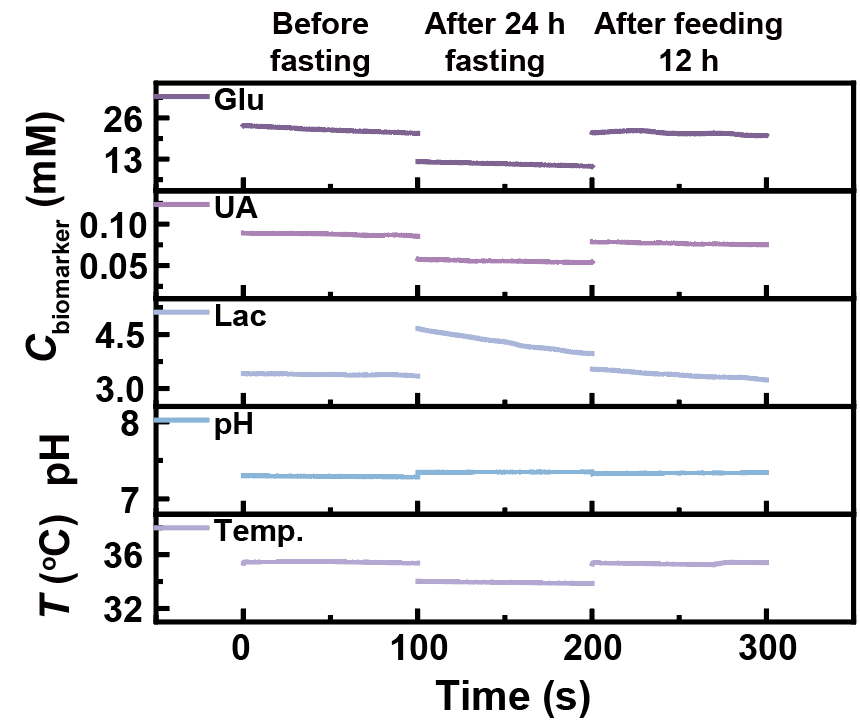


**Figure S37.** **Real-time monitoring of metabolic changes in wound exudate using HAST biochip.** Dynamic changes of glucose, uric acid, lactate, pH, and temperature were continuously tracked before fasting, after 24 h fasting, and following 12 h refeeding in diabetic mice, demonstrating HAST’s capability for real-time wound metabolism monitoring.


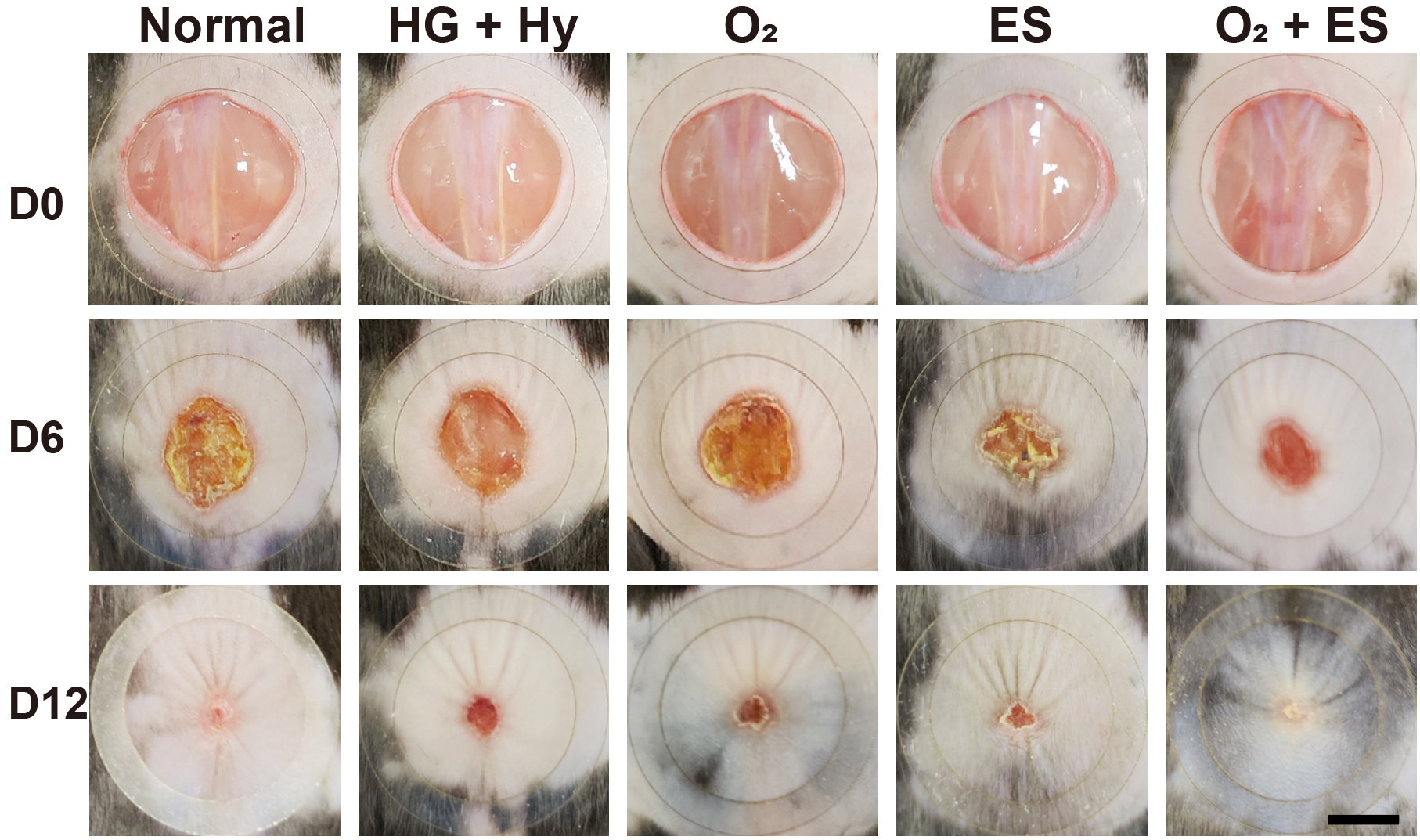


**Figure S38.** **Wound healing progression assessment over time.** Representative macroscopic images of wounds at day 0, 6, and 12 post-surgery in normal mice, untreated diabetic mice, and diabetic mice treated with O_2_, ES, or O_2_ + ES (n=3). O_2_ + ES treatment demonstrates accelerated wound closure comparable to normal healing, while untreated diabetic wounds show delayed healing. Scale bar: 5 mm.


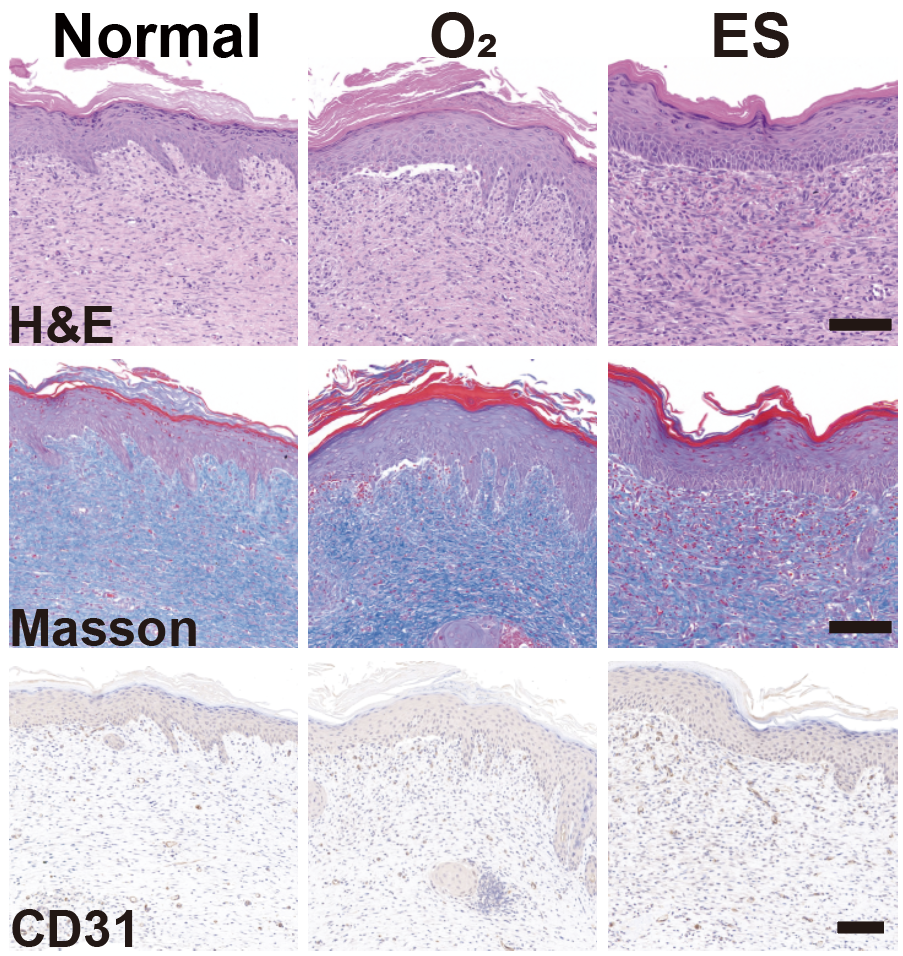


**Figure S39.** **Histological analysis of wound healing at day 12.** Representative images of H&E staining showing re-epithelialization, Masson’s trichrome staining revealing collagen deposition, and CD31 immunostaining indicating blood vessel formation in normal mice and diabetic mice treated with O_2_, or ES (n=3). Scale bar: 100 μm.


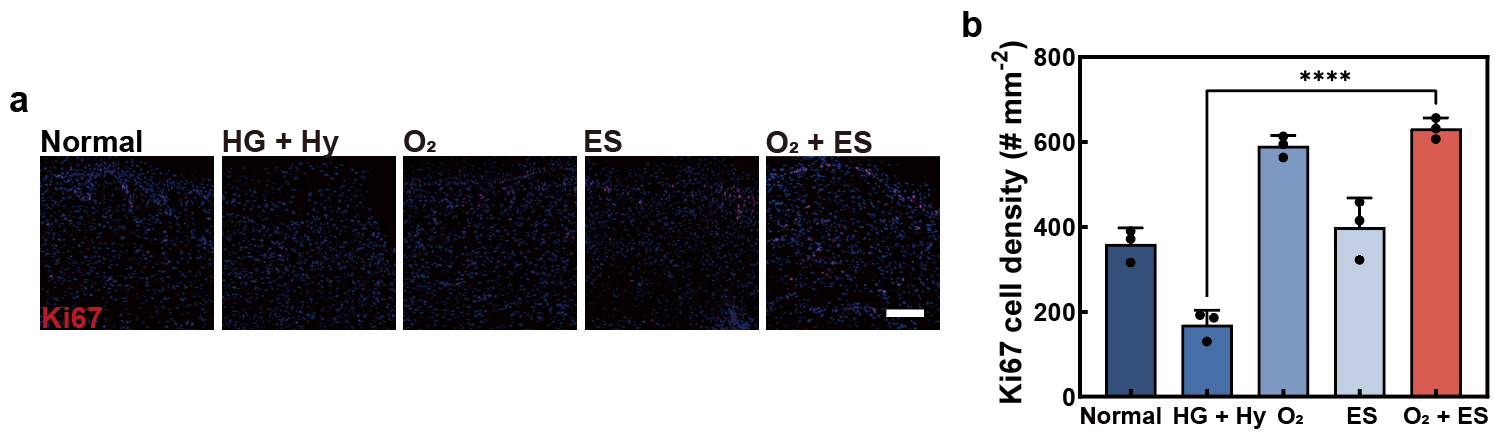


**Figure S40.** **Cell proliferation analysis in wound tissues at day 12.** (a) Representative immunofluorescence images showing Ki67-positive proliferating cells (red) in wound sections from normal mice, untreated diabetic mice, and diabetic mice treated with O_2_, ES, or O_2_ + ES (Red: Ki67, Blue: DAPI). Scale bar: 100 μm. (b) Quantification of Ki67-positive cell density in wound tissue sections across different groups, demonstrating enhanced cell proliferation in O_2_ + ES treated wounds comparable to normal healing. Data are presented as mean ± SD (n = 3). Statistical significance was determined using one-way ANOVA followed by Dunnett's multiple-comparisons test. **P < 0.01; ***P < 0.001; ****P < 0.0001.

**Table S1. Reference Ranges of Biochemical Analytes and Physical Parameters in Wound Exudate.**

| Biochemical | Normal healing  wounds | Infected nonhealing wounds | Reference |
| --- | --- | --- | --- |
| Glucose | 1.3-5.9 mM | 0-1.2 mM | ^[13-18]^ |
| Lactate | 2.0-5.9 mM | 6.1-16.7 mM | ^[13, 15, 18-20]^ |
| Uric acid | 221-751 μM | 0-200 μM | ^[13-15, 18, 21]^ |
| pH | 4.0-7.5 | 7.6-9.0 | ^[17, 19, 22-23]^ |
| Temperature | 34-37.1^o^C | 37.2-42^o^C | ^[14, 17, 19-20, 23-24]^ |

**Supplementary Video 1:** **Wireless HAST system operation in diabetic wound model**. Real-time demonstration of the HAST biochip’s in vivo performance showing secure adhesion to wound tissue, maintained functionality during normal mouse locomotion, and continuous wireless biomarker monitoring coupled with responsive electrical stimulation therapy. The video illustrates the system’s ability to operate autonomously while the animal moves freely, demonstrating practical applicability for chronic wound management.

**References**

[1] Q.-B. Zhu, B. Li, D.-D. Yang, C. Liu, S. Feng, M.-L. Chen, Y. Sun, Y.-N. Tian, X. Su, X.-M. Wang, S. Qiu, Q.-W. Li, X.-M. Li, H.-B. Zeng, H.-M. Cheng, D.-M. Sun, *Nat. Commun.* **2021**, 12, 1798.

[2] S. Chun, J.-S. Kim, Y. Yoo, Y. Choi, S. J. Jung, D. Jang, G. Lee, K.-I. Song, K. S. Nam, I. Youn, D. Son, C. Pang, Y. Jeong, H. Jung, Y.-J. Kim, B.-D. Choi, J. Kim, S.-P. Kim, W. Park, S. Park, *Nat. Electron.* **2021**, 4, 429.

[3] F. Liao, Z. Zhou, B. J. Kim, J. Chen, J. Wang, T. Wan, Y. Zhou, A. T. Hoang, C. Wang, J. Kang, J.-H. Ahn, Y. Chai, *Nat. Electron.* **2022**, 5, 84.

[4] C. Lenk, P. Hövel, K. Ved, S. Durstewitz, T. Meurer, T. Fritsch, A. Männchen, J. Küller, D. Beer, T. Ivanov, M. Ziegler, *Nat. Electron.* **2023**, 6, 370.

[5] C. Wang, Y. Bian, K. Liu, M. Qin, F. Zhang, M. Zhu, W. Shi, M. Shao, S. Shang, J. Hong, Z. Zhu, Z. Zhao, Y. Liu, Y. Guo, *Nat. Commun.* **2024**, 15, 3123.

[6] C. Zhao, J. Park, D. Maulà, Y. Yuan, D. Zhong, W. Wang, Q. Liu, C. Xu, Y. Zheng, R. K. Mow, Y. Jiang, C. Xu, H. Lyu, L. Michalek, A. Berman, Y. Jiang, S. Wei, C. Zhu, C. Wu, A. Abramson, E. Kim, X. Ji, Z. Yu, J. Shi, M. Khatib, B. Shi, Z. Bao, *Nat. Electron.* **2025**, 8, 981.

[7] H. Chen, Y. Cheng, J. Tian, P. Yang, X. Zhang, Y. Chen, Y. Hu, J. Wu, *Sci. Adv.* **2020**, 6, eaba4311.

[8] Y. Guan, H. Niu, Z. Liu, Y. Dang, J. Shen, M. Zayed, L. Ma, J. Guan, *Sci. Adv.* **2021**, 7, eabj0153.

[9] G. Chen, F. Wang, X. Zhang, Y. Shang, Y. Zhao, *Sci. Adv.* **2023**, 9, eadg3478.

[10] J. Yang, X. Jin, W. Liu, W. Wang, *Adv. Mater.* **2023**, 35, 2305819.

[11] X. Han, C. Saengow, L. Ju, W. Ren, R. H. Ewoldt, J. Irudayaraj, *Nat. Commun.* **2024**, 15, 3435.

[12] X. Han, A. Bushra, W. Ren, R. Lu, D. Um, L. Ju, T. Jensen, S. Paul, M. Mahoney, M. Tsipursky, G. Cheng, J. Irudayaraj, *ACS Nano* **2025**, DOI: 10.1021/acsnano.5c13124.

[13] N. J. Trengove, S. R. Langton, M. C. Stacey, *Wound Repair Regen.* **1996**, 4, 234.

[14] C. Wang, E. Shirzaei Sani, W. Gao, *Adv. Funct. Mater.* **2022**, 32, 2111022.

[15] J. R. Sempionatto, J. A. Lasalde-Ramírez, K. Mahato, J. Wang, W. Gao, *Nat. Rev. Chem.* **2022**, 6, 899.

[16] Q. Pang, F. Yang, Z. Jiang, K. Wu, R. Hou, Y. Zhu, *Materials & Design* **2023**, 229, 111917.

[17] D. Prakashan, A. Kaushik, S. Gandhi, *Chem. Eng. J.* **2024**, 497, 154371.

[18] X. Wang, B. Zhong, Z. Lou, W. Han, L. Wang, *Chem. Eng. J.* **2024**, 484, 149643.

[19] W. Deng, M. Sun, M. Cao, C.-B. Ma, X. Bo, J. Bai, M. Zhou, *ACS Nano* **2025**, 19, 16163.

[20] E. Shirzaei Sani, C. Xu, C. Wang, Y. Song, J. Min, J. Tu, S. A. Solomon, J. Li, J. L. Banks, D. G. Armstrong, W. Gao, *Sci. Adv.* **2023**, 9, eadf7388.

[21] X. T. Zheng, Z. Yang, L. Sutarlie, M. Thangaveloo, Y. Yu, N. A. B. M. Salleh, J. S. Chin, Z. Xiong, D. L. Becker, X. J. Loh, B. C. K. Tee, X. Su, *Sci. Adv.* **2023**, 9, eadg6670.

[22] A. McLister, J. McHugh, J. Cundell, J. Davis, *Adv. Mater.* **2016**, 28, 5732.

[23] S. Darvishi, S. Tavakoli, M. Kharaziha, H. H. Girault, C. F. Kaminski, I. Mela, *Angew. Chem. Int. Ed.* **2022**, 61, e202112218.

[24] Z. Ge, W. Guo, Y. Tao, H. Sun, X. Meng, L. Cao, S. Zhang, W. Liu, M. L. Akhtar, Y. Li, Y. Ren, *Adv. Mater.* **2023**, 35, 2304005.
